# Supplementary figures and images for: Mining transcriptomic data to study the origins and evolution of a plant allopolyploid complex
Source: PeerJ. 2014 May 20;2:e391. doi: 10.7717/peerj.391 (PMC4034613; doi:10.7717/peerj.391)

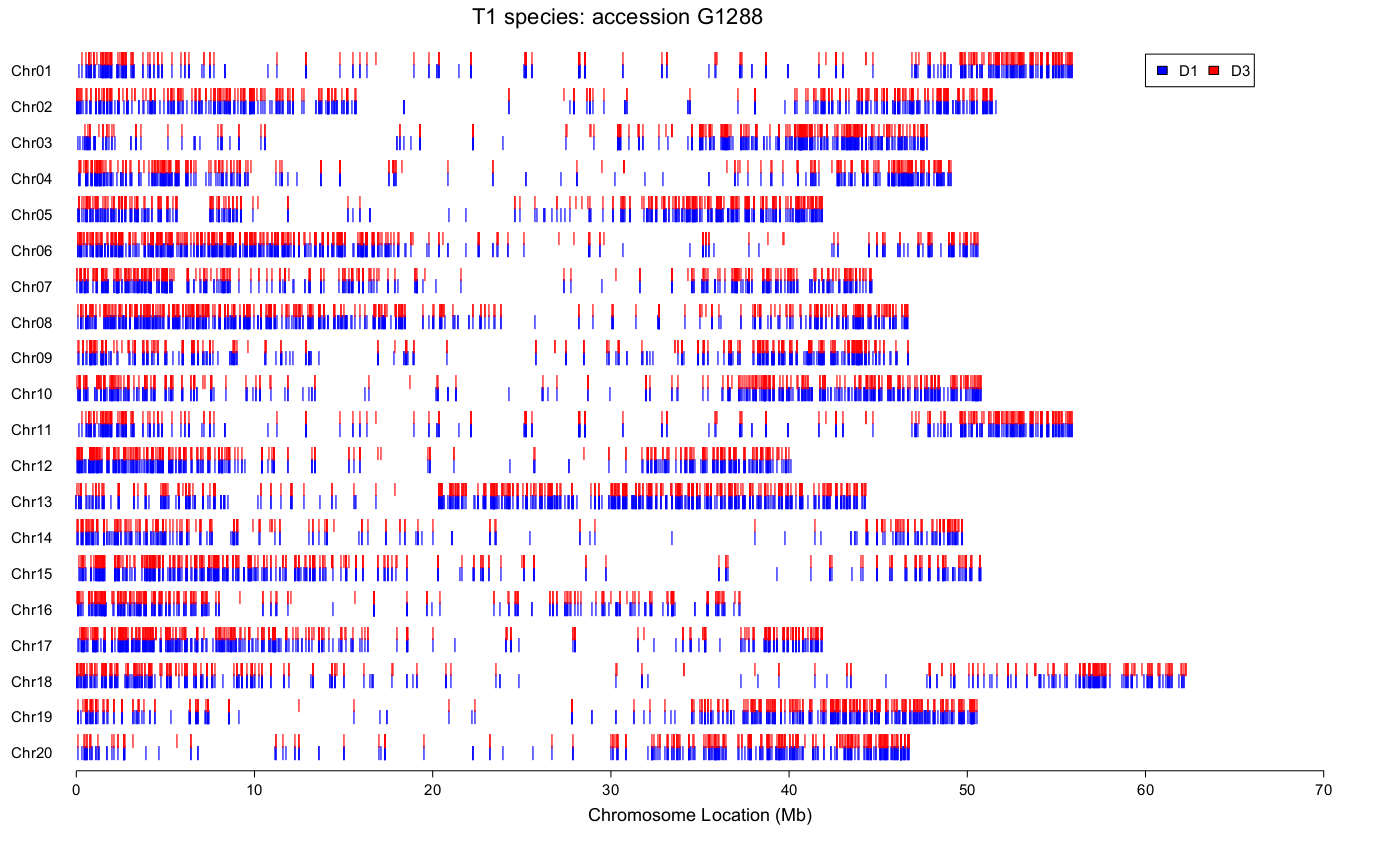

Supplement: Figures S01--S10 — Electronic chromosome painting for G. tomentella T1, accessions 1288 (Figure S01), 1361 (Figure S02), 1763 (Figure S03), G. dolichocarpa, accessions 1188 (Figure S04), 1286 (Figure S05), 1393 (Figure S06), 1854 (Figure S07) and G. tomentella T5 accessions A58-1 (Figure S08), 1487 (Figure S09) and 1969 (Figure S10). [file peerj-02-391-s001.zip › Suplementary_Figures_S01-S10/Suplementary_Figure_01.png]

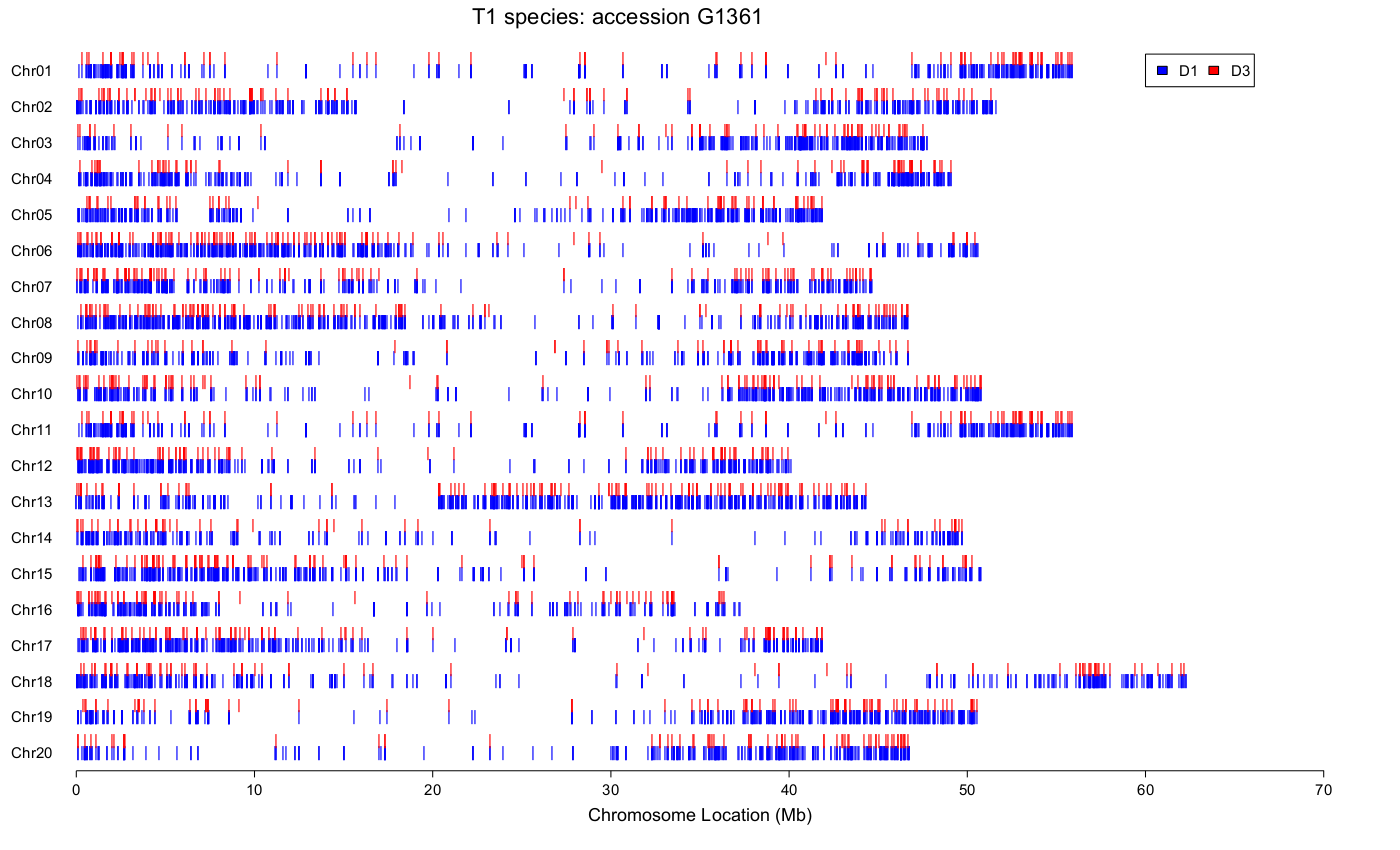

Supplement: Figures S01--S10 — Electronic chromosome painting for G. tomentella T1, accessions 1288 (Figure S01), 1361 (Figure S02), 1763 (Figure S03), G. dolichocarpa, accessions 1188 (Figure S04), 1286 (Figure S05), 1393 (Figure S06), 1854 (Figure S07) and G. tomentella T5 accessions A58-1 (Figure S08), 1487 (Figure S09) and 1969 (Figure S10). [file peerj-02-391-s001.zip › Suplementary_Figures_S01-S10/Suplementary_Figure_02.png]

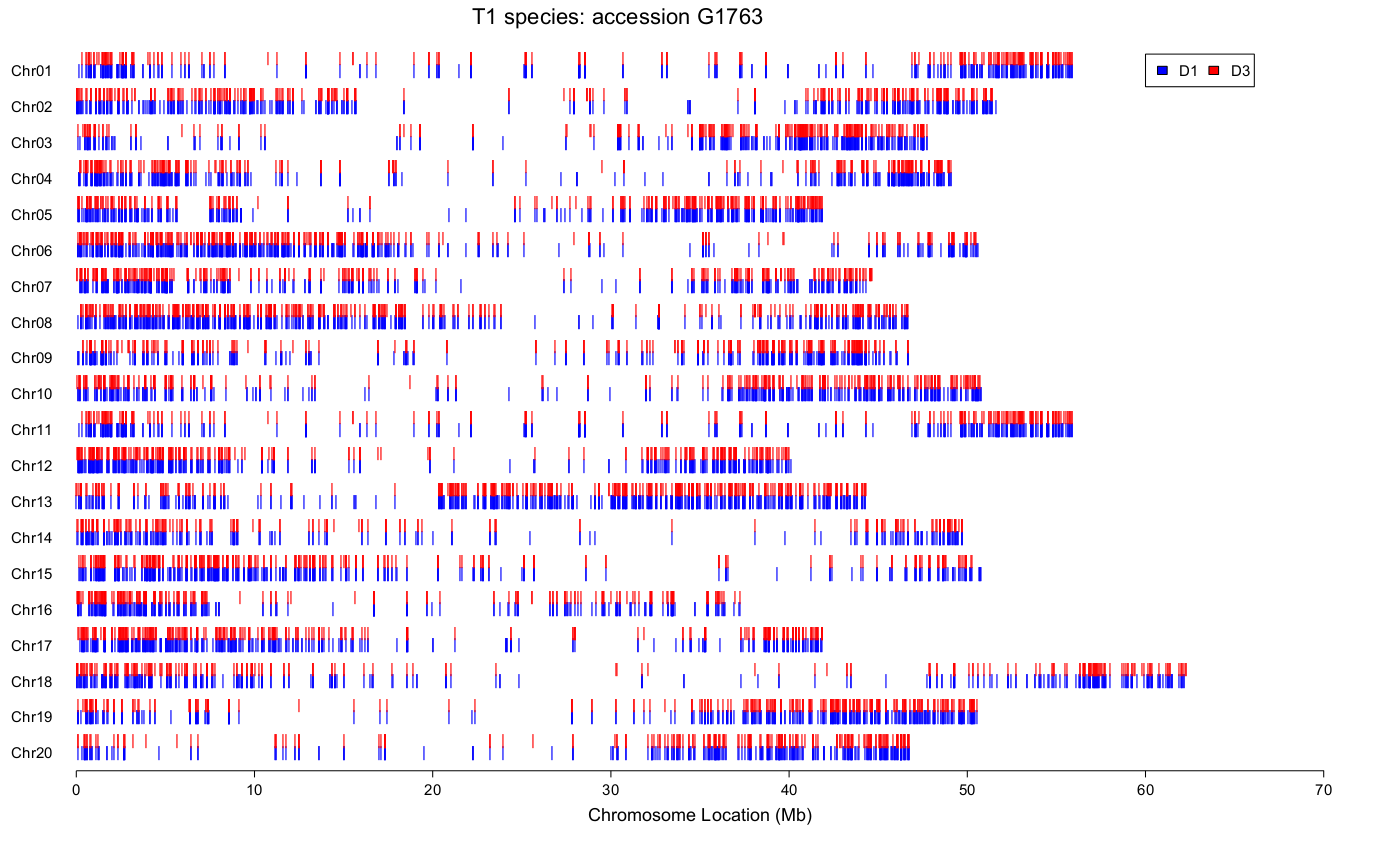

Supplement: Figures S01--S10 — Electronic chromosome painting for G. tomentella T1, accessions 1288 (Figure S01), 1361 (Figure S02), 1763 (Figure S03), G. dolichocarpa, accessions 1188 (Figure S04), 1286 (Figure S05), 1393 (Figure S06), 1854 (Figure S07) and G. tomentella T5 accessions A58-1 (Figure S08), 1487 (Figure S09) and 1969 (Figure S10). [file peerj-02-391-s001.zip › Suplementary_Figures_S01-S10/Suplementary_Figure_03.png]

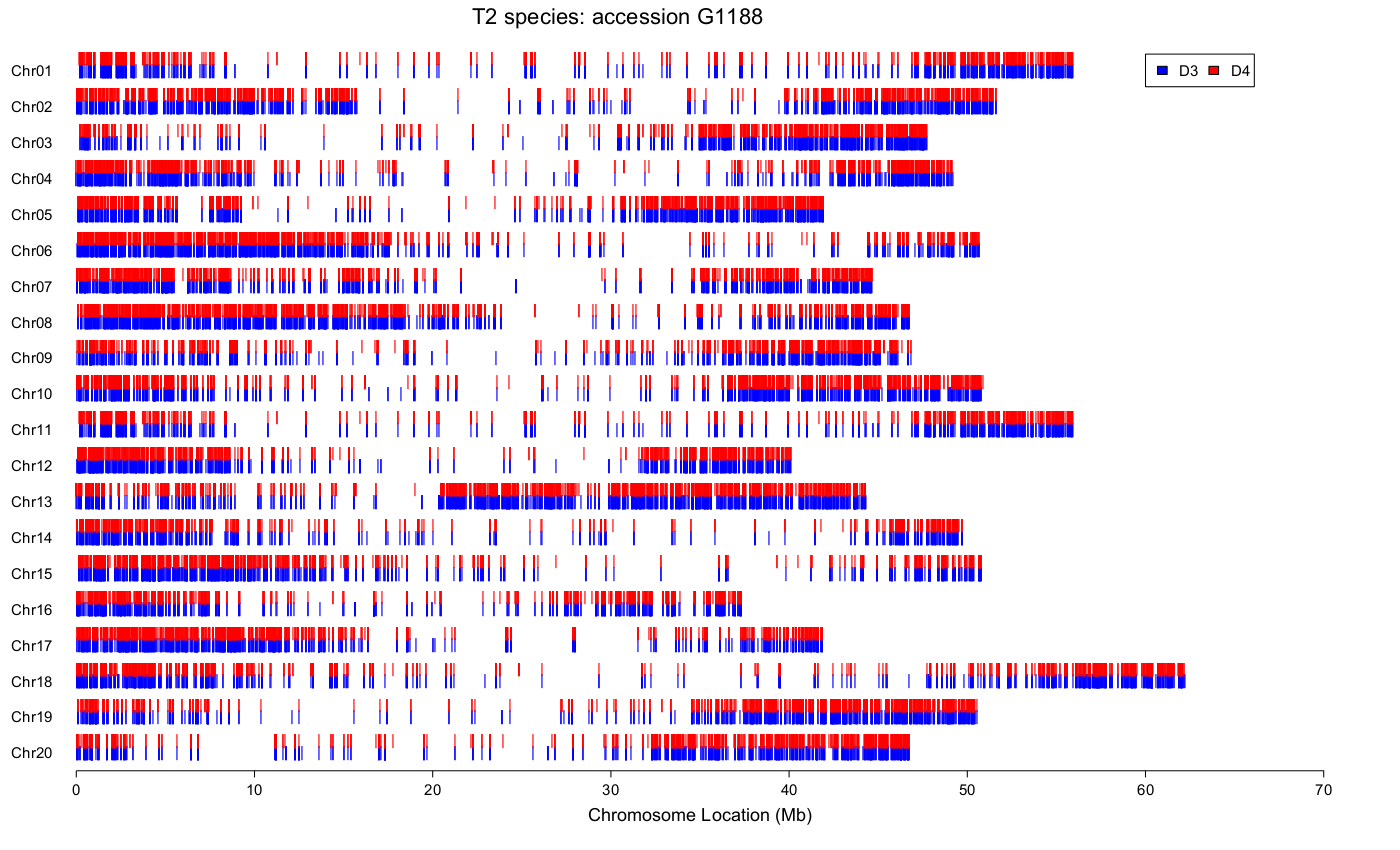

Supplement: Figures S01--S10 — Electronic chromosome painting for G. tomentella T1, accessions 1288 (Figure S01), 1361 (Figure S02), 1763 (Figure S03), G. dolichocarpa, accessions 1188 (Figure S04), 1286 (Figure S05), 1393 (Figure S06), 1854 (Figure S07) and G. tomentella T5 accessions A58-1 (Figure S08), 1487 (Figure S09) and 1969 (Figure S10). [file peerj-02-391-s001.zip › Suplementary_Figures_S01-S10/Suplementary_Figure_04.png]

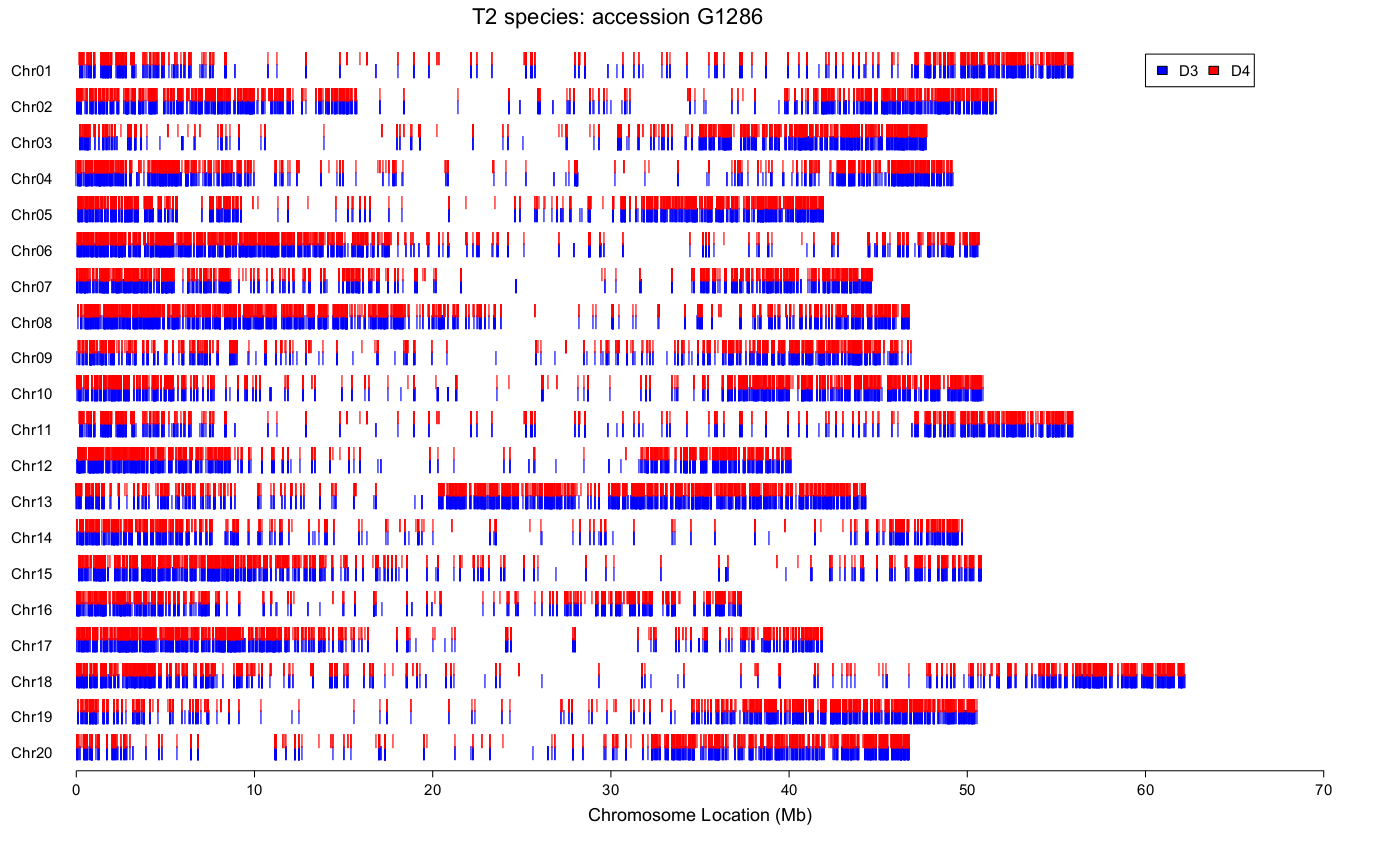

Supplement: Figures S01--S10 — Electronic chromosome painting for G. tomentella T1, accessions 1288 (Figure S01), 1361 (Figure S02), 1763 (Figure S03), G. dolichocarpa, accessions 1188 (Figure S04), 1286 (Figure S05), 1393 (Figure S06), 1854 (Figure S07) and G. tomentella T5 accessions A58-1 (Figure S08), 1487 (Figure S09) and 1969 (Figure S10). [file peerj-02-391-s001.zip › Suplementary_Figures_S01-S10/Suplementary_Figure_05.png]

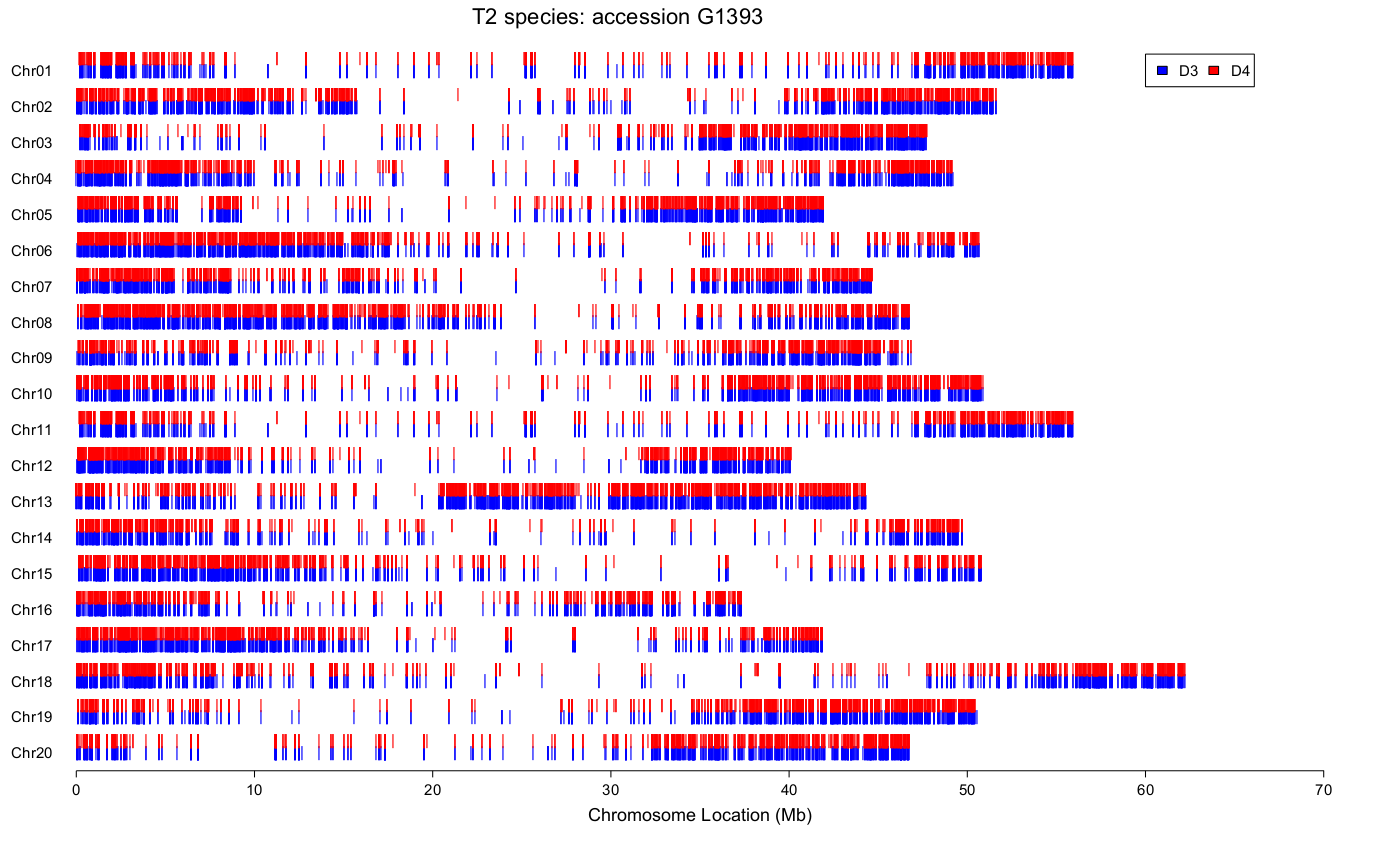

Supplement: Figures S01--S10 — Electronic chromosome painting for G. tomentella T1, accessions 1288 (Figure S01), 1361 (Figure S02), 1763 (Figure S03), G. dolichocarpa, accessions 1188 (Figure S04), 1286 (Figure S05), 1393 (Figure S06), 1854 (Figure S07) and G. tomentella T5 accessions A58-1 (Figure S08), 1487 (Figure S09) and 1969 (Figure S10). [file peerj-02-391-s001.zip › Suplementary_Figures_S01-S10/Suplementary_Figure_06.png]

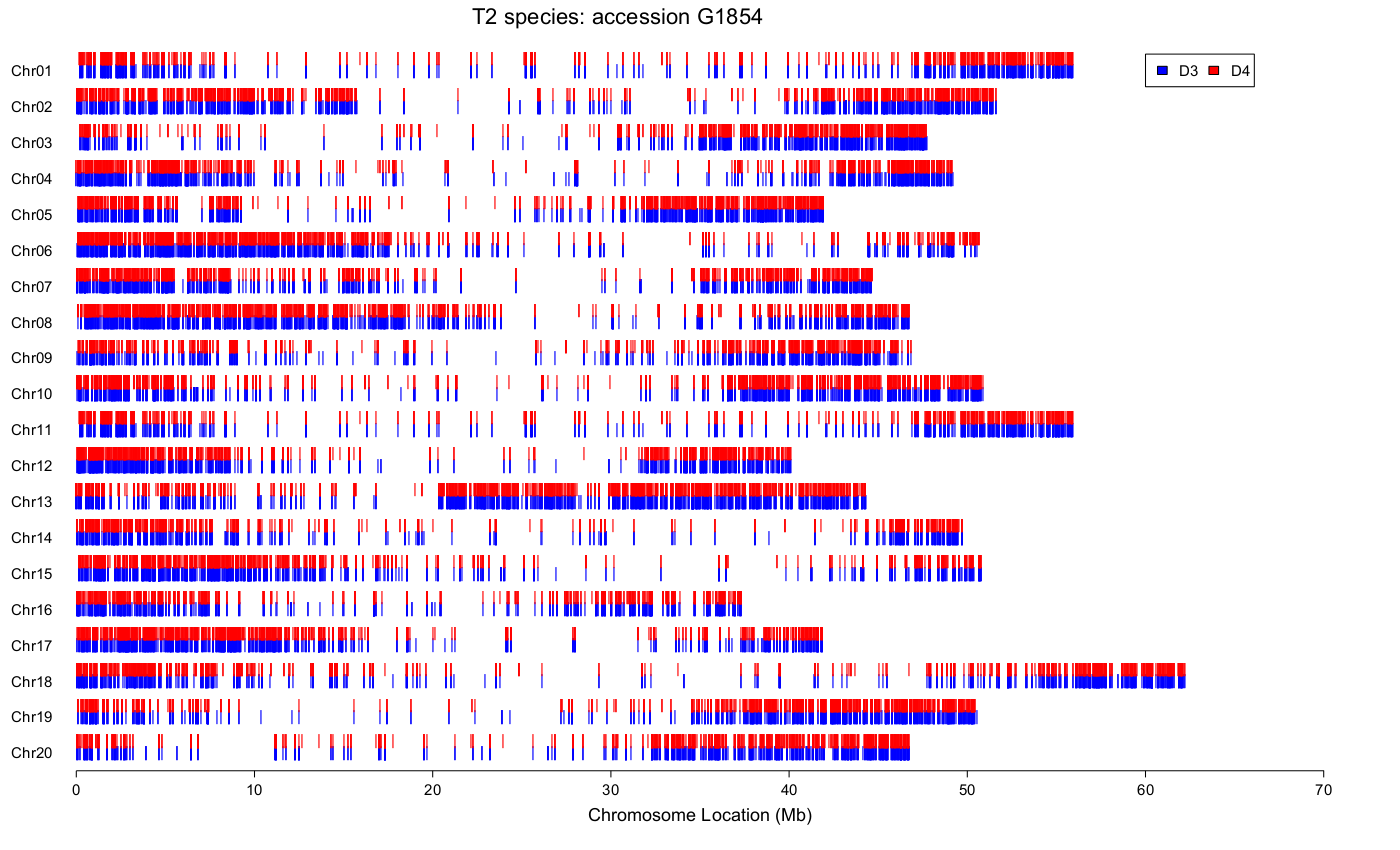

Supplement: Figures S01--S10 — Electronic chromosome painting for G. tomentella T1, accessions 1288 (Figure S01), 1361 (Figure S02), 1763 (Figure S03), G. dolichocarpa, accessions 1188 (Figure S04), 1286 (Figure S05), 1393 (Figure S06), 1854 (Figure S07) and G. tomentella T5 accessions A58-1 (Figure S08), 1487 (Figure S09) and 1969 (Figure S10). [file peerj-02-391-s001.zip › Suplementary_Figures_S01-S10/Suplementary_Figure_07.png]

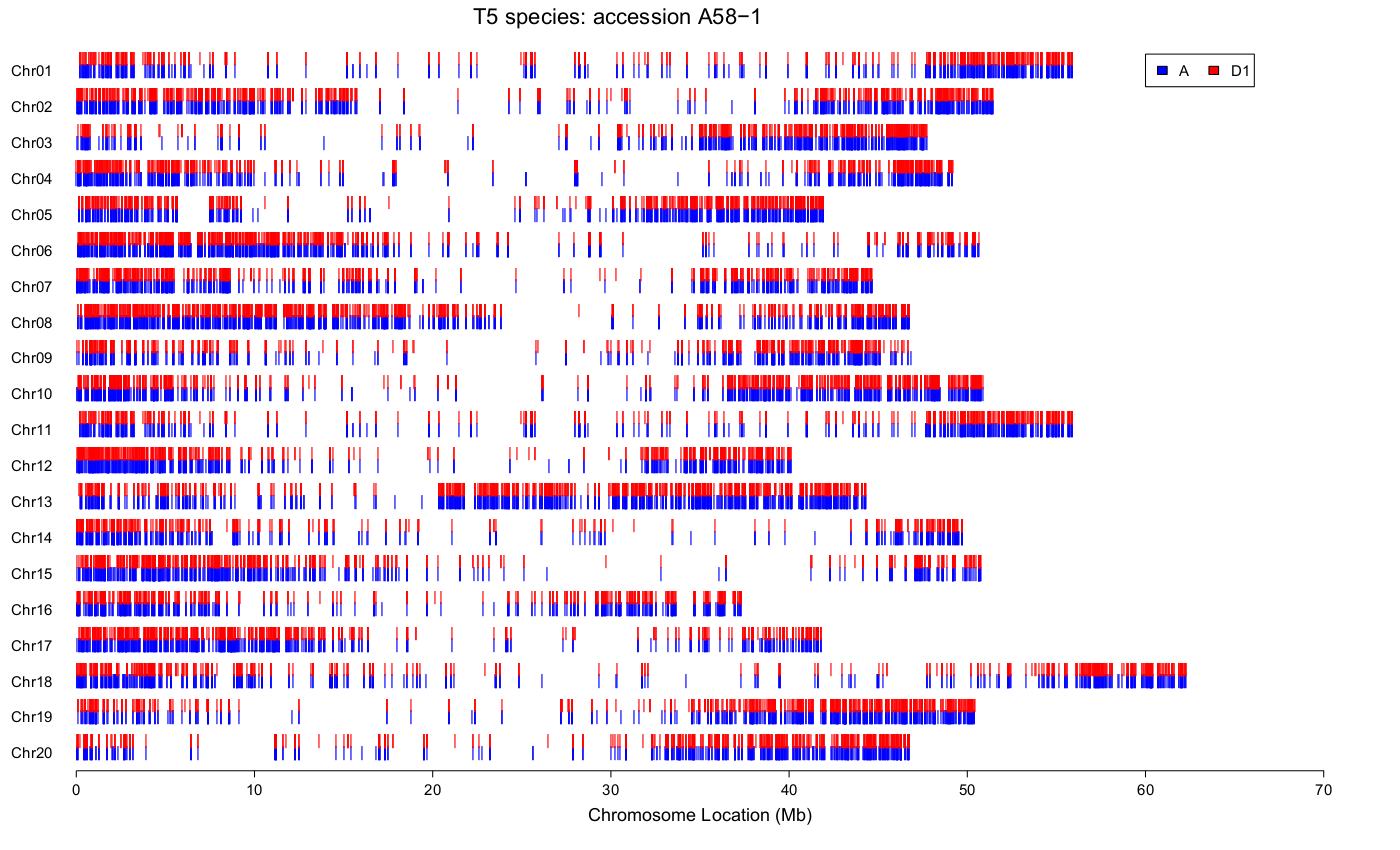

Supplement: Figures S01--S10 — Electronic chromosome painting for G. tomentella T1, accessions 1288 (Figure S01), 1361 (Figure S02), 1763 (Figure S03), G. dolichocarpa, accessions 1188 (Figure S04), 1286 (Figure S05), 1393 (Figure S06), 1854 (Figure S07) and G. tomentella T5 accessions A58-1 (Figure S08), 1487 (Figure S09) and 1969 (Figure S10). [file peerj-02-391-s001.zip › Suplementary_Figures_S01-S10/Suplementary_Figure_08.png]

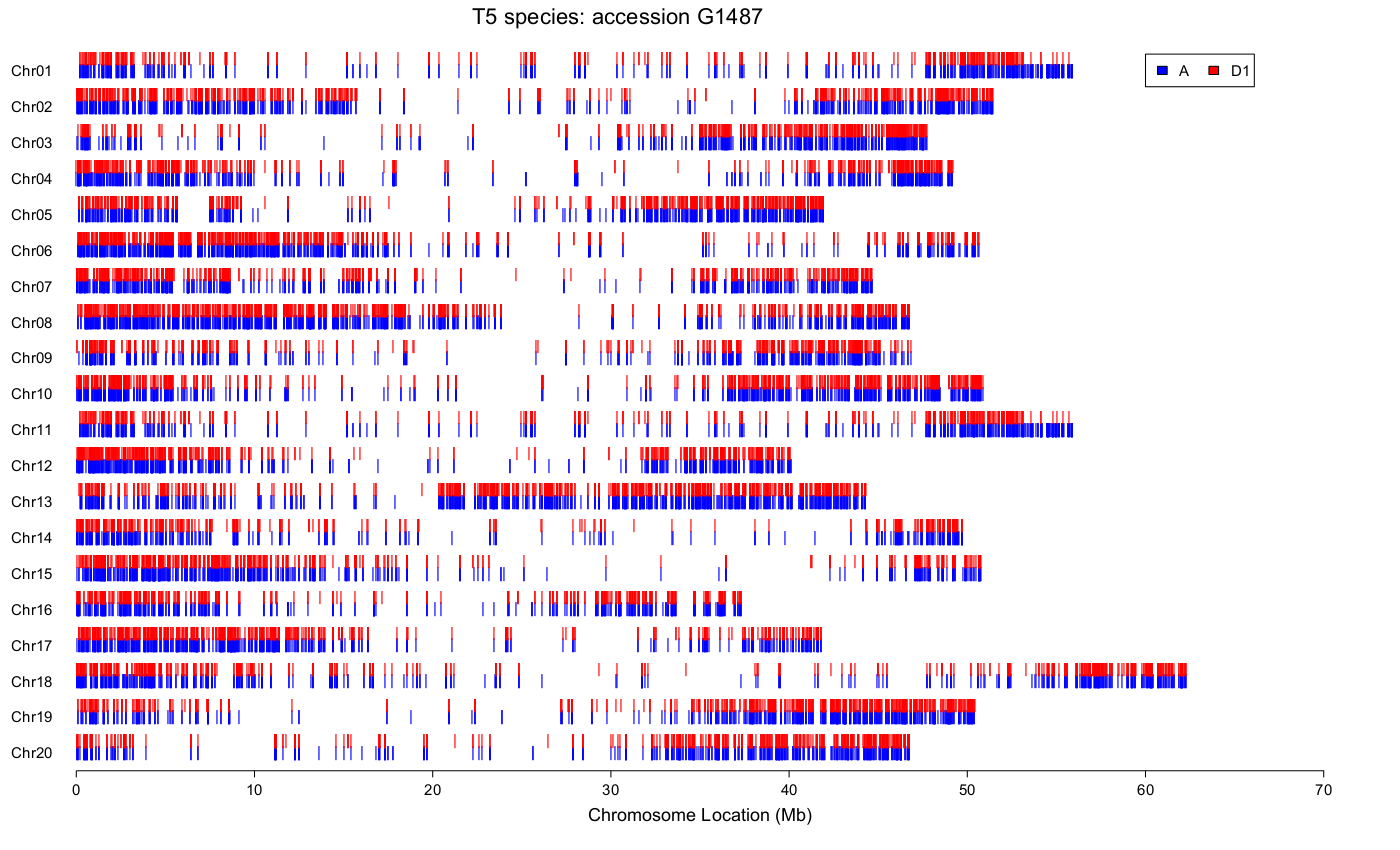

Supplement: Figures S01--S10 — Electronic chromosome painting for G. tomentella T1, accessions 1288 (Figure S01), 1361 (Figure S02), 1763 (Figure S03), G. dolichocarpa, accessions 1188 (Figure S04), 1286 (Figure S05), 1393 (Figure S06), 1854 (Figure S07) and G. tomentella T5 accessions A58-1 (Figure S08), 1487 (Figure S09) and 1969 (Figure S10). [file peerj-02-391-s001.zip › Suplementary_Figures_S01-S10/Suplementary_Figure_09.png]

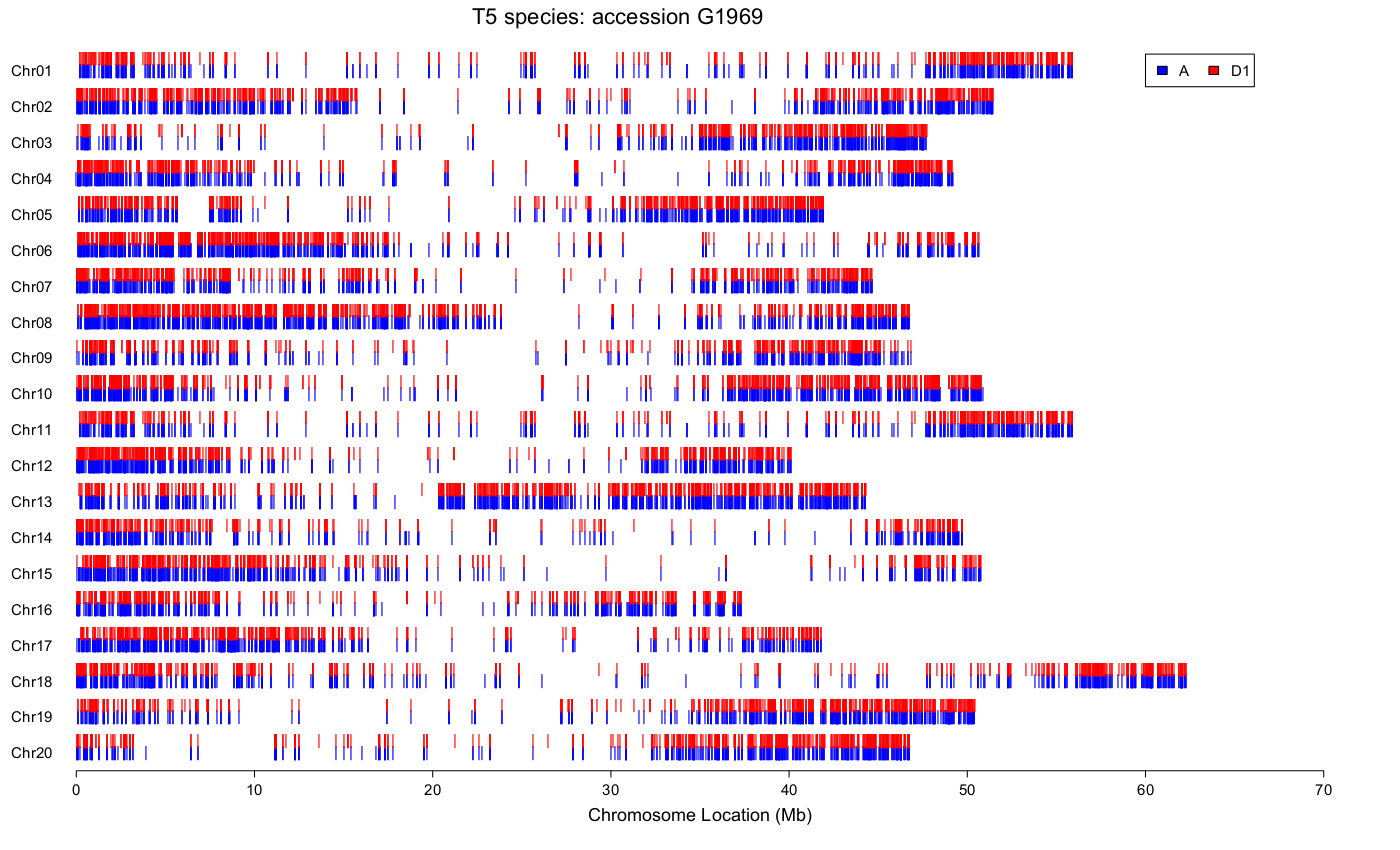

Supplement: Figures S01--S10 — Electronic chromosome painting for G. tomentella T1, accessions 1288 (Figure S01), 1361 (Figure S02), 1763 (Figure S03), G. dolichocarpa, accessions 1188 (Figure S04), 1286 (Figure S05), 1393 (Figure S06), 1854 (Figure S07) and G. tomentella T5 accessions A58-1 (Figure S08), 1487 (Figure S09) and 1969 (Figure S10). [file peerj-02-391-s001.zip › Suplementary_Figures_S01-S10/Suplementary_Figure_10.png]

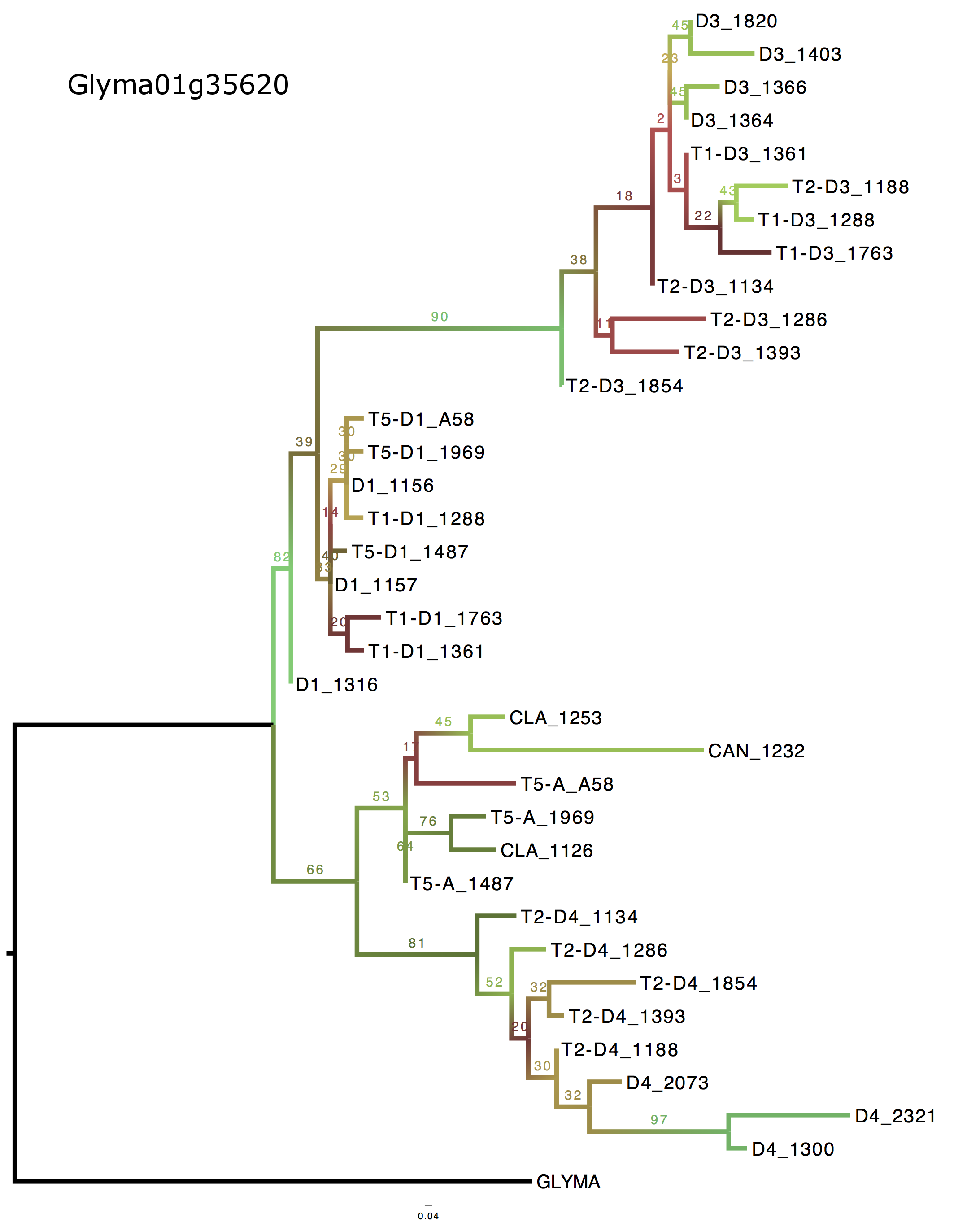

Supplement: Figures S13--S39 — MaximuThe color of the tree branch represents the bootstraping value (also with the same color). Genes are in the following order: Glyma01g35620 (Figure S13), Glyma02g11580 (Figure S14), Glyma03g29330 (Figure S15), Glyma03g36630 (Figure S16), Glyma04g39670 (Figure S17), Glyma05g05750 (Figure S18), Glyma05g09310 (Figure S19), Glyma05g26230 (Figure S20), Glyma05g37840 (Figure S21), Glyma06g18640 (Figure S22), Glyma07g03370 (Figure S23), Glyma07g17180 (Figure S24), Glyma10g42100 (Figure S25), Glyma11g13880 (Figure S26), Glyma11g33720 (Figure S27), Glyma12g04150 (Figure S28), Glyma12g12230 (Figure S29), Glyma13g17820 (Figure S30), Glyma14g03500 (Figure S31), Glyma16g00410 (Figure S32), Glyma16g01980 (Figure S33), Glyma16g04940 (Figure S34), Glyma18g04080 (Figure S35), Glyma19g03390 (Figure S36), Glyma19g32940 (Figure S37), Glyma20g24930 (Figure S38), Glyma20g32930 (Figure S39) [file peerj-02-391-s002.zip › Suplementary_Figures_S13-S39/Suplementary_Figure_13.png]

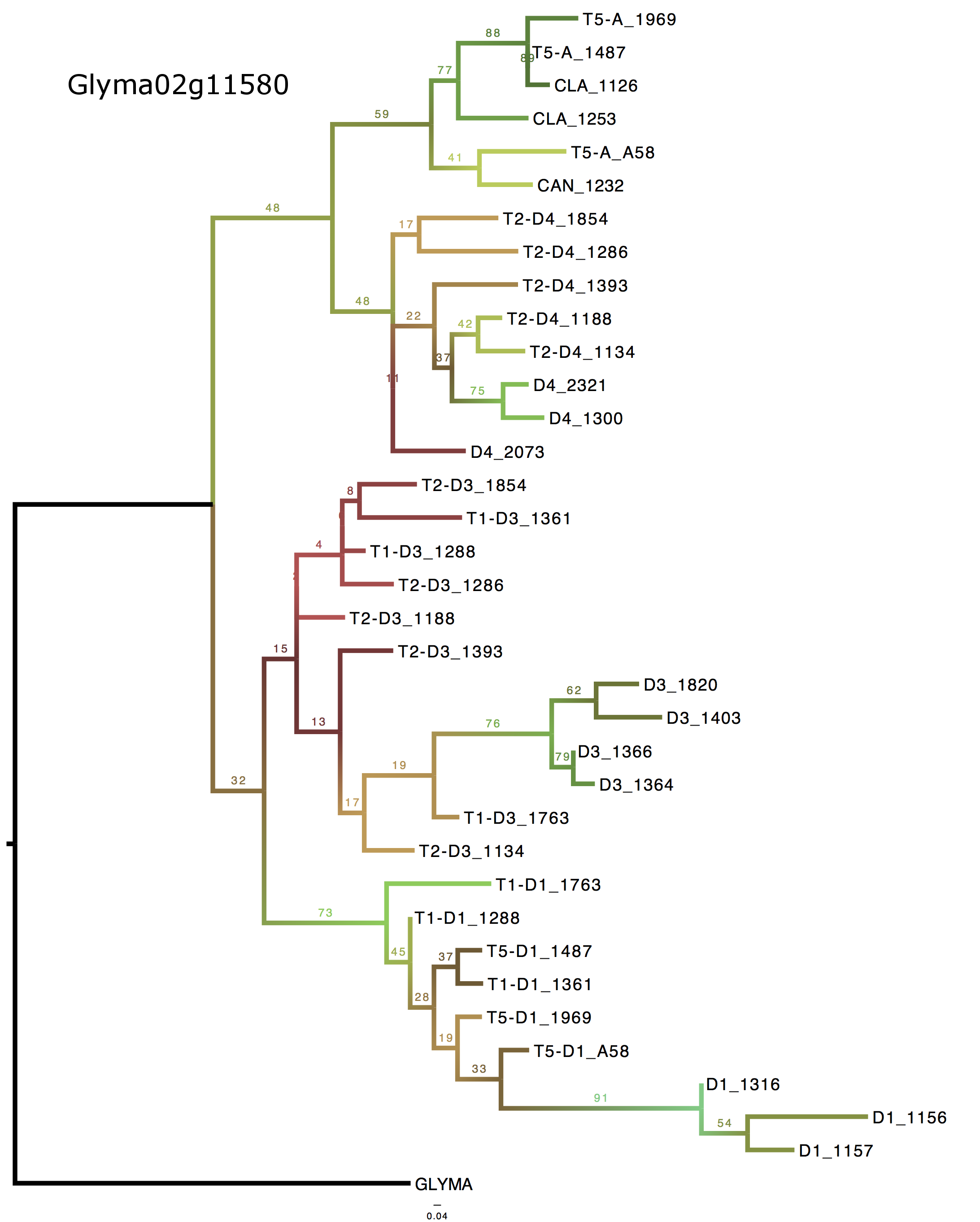

Supplement: Figures S13--S39 — MaximuThe color of the tree branch represents the bootstraping value (also with the same color). Genes are in the following order: Glyma01g35620 (Figure S13), Glyma02g11580 (Figure S14), Glyma03g29330 (Figure S15), Glyma03g36630 (Figure S16), Glyma04g39670 (Figure S17), Glyma05g05750 (Figure S18), Glyma05g09310 (Figure S19), Glyma05g26230 (Figure S20), Glyma05g37840 (Figure S21), Glyma06g18640 (Figure S22), Glyma07g03370 (Figure S23), Glyma07g17180 (Figure S24), Glyma10g42100 (Figure S25), Glyma11g13880 (Figure S26), Glyma11g33720 (Figure S27), Glyma12g04150 (Figure S28), Glyma12g12230 (Figure S29), Glyma13g17820 (Figure S30), Glyma14g03500 (Figure S31), Glyma16g00410 (Figure S32), Glyma16g01980 (Figure S33), Glyma16g04940 (Figure S34), Glyma18g04080 (Figure S35), Glyma19g03390 (Figure S36), Glyma19g32940 (Figure S37), Glyma20g24930 (Figure S38), Glyma20g32930 (Figure S39) [file peerj-02-391-s002.zip › Suplementary_Figures_S13-S39/Suplementary_Figure_14.png]

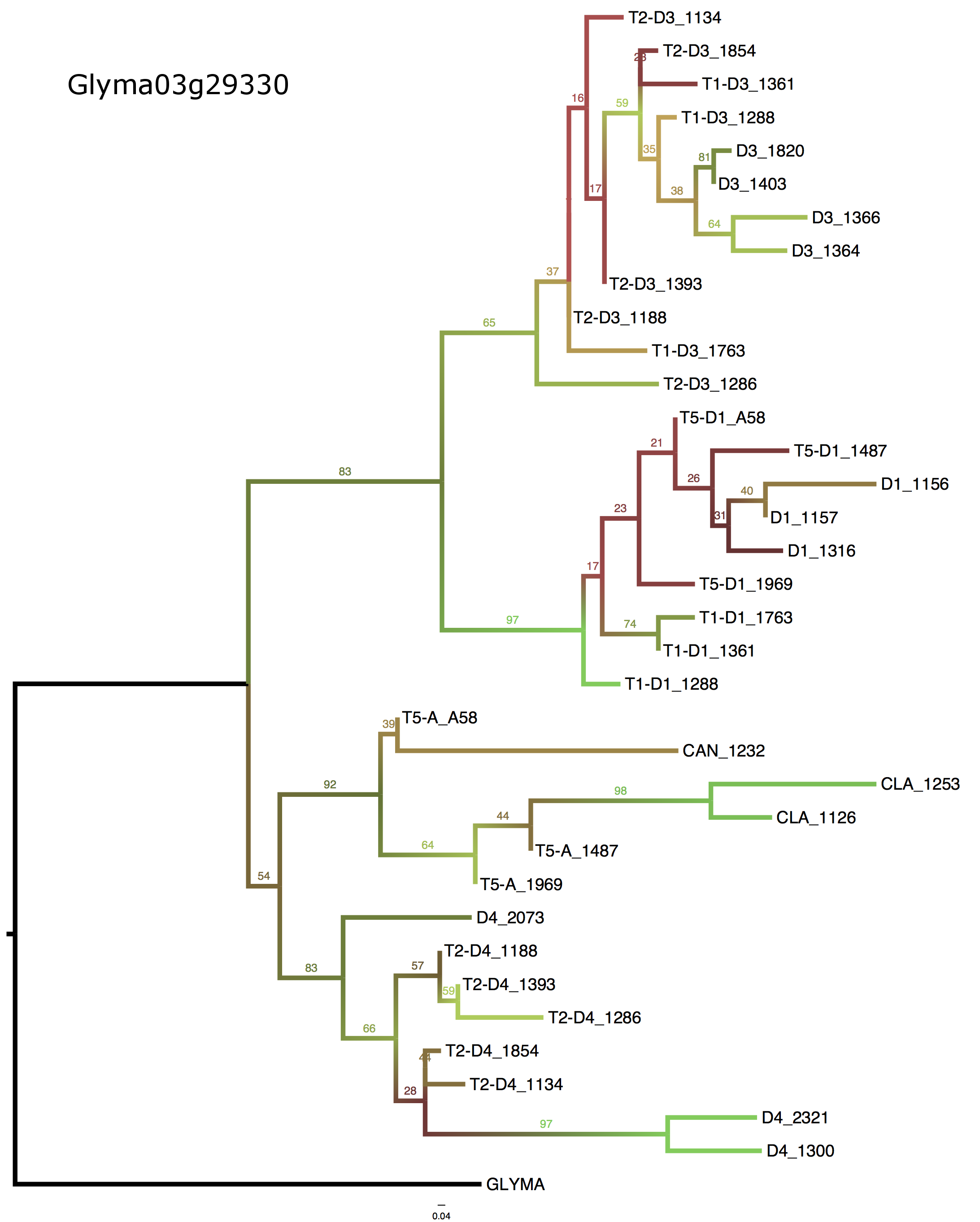

Supplement: Figures S13--S39 — MaximuThe color of the tree branch represents the bootstraping value (also with the same color). Genes are in the following order: Glyma01g35620 (Figure S13), Glyma02g11580 (Figure S14), Glyma03g29330 (Figure S15), Glyma03g36630 (Figure S16), Glyma04g39670 (Figure S17), Glyma05g05750 (Figure S18), Glyma05g09310 (Figure S19), Glyma05g26230 (Figure S20), Glyma05g37840 (Figure S21), Glyma06g18640 (Figure S22), Glyma07g03370 (Figure S23), Glyma07g17180 (Figure S24), Glyma10g42100 (Figure S25), Glyma11g13880 (Figure S26), Glyma11g33720 (Figure S27), Glyma12g04150 (Figure S28), Glyma12g12230 (Figure S29), Glyma13g17820 (Figure S30), Glyma14g03500 (Figure S31), Glyma16g00410 (Figure S32), Glyma16g01980 (Figure S33), Glyma16g04940 (Figure S34), Glyma18g04080 (Figure S35), Glyma19g03390 (Figure S36), Glyma19g32940 (Figure S37), Glyma20g24930 (Figure S38), Glyma20g32930 (Figure S39) [file peerj-02-391-s002.zip › Suplementary_Figures_S13-S39/Suplementary_Figure_15.png]

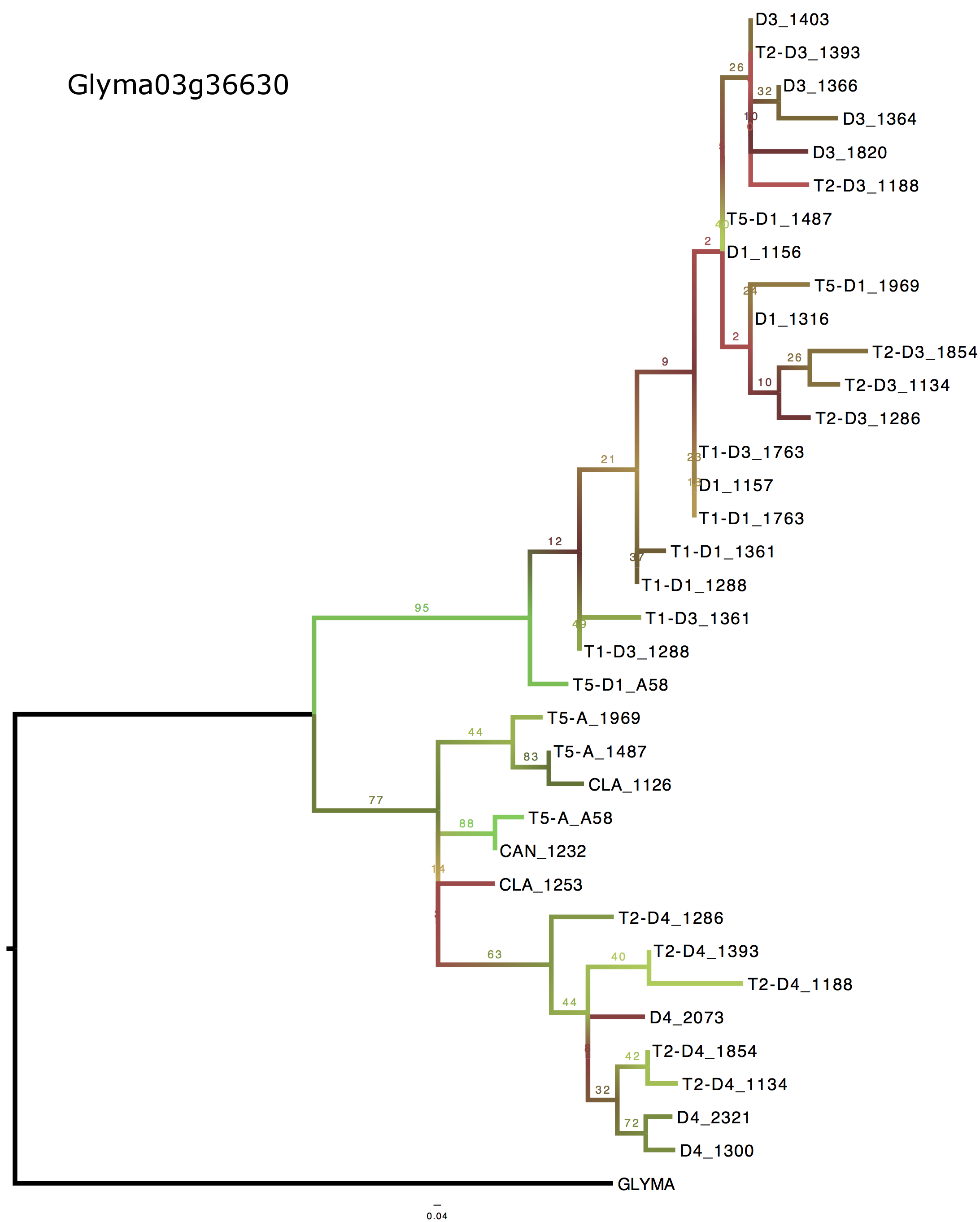

Supplement: Figures S13--S39 — MaximuThe color of the tree branch represents the bootstraping value (also with the same color). Genes are in the following order: Glyma01g35620 (Figure S13), Glyma02g11580 (Figure S14), Glyma03g29330 (Figure S15), Glyma03g36630 (Figure S16), Glyma04g39670 (Figure S17), Glyma05g05750 (Figure S18), Glyma05g09310 (Figure S19), Glyma05g26230 (Figure S20), Glyma05g37840 (Figure S21), Glyma06g18640 (Figure S22), Glyma07g03370 (Figure S23), Glyma07g17180 (Figure S24), Glyma10g42100 (Figure S25), Glyma11g13880 (Figure S26), Glyma11g33720 (Figure S27), Glyma12g04150 (Figure S28), Glyma12g12230 (Figure S29), Glyma13g17820 (Figure S30), Glyma14g03500 (Figure S31), Glyma16g00410 (Figure S32), Glyma16g01980 (Figure S33), Glyma16g04940 (Figure S34), Glyma18g04080 (Figure S35), Glyma19g03390 (Figure S36), Glyma19g32940 (Figure S37), Glyma20g24930 (Figure S38), Glyma20g32930 (Figure S39) [file peerj-02-391-s002.zip › Suplementary_Figures_S13-S39/Suplementary_Figure_16.png]

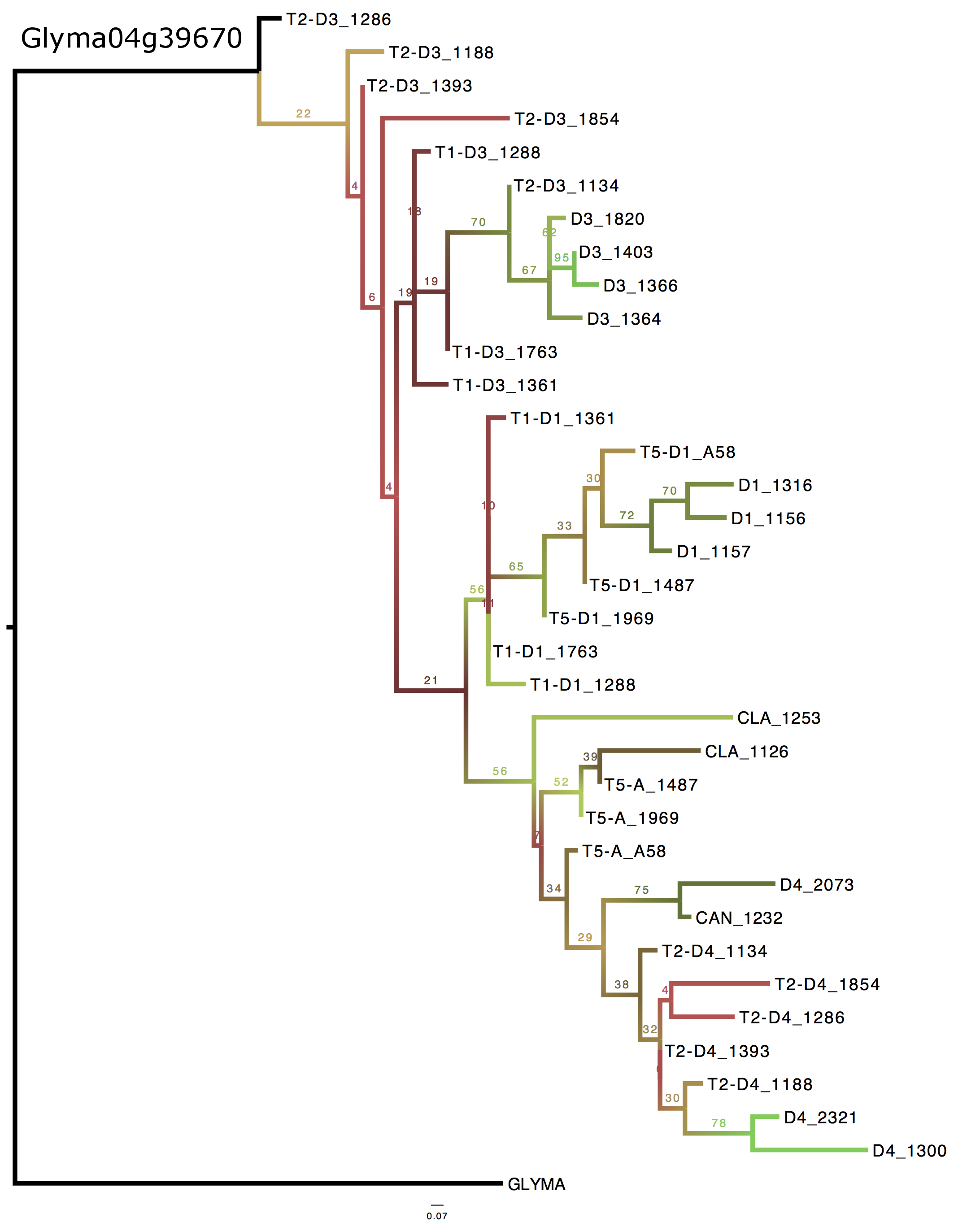

Supplement: Figures S13--S39 — MaximuThe color of the tree branch represents the bootstraping value (also with the same color). Genes are in the following order: Glyma01g35620 (Figure S13), Glyma02g11580 (Figure S14), Glyma03g29330 (Figure S15), Glyma03g36630 (Figure S16), Glyma04g39670 (Figure S17), Glyma05g05750 (Figure S18), Glyma05g09310 (Figure S19), Glyma05g26230 (Figure S20), Glyma05g37840 (Figure S21), Glyma06g18640 (Figure S22), Glyma07g03370 (Figure S23), Glyma07g17180 (Figure S24), Glyma10g42100 (Figure S25), Glyma11g13880 (Figure S26), Glyma11g33720 (Figure S27), Glyma12g04150 (Figure S28), Glyma12g12230 (Figure S29), Glyma13g17820 (Figure S30), Glyma14g03500 (Figure S31), Glyma16g00410 (Figure S32), Glyma16g01980 (Figure S33), Glyma16g04940 (Figure S34), Glyma18g04080 (Figure S35), Glyma19g03390 (Figure S36), Glyma19g32940 (Figure S37), Glyma20g24930 (Figure S38), Glyma20g32930 (Figure S39) [file peerj-02-391-s002.zip › Suplementary_Figures_S13-S39/Suplementary_Figure_17.png]

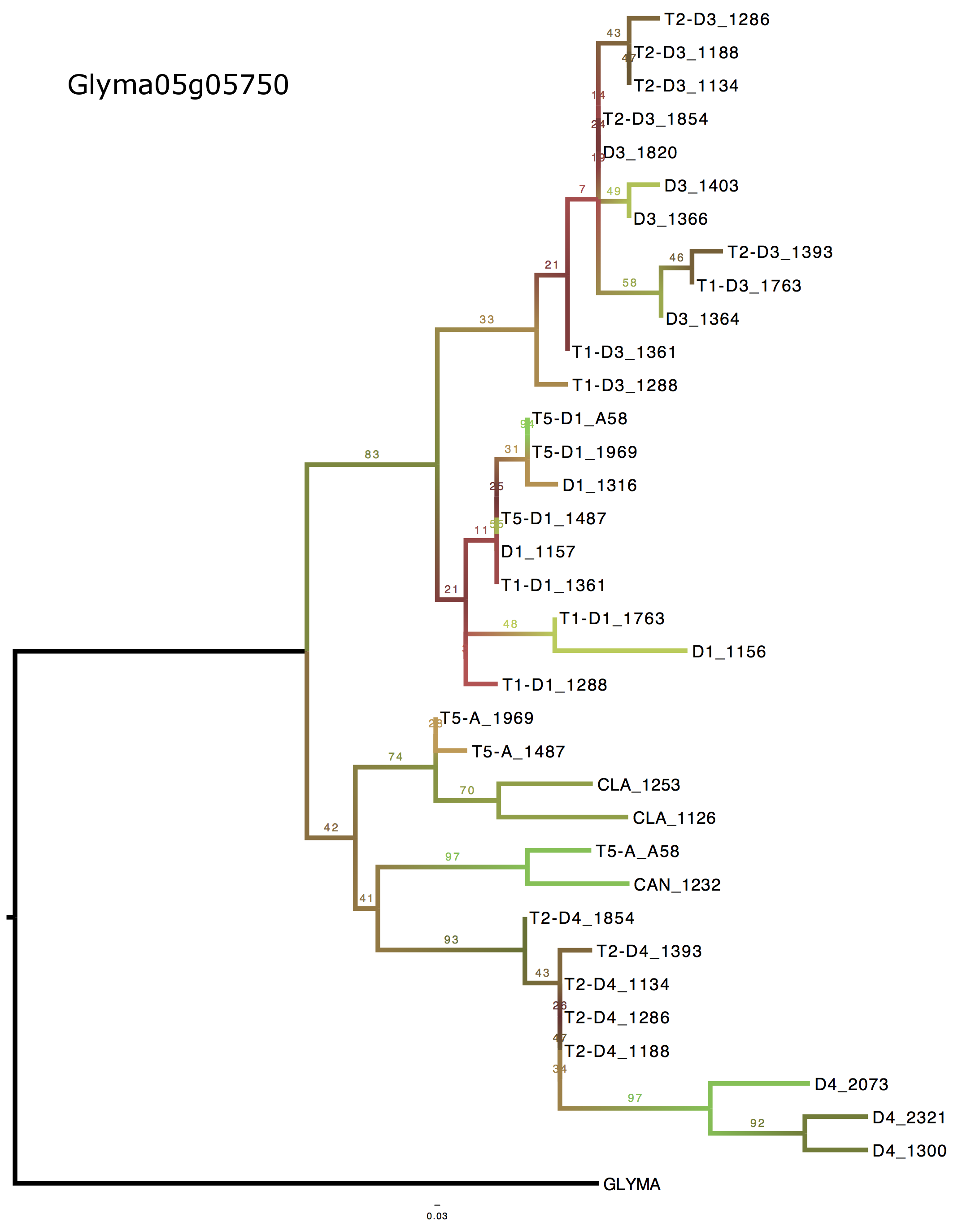

Supplement: Figures S13--S39 — MaximuThe color of the tree branch represents the bootstraping value (also with the same color). Genes are in the following order: Glyma01g35620 (Figure S13), Glyma02g11580 (Figure S14), Glyma03g29330 (Figure S15), Glyma03g36630 (Figure S16), Glyma04g39670 (Figure S17), Glyma05g05750 (Figure S18), Glyma05g09310 (Figure S19), Glyma05g26230 (Figure S20), Glyma05g37840 (Figure S21), Glyma06g18640 (Figure S22), Glyma07g03370 (Figure S23), Glyma07g17180 (Figure S24), Glyma10g42100 (Figure S25), Glyma11g13880 (Figure S26), Glyma11g33720 (Figure S27), Glyma12g04150 (Figure S28), Glyma12g12230 (Figure S29), Glyma13g17820 (Figure S30), Glyma14g03500 (Figure S31), Glyma16g00410 (Figure S32), Glyma16g01980 (Figure S33), Glyma16g04940 (Figure S34), Glyma18g04080 (Figure S35), Glyma19g03390 (Figure S36), Glyma19g32940 (Figure S37), Glyma20g24930 (Figure S38), Glyma20g32930 (Figure S39) [file peerj-02-391-s002.zip › Suplementary_Figures_S13-S39/Suplementary_Figure_18.png]

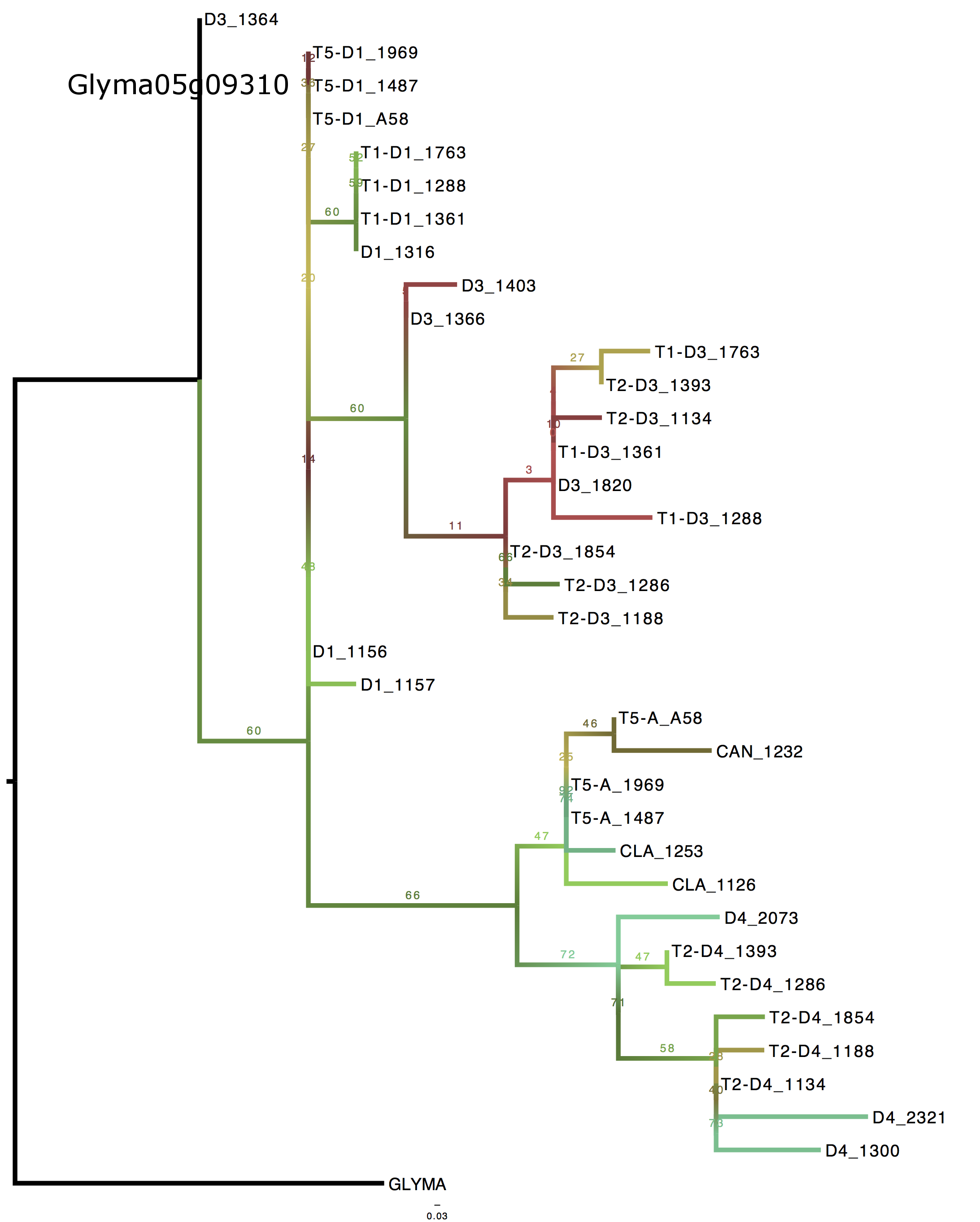

Supplement: Figures S13--S39 — MaximuThe color of the tree branch represents the bootstraping value (also with the same color). Genes are in the following order: Glyma01g35620 (Figure S13), Glyma02g11580 (Figure S14), Glyma03g29330 (Figure S15), Glyma03g36630 (Figure S16), Glyma04g39670 (Figure S17), Glyma05g05750 (Figure S18), Glyma05g09310 (Figure S19), Glyma05g26230 (Figure S20), Glyma05g37840 (Figure S21), Glyma06g18640 (Figure S22), Glyma07g03370 (Figure S23), Glyma07g17180 (Figure S24), Glyma10g42100 (Figure S25), Glyma11g13880 (Figure S26), Glyma11g33720 (Figure S27), Glyma12g04150 (Figure S28), Glyma12g12230 (Figure S29), Glyma13g17820 (Figure S30), Glyma14g03500 (Figure S31), Glyma16g00410 (Figure S32), Glyma16g01980 (Figure S33), Glyma16g04940 (Figure S34), Glyma18g04080 (Figure S35), Glyma19g03390 (Figure S36), Glyma19g32940 (Figure S37), Glyma20g24930 (Figure S38), Glyma20g32930 (Figure S39) [file peerj-02-391-s002.zip › Suplementary_Figures_S13-S39/Suplementary_Figure_19.png]

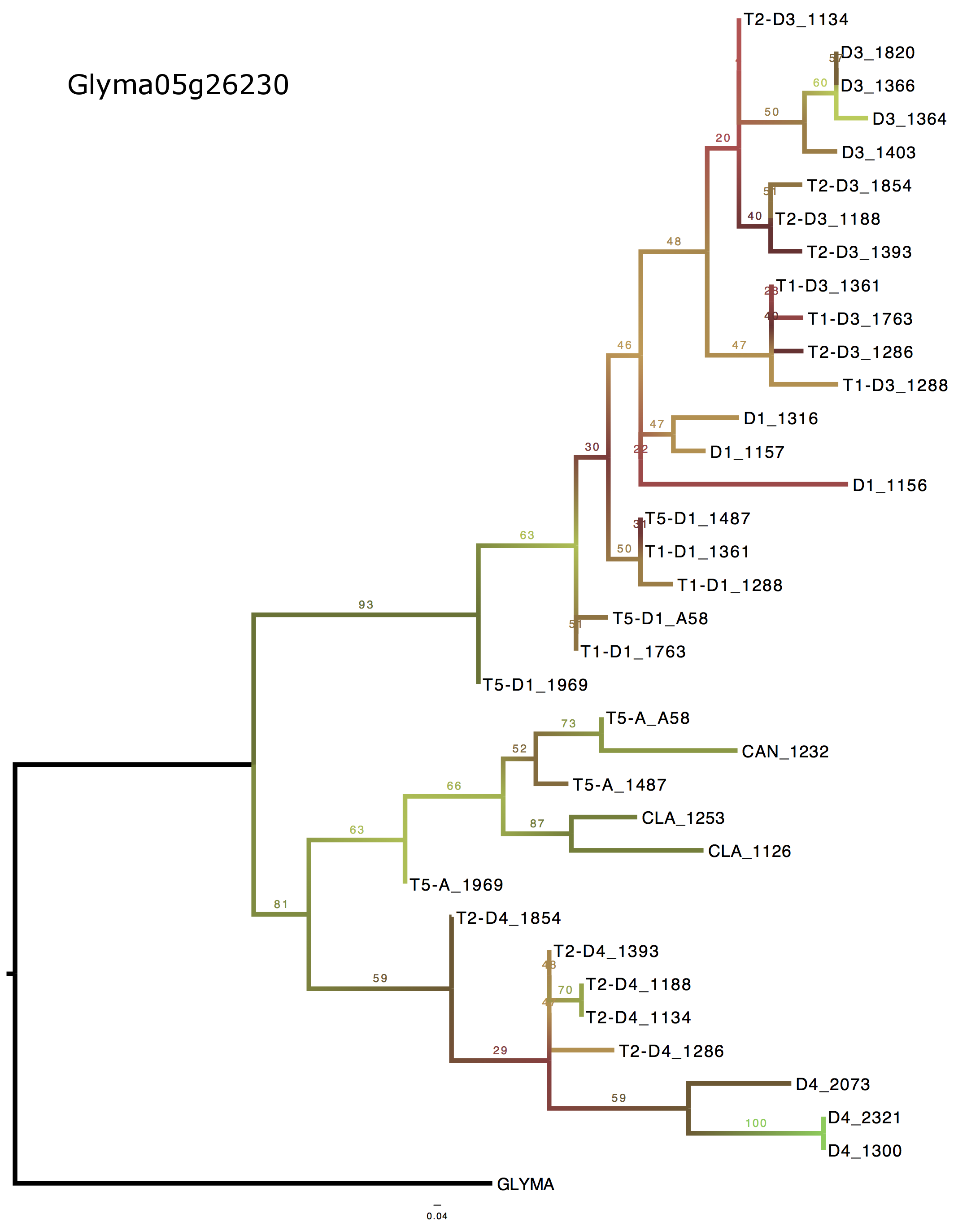

Supplement: Figures S13--S39 — MaximuThe color of the tree branch represents the bootstraping value (also with the same color). Genes are in the following order: Glyma01g35620 (Figure S13), Glyma02g11580 (Figure S14), Glyma03g29330 (Figure S15), Glyma03g36630 (Figure S16), Glyma04g39670 (Figure S17), Glyma05g05750 (Figure S18), Glyma05g09310 (Figure S19), Glyma05g26230 (Figure S20), Glyma05g37840 (Figure S21), Glyma06g18640 (Figure S22), Glyma07g03370 (Figure S23), Glyma07g17180 (Figure S24), Glyma10g42100 (Figure S25), Glyma11g13880 (Figure S26), Glyma11g33720 (Figure S27), Glyma12g04150 (Figure S28), Glyma12g12230 (Figure S29), Glyma13g17820 (Figure S30), Glyma14g03500 (Figure S31), Glyma16g00410 (Figure S32), Glyma16g01980 (Figure S33), Glyma16g04940 (Figure S34), Glyma18g04080 (Figure S35), Glyma19g03390 (Figure S36), Glyma19g32940 (Figure S37), Glyma20g24930 (Figure S38), Glyma20g32930 (Figure S39) [file peerj-02-391-s002.zip › Suplementary_Figures_S13-S39/Suplementary_Figure_20.png]

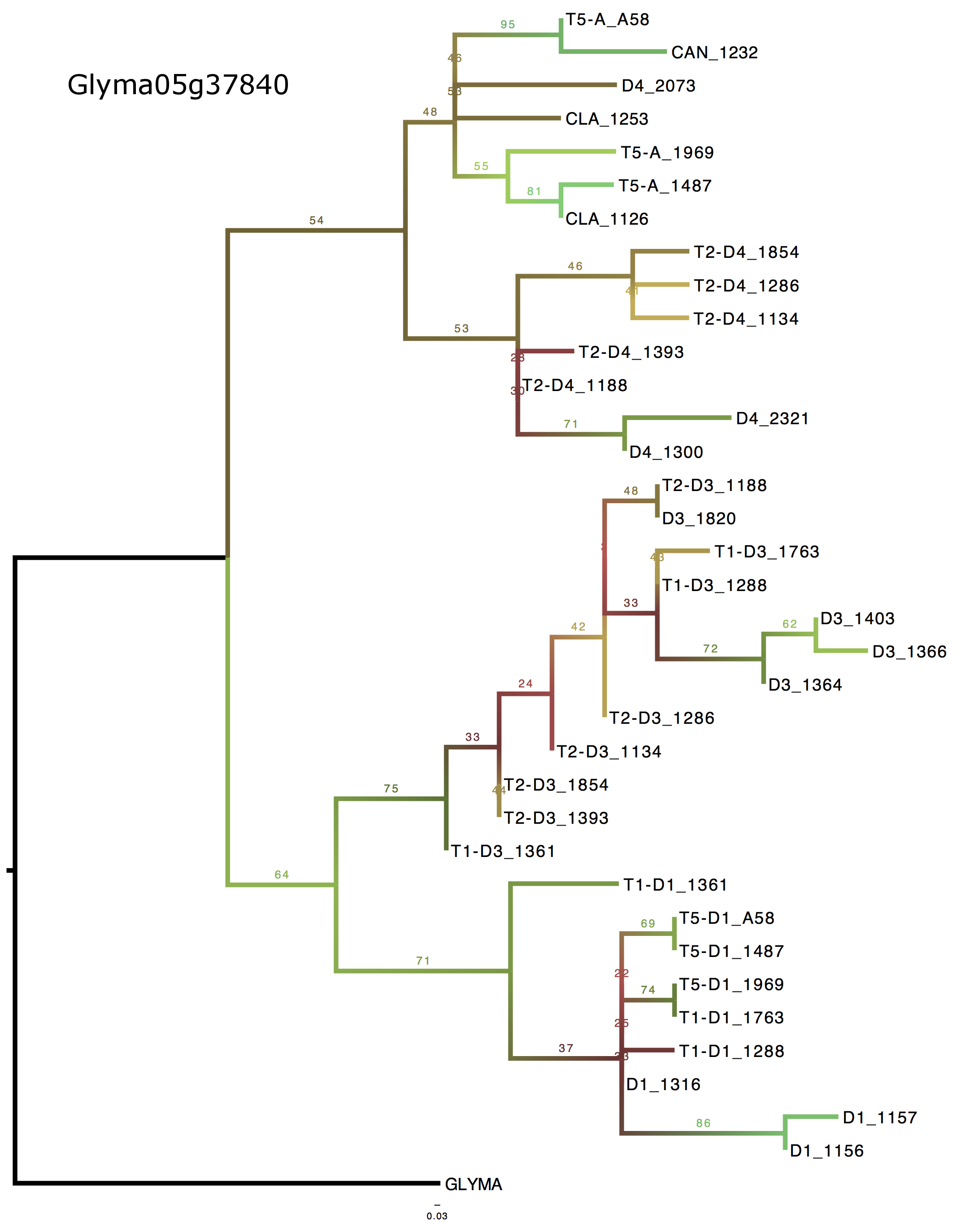

Supplement: Figures S13--S39 — MaximuThe color of the tree branch represents the bootstraping value (also with the same color). Genes are in the following order: Glyma01g35620 (Figure S13), Glyma02g11580 (Figure S14), Glyma03g29330 (Figure S15), Glyma03g36630 (Figure S16), Glyma04g39670 (Figure S17), Glyma05g05750 (Figure S18), Glyma05g09310 (Figure S19), Glyma05g26230 (Figure S20), Glyma05g37840 (Figure S21), Glyma06g18640 (Figure S22), Glyma07g03370 (Figure S23), Glyma07g17180 (Figure S24), Glyma10g42100 (Figure S25), Glyma11g13880 (Figure S26), Glyma11g33720 (Figure S27), Glyma12g04150 (Figure S28), Glyma12g12230 (Figure S29), Glyma13g17820 (Figure S30), Glyma14g03500 (Figure S31), Glyma16g00410 (Figure S32), Glyma16g01980 (Figure S33), Glyma16g04940 (Figure S34), Glyma18g04080 (Figure S35), Glyma19g03390 (Figure S36), Glyma19g32940 (Figure S37), Glyma20g24930 (Figure S38), Glyma20g32930 (Figure S39) [file peerj-02-391-s002.zip › Suplementary_Figures_S13-S39/Suplementary_Figure_21.png]

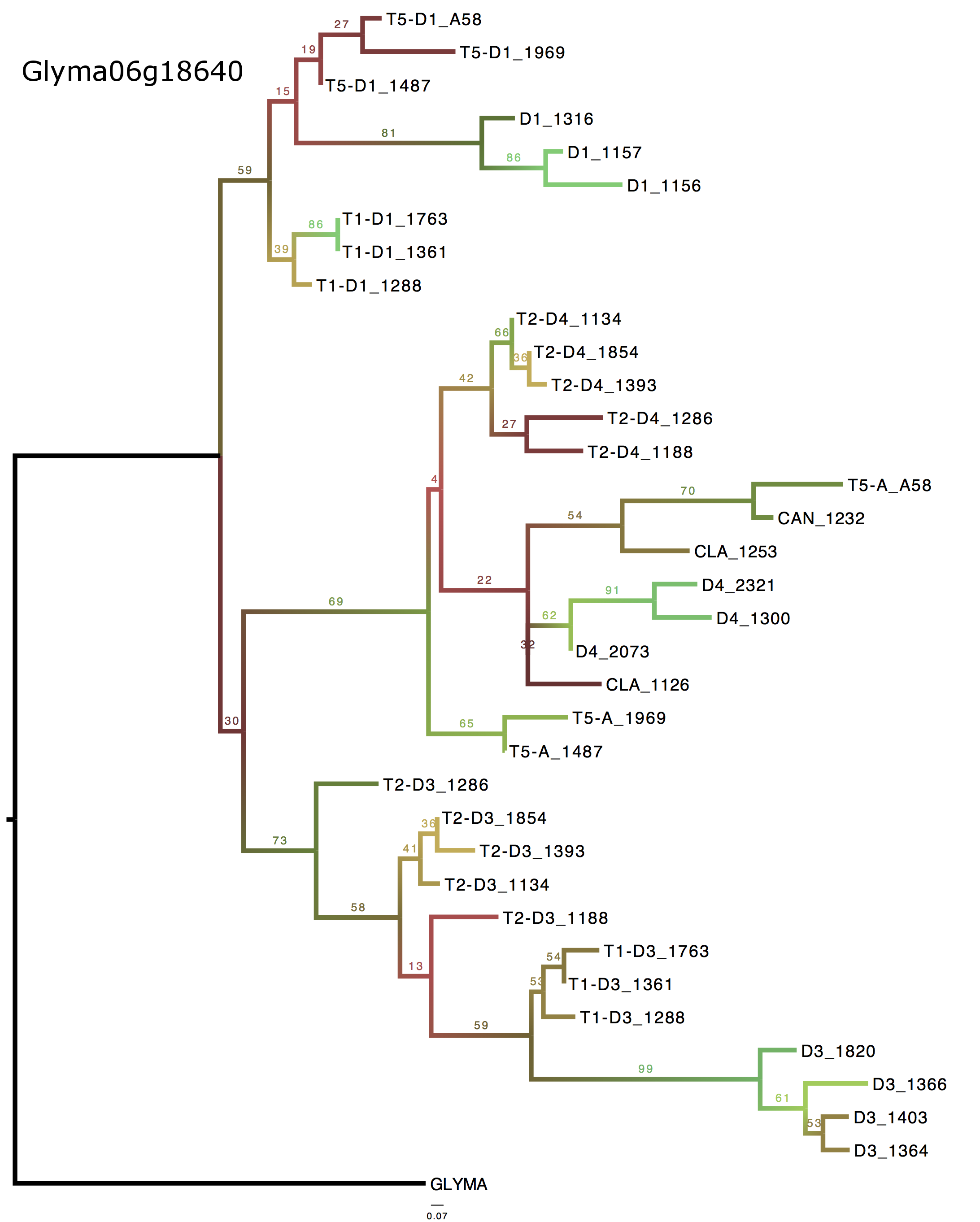

Supplement: Figures S13--S39 — MaximuThe color of the tree branch represents the bootstraping value (also with the same color). Genes are in the following order: Glyma01g35620 (Figure S13), Glyma02g11580 (Figure S14), Glyma03g29330 (Figure S15), Glyma03g36630 (Figure S16), Glyma04g39670 (Figure S17), Glyma05g05750 (Figure S18), Glyma05g09310 (Figure S19), Glyma05g26230 (Figure S20), Glyma05g37840 (Figure S21), Glyma06g18640 (Figure S22), Glyma07g03370 (Figure S23), Glyma07g17180 (Figure S24), Glyma10g42100 (Figure S25), Glyma11g13880 (Figure S26), Glyma11g33720 (Figure S27), Glyma12g04150 (Figure S28), Glyma12g12230 (Figure S29), Glyma13g17820 (Figure S30), Glyma14g03500 (Figure S31), Glyma16g00410 (Figure S32), Glyma16g01980 (Figure S33), Glyma16g04940 (Figure S34), Glyma18g04080 (Figure S35), Glyma19g03390 (Figure S36), Glyma19g32940 (Figure S37), Glyma20g24930 (Figure S38), Glyma20g32930 (Figure S39) [file peerj-02-391-s002.zip › Suplementary_Figures_S13-S39/Suplementary_Figure_22.png]

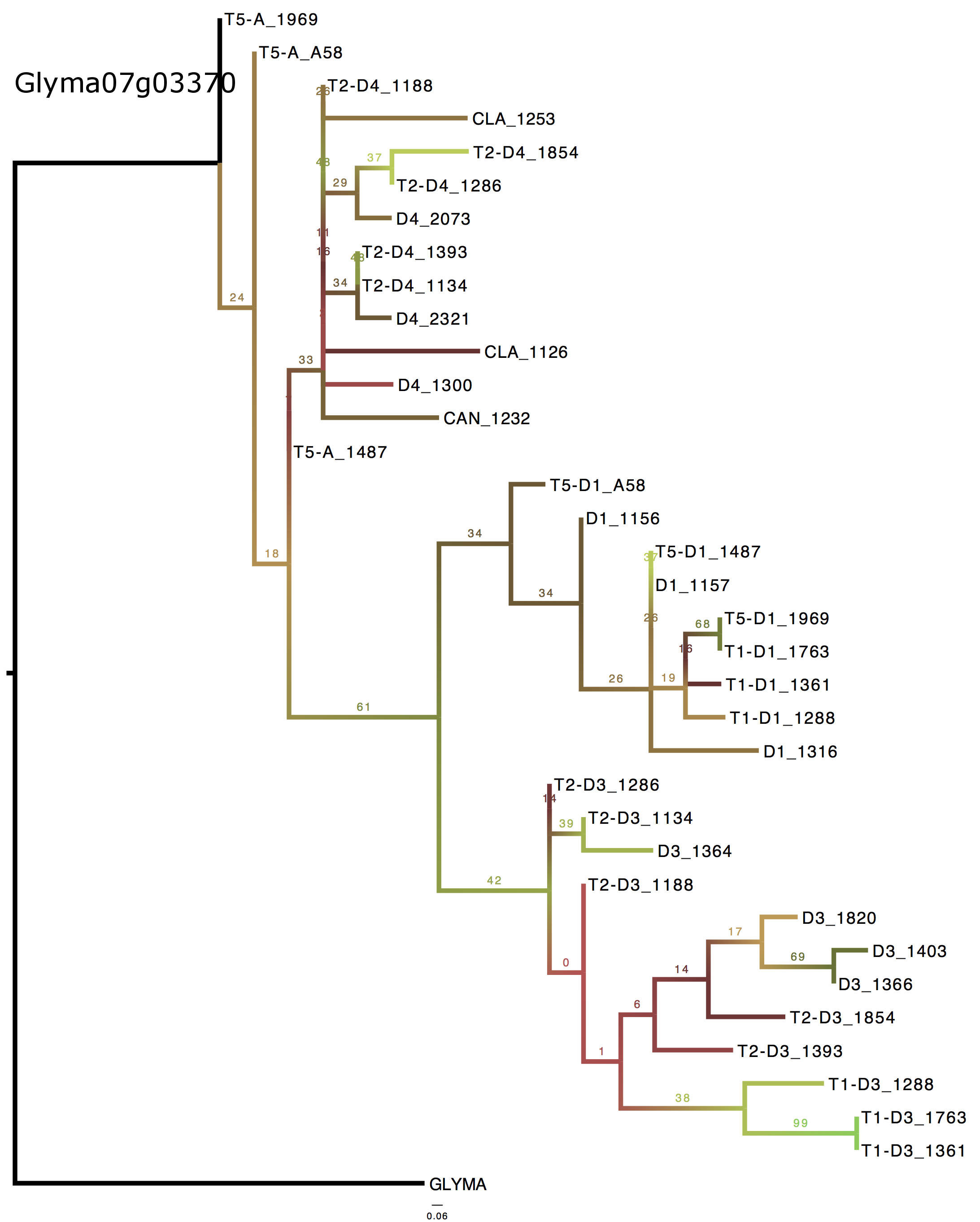

Supplement: Figures S13--S39 — MaximuThe color of the tree branch represents the bootstraping value (also with the same color). Genes are in the following order: Glyma01g35620 (Figure S13), Glyma02g11580 (Figure S14), Glyma03g29330 (Figure S15), Glyma03g36630 (Figure S16), Glyma04g39670 (Figure S17), Glyma05g05750 (Figure S18), Glyma05g09310 (Figure S19), Glyma05g26230 (Figure S20), Glyma05g37840 (Figure S21), Glyma06g18640 (Figure S22), Glyma07g03370 (Figure S23), Glyma07g17180 (Figure S24), Glyma10g42100 (Figure S25), Glyma11g13880 (Figure S26), Glyma11g33720 (Figure S27), Glyma12g04150 (Figure S28), Glyma12g12230 (Figure S29), Glyma13g17820 (Figure S30), Glyma14g03500 (Figure S31), Glyma16g00410 (Figure S32), Glyma16g01980 (Figure S33), Glyma16g04940 (Figure S34), Glyma18g04080 (Figure S35), Glyma19g03390 (Figure S36), Glyma19g32940 (Figure S37), Glyma20g24930 (Figure S38), Glyma20g32930 (Figure S39) [file peerj-02-391-s002.zip › Suplementary_Figures_S13-S39/Suplementary_Figure_23.png]

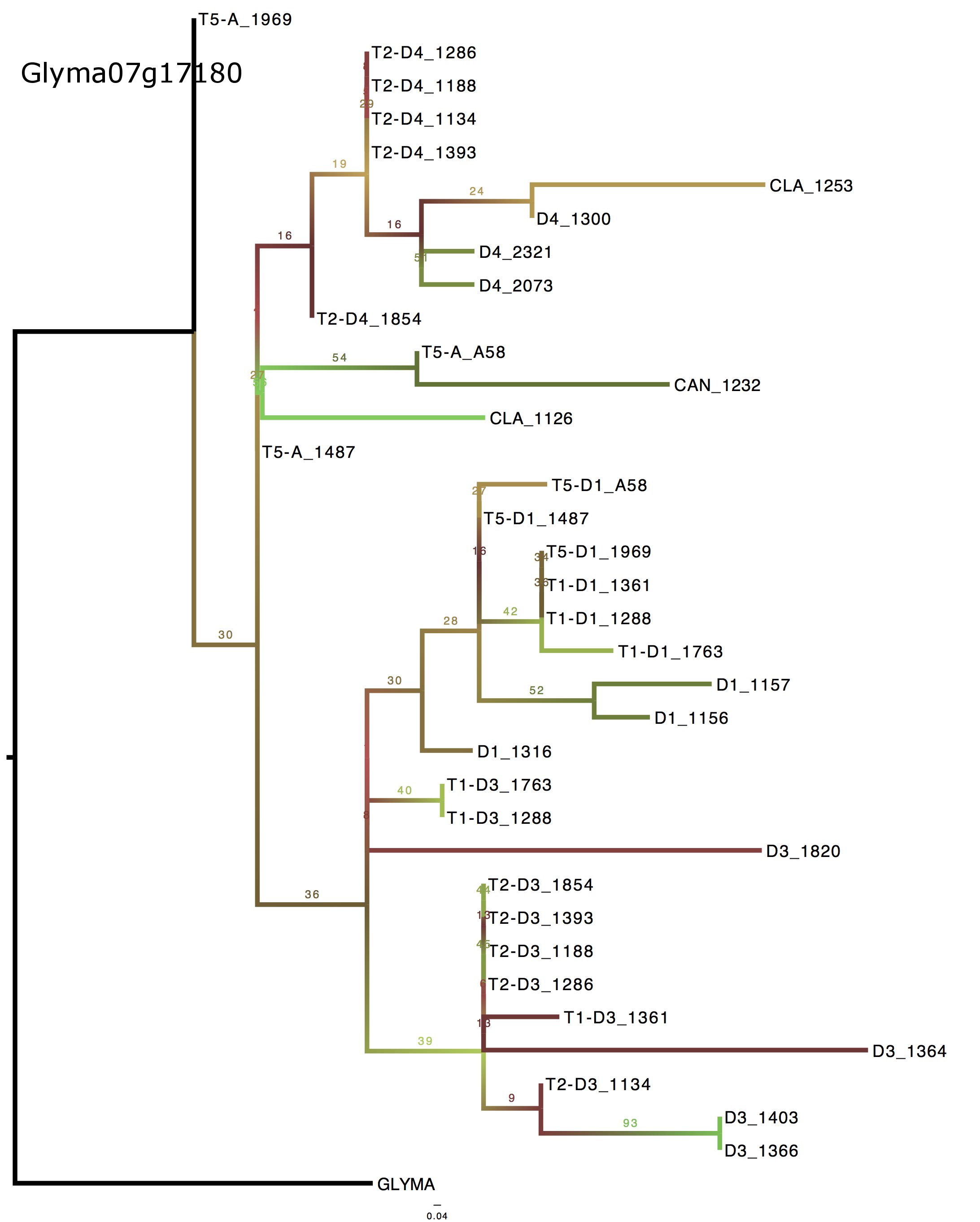

Supplement: Figures S13--S39 — MaximuThe color of the tree branch represents the bootstraping value (also with the same color). Genes are in the following order: Glyma01g35620 (Figure S13), Glyma02g11580 (Figure S14), Glyma03g29330 (Figure S15), Glyma03g36630 (Figure S16), Glyma04g39670 (Figure S17), Glyma05g05750 (Figure S18), Glyma05g09310 (Figure S19), Glyma05g26230 (Figure S20), Glyma05g37840 (Figure S21), Glyma06g18640 (Figure S22), Glyma07g03370 (Figure S23), Glyma07g17180 (Figure S24), Glyma10g42100 (Figure S25), Glyma11g13880 (Figure S26), Glyma11g33720 (Figure S27), Glyma12g04150 (Figure S28), Glyma12g12230 (Figure S29), Glyma13g17820 (Figure S30), Glyma14g03500 (Figure S31), Glyma16g00410 (Figure S32), Glyma16g01980 (Figure S33), Glyma16g04940 (Figure S34), Glyma18g04080 (Figure S35), Glyma19g03390 (Figure S36), Glyma19g32940 (Figure S37), Glyma20g24930 (Figure S38), Glyma20g32930 (Figure S39) [file peerj-02-391-s002.zip › Suplementary_Figures_S13-S39/Suplementary_Figure_24.png]

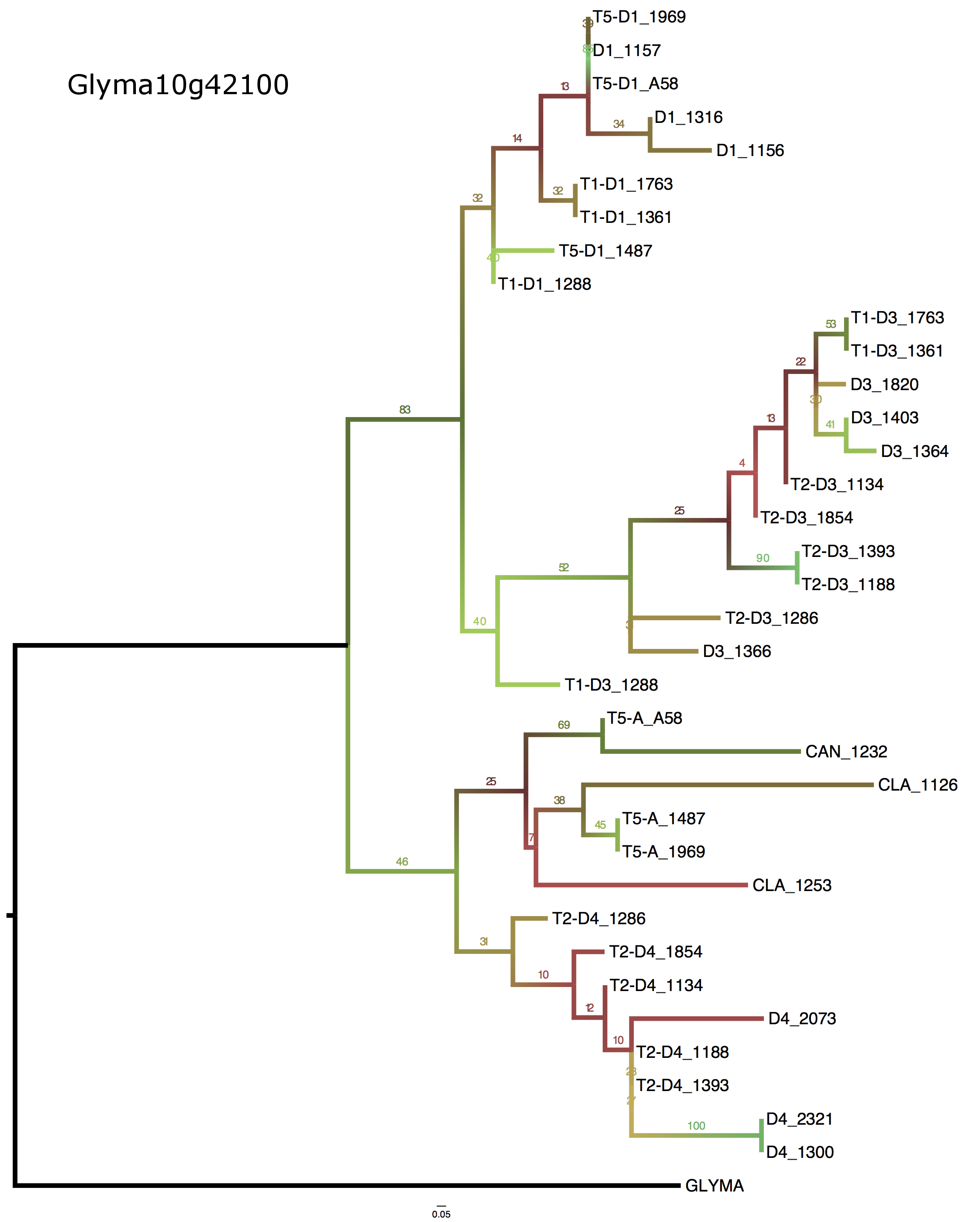

Supplement: Figures S13--S39 — MaximuThe color of the tree branch represents the bootstraping value (also with the same color). Genes are in the following order: Glyma01g35620 (Figure S13), Glyma02g11580 (Figure S14), Glyma03g29330 (Figure S15), Glyma03g36630 (Figure S16), Glyma04g39670 (Figure S17), Glyma05g05750 (Figure S18), Glyma05g09310 (Figure S19), Glyma05g26230 (Figure S20), Glyma05g37840 (Figure S21), Glyma06g18640 (Figure S22), Glyma07g03370 (Figure S23), Glyma07g17180 (Figure S24), Glyma10g42100 (Figure S25), Glyma11g13880 (Figure S26), Glyma11g33720 (Figure S27), Glyma12g04150 (Figure S28), Glyma12g12230 (Figure S29), Glyma13g17820 (Figure S30), Glyma14g03500 (Figure S31), Glyma16g00410 (Figure S32), Glyma16g01980 (Figure S33), Glyma16g04940 (Figure S34), Glyma18g04080 (Figure S35), Glyma19g03390 (Figure S36), Glyma19g32940 (Figure S37), Glyma20g24930 (Figure S38), Glyma20g32930 (Figure S39) [file peerj-02-391-s002.zip › Suplementary_Figures_S13-S39/Suplementary_Figure_25.png]

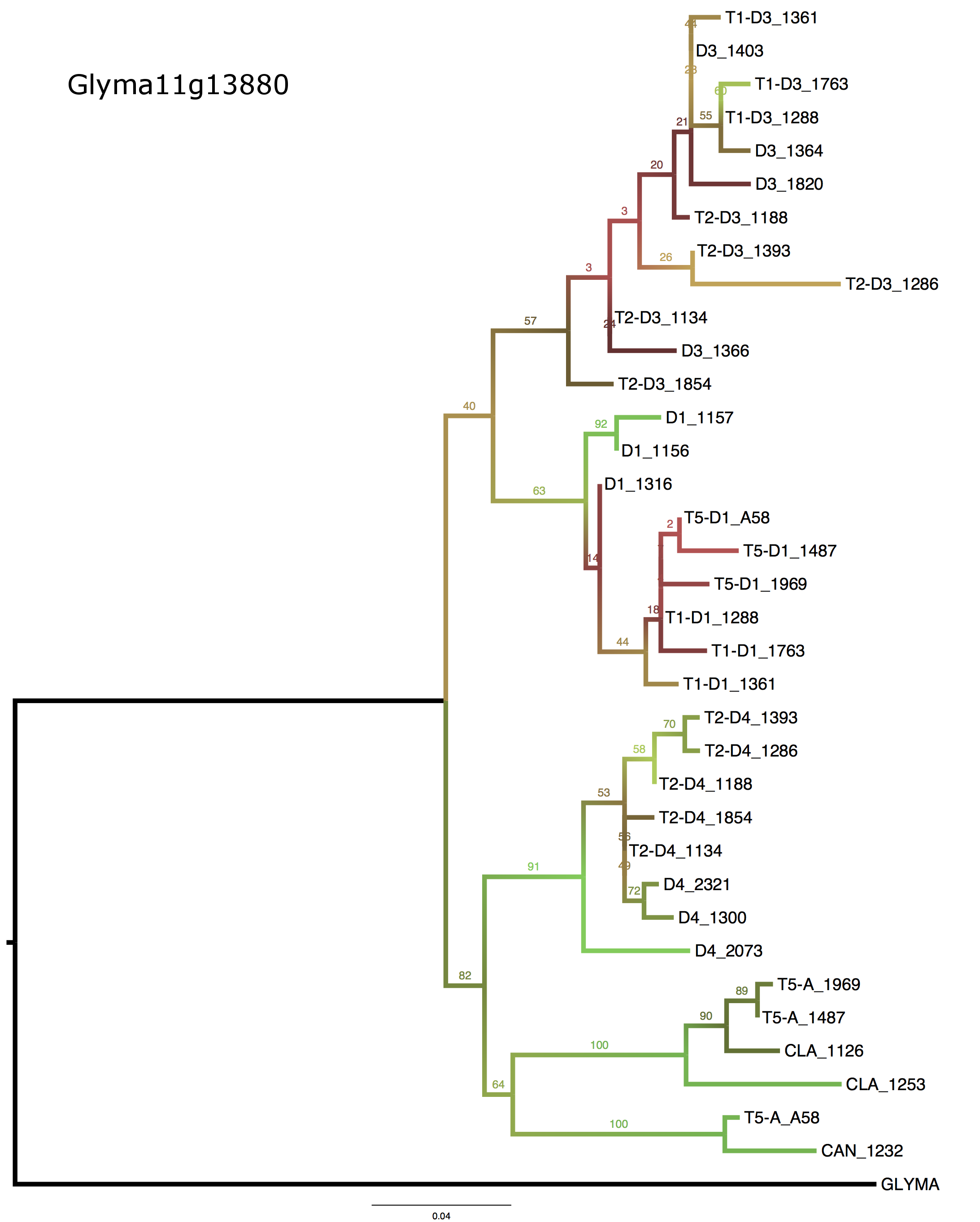

Supplement: Figures S13--S39 — MaximuThe color of the tree branch represents the bootstraping value (also with the same color). Genes are in the following order: Glyma01g35620 (Figure S13), Glyma02g11580 (Figure S14), Glyma03g29330 (Figure S15), Glyma03g36630 (Figure S16), Glyma04g39670 (Figure S17), Glyma05g05750 (Figure S18), Glyma05g09310 (Figure S19), Glyma05g26230 (Figure S20), Glyma05g37840 (Figure S21), Glyma06g18640 (Figure S22), Glyma07g03370 (Figure S23), Glyma07g17180 (Figure S24), Glyma10g42100 (Figure S25), Glyma11g13880 (Figure S26), Glyma11g33720 (Figure S27), Glyma12g04150 (Figure S28), Glyma12g12230 (Figure S29), Glyma13g17820 (Figure S30), Glyma14g03500 (Figure S31), Glyma16g00410 (Figure S32), Glyma16g01980 (Figure S33), Glyma16g04940 (Figure S34), Glyma18g04080 (Figure S35), Glyma19g03390 (Figure S36), Glyma19g32940 (Figure S37), Glyma20g24930 (Figure S38), Glyma20g32930 (Figure S39) [file peerj-02-391-s002.zip › Suplementary_Figures_S13-S39/Suplementary_Figure_26.png]

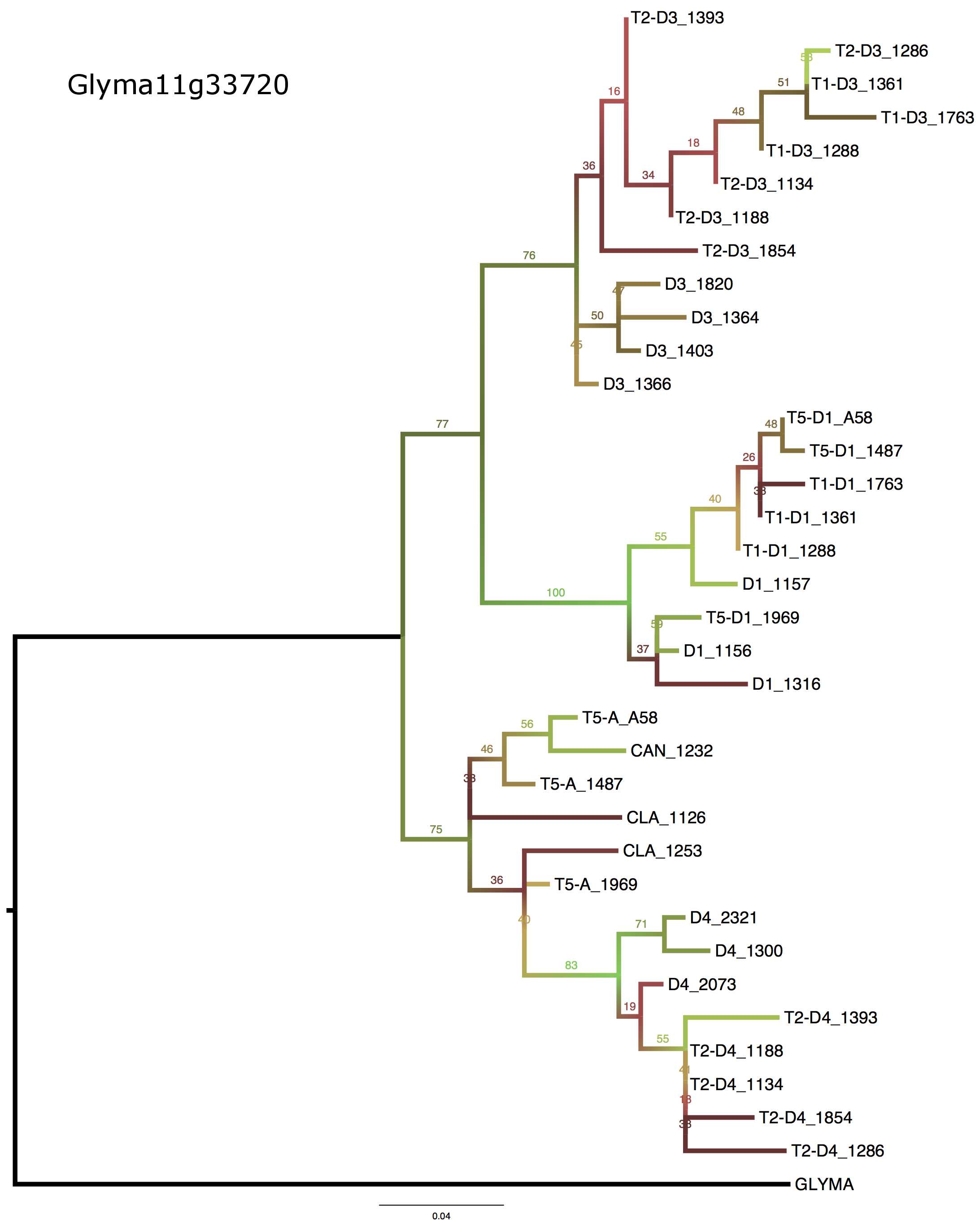

Supplement: Figures S13--S39 — MaximuThe color of the tree branch represents the bootstraping value (also with the same color). Genes are in the following order: Glyma01g35620 (Figure S13), Glyma02g11580 (Figure S14), Glyma03g29330 (Figure S15), Glyma03g36630 (Figure S16), Glyma04g39670 (Figure S17), Glyma05g05750 (Figure S18), Glyma05g09310 (Figure S19), Glyma05g26230 (Figure S20), Glyma05g37840 (Figure S21), Glyma06g18640 (Figure S22), Glyma07g03370 (Figure S23), Glyma07g17180 (Figure S24), Glyma10g42100 (Figure S25), Glyma11g13880 (Figure S26), Glyma11g33720 (Figure S27), Glyma12g04150 (Figure S28), Glyma12g12230 (Figure S29), Glyma13g17820 (Figure S30), Glyma14g03500 (Figure S31), Glyma16g00410 (Figure S32), Glyma16g01980 (Figure S33), Glyma16g04940 (Figure S34), Glyma18g04080 (Figure S35), Glyma19g03390 (Figure S36), Glyma19g32940 (Figure S37), Glyma20g24930 (Figure S38), Glyma20g32930 (Figure S39) [file peerj-02-391-s002.zip › Suplementary_Figures_S13-S39/Suplementary_Figure_27.png]

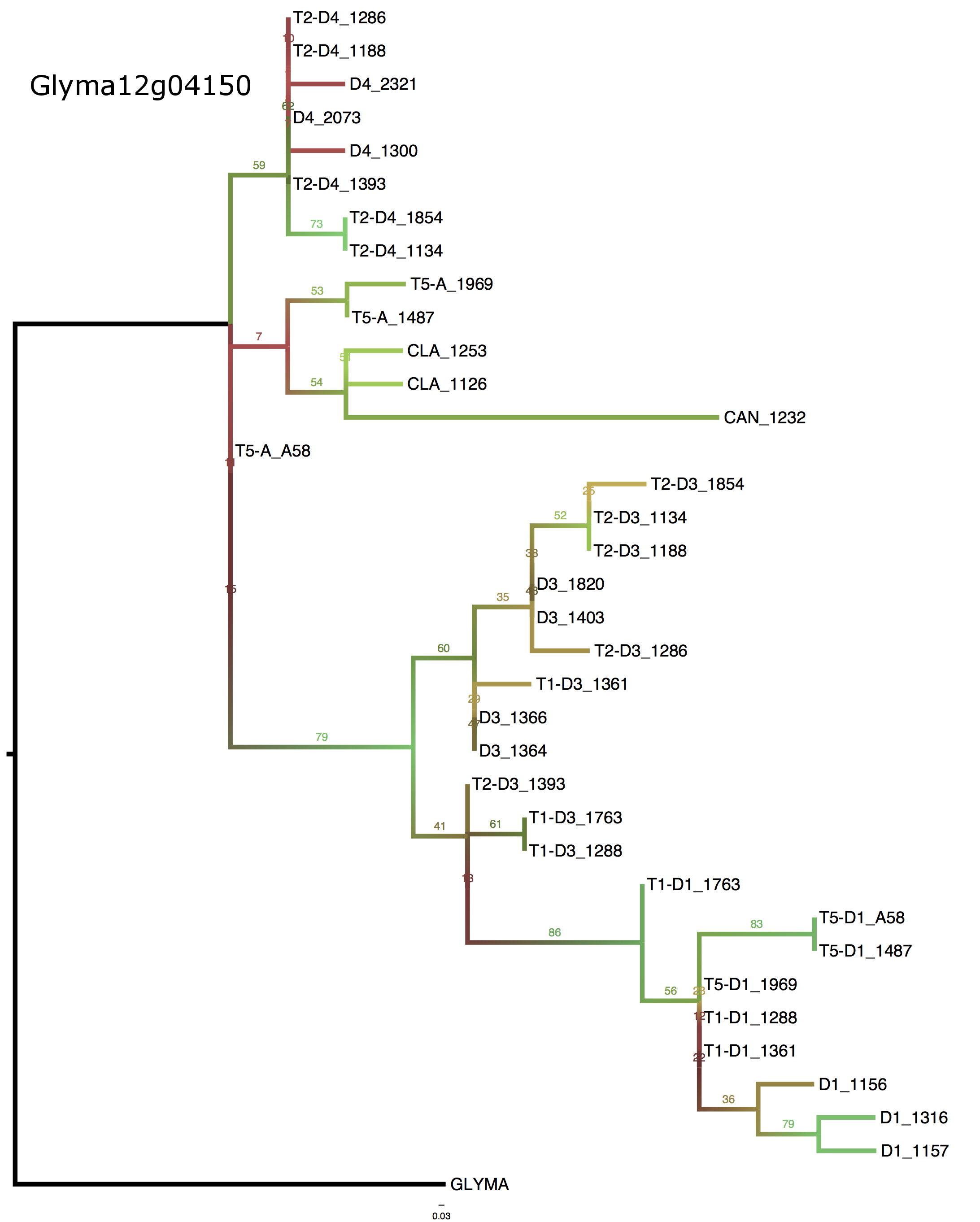

Supplement: Figures S13--S39 — MaximuThe color of the tree branch represents the bootstraping value (also with the same color). Genes are in the following order: Glyma01g35620 (Figure S13), Glyma02g11580 (Figure S14), Glyma03g29330 (Figure S15), Glyma03g36630 (Figure S16), Glyma04g39670 (Figure S17), Glyma05g05750 (Figure S18), Glyma05g09310 (Figure S19), Glyma05g26230 (Figure S20), Glyma05g37840 (Figure S21), Glyma06g18640 (Figure S22), Glyma07g03370 (Figure S23), Glyma07g17180 (Figure S24), Glyma10g42100 (Figure S25), Glyma11g13880 (Figure S26), Glyma11g33720 (Figure S27), Glyma12g04150 (Figure S28), Glyma12g12230 (Figure S29), Glyma13g17820 (Figure S30), Glyma14g03500 (Figure S31), Glyma16g00410 (Figure S32), Glyma16g01980 (Figure S33), Glyma16g04940 (Figure S34), Glyma18g04080 (Figure S35), Glyma19g03390 (Figure S36), Glyma19g32940 (Figure S37), Glyma20g24930 (Figure S38), Glyma20g32930 (Figure S39) [file peerj-02-391-s002.zip › Suplementary_Figures_S13-S39/Suplementary_Figure_28.png]

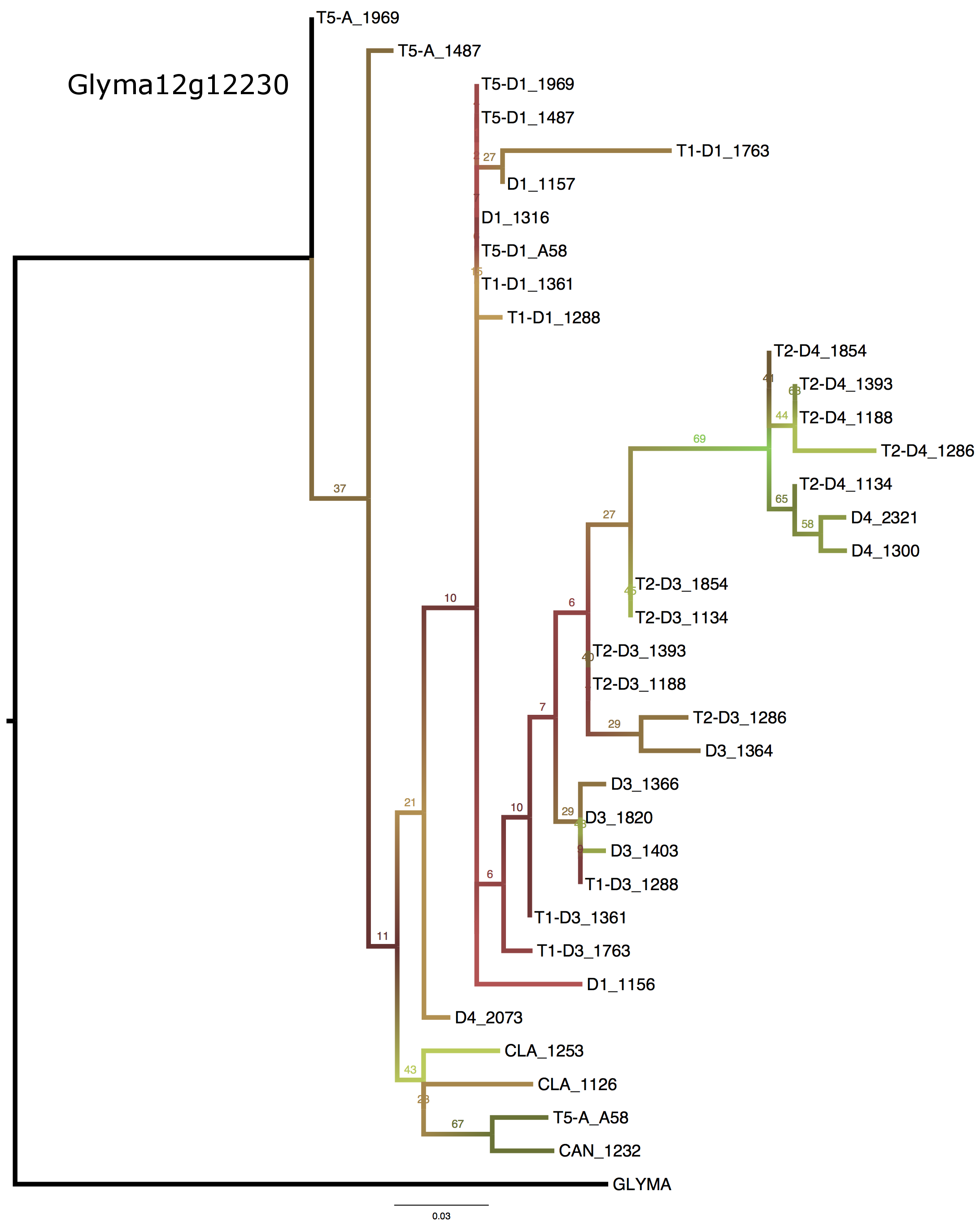

Supplement: Figures S13--S39 — MaximuThe color of the tree branch represents the bootstraping value (also with the same color). Genes are in the following order: Glyma01g35620 (Figure S13), Glyma02g11580 (Figure S14), Glyma03g29330 (Figure S15), Glyma03g36630 (Figure S16), Glyma04g39670 (Figure S17), Glyma05g05750 (Figure S18), Glyma05g09310 (Figure S19), Glyma05g26230 (Figure S20), Glyma05g37840 (Figure S21), Glyma06g18640 (Figure S22), Glyma07g03370 (Figure S23), Glyma07g17180 (Figure S24), Glyma10g42100 (Figure S25), Glyma11g13880 (Figure S26), Glyma11g33720 (Figure S27), Glyma12g04150 (Figure S28), Glyma12g12230 (Figure S29), Glyma13g17820 (Figure S30), Glyma14g03500 (Figure S31), Glyma16g00410 (Figure S32), Glyma16g01980 (Figure S33), Glyma16g04940 (Figure S34), Glyma18g04080 (Figure S35), Glyma19g03390 (Figure S36), Glyma19g32940 (Figure S37), Glyma20g24930 (Figure S38), Glyma20g32930 (Figure S39) [file peerj-02-391-s002.zip › Suplementary_Figures_S13-S39/Suplementary_Figure_29.png]

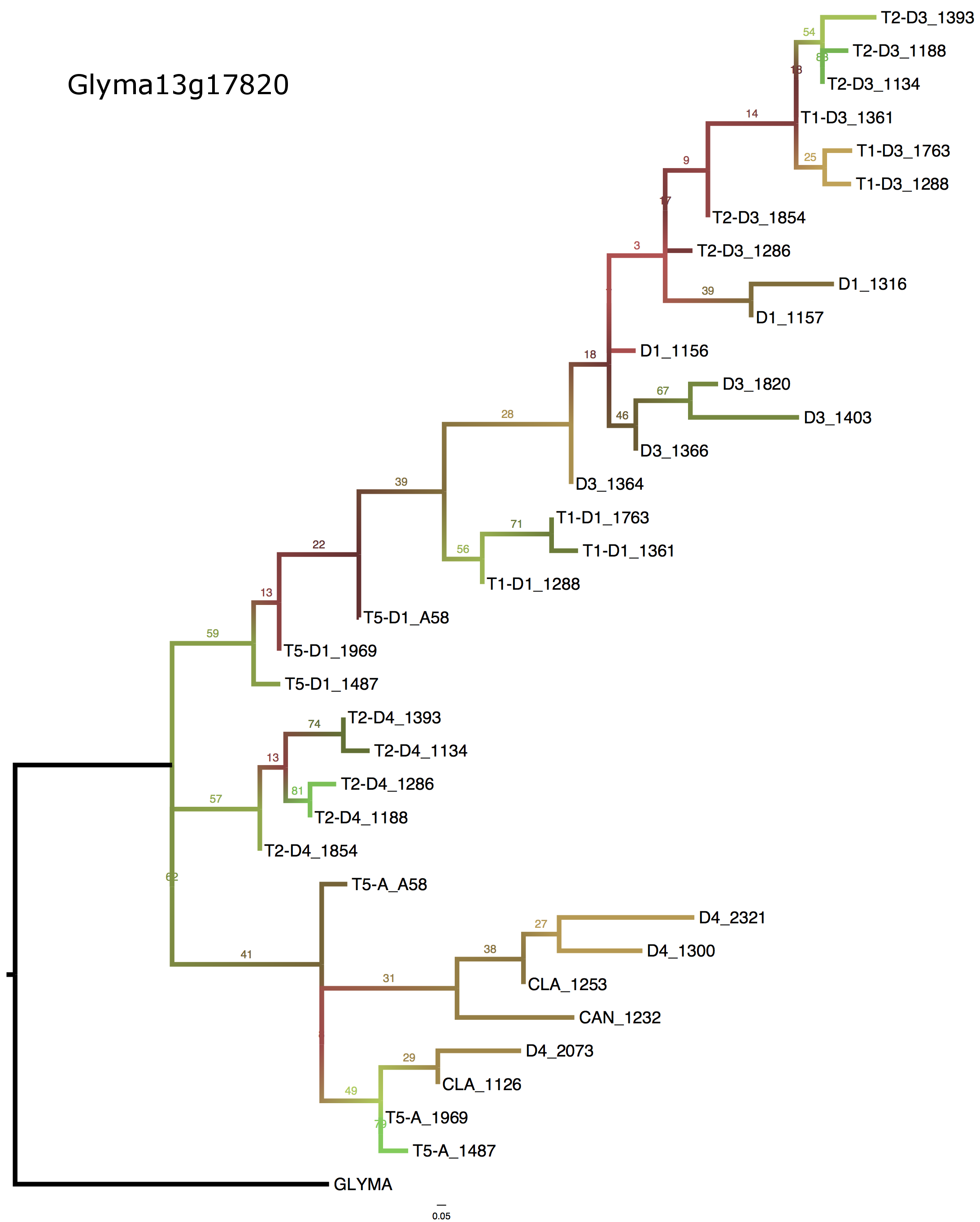

Supplement: Figures S13--S39 — MaximuThe color of the tree branch represents the bootstraping value (also with the same color). Genes are in the following order: Glyma01g35620 (Figure S13), Glyma02g11580 (Figure S14), Glyma03g29330 (Figure S15), Glyma03g36630 (Figure S16), Glyma04g39670 (Figure S17), Glyma05g05750 (Figure S18), Glyma05g09310 (Figure S19), Glyma05g26230 (Figure S20), Glyma05g37840 (Figure S21), Glyma06g18640 (Figure S22), Glyma07g03370 (Figure S23), Glyma07g17180 (Figure S24), Glyma10g42100 (Figure S25), Glyma11g13880 (Figure S26), Glyma11g33720 (Figure S27), Glyma12g04150 (Figure S28), Glyma12g12230 (Figure S29), Glyma13g17820 (Figure S30), Glyma14g03500 (Figure S31), Glyma16g00410 (Figure S32), Glyma16g01980 (Figure S33), Glyma16g04940 (Figure S34), Glyma18g04080 (Figure S35), Glyma19g03390 (Figure S36), Glyma19g32940 (Figure S37), Glyma20g24930 (Figure S38), Glyma20g32930 (Figure S39) [file peerj-02-391-s002.zip › Suplementary_Figures_S13-S39/Suplementary_Figure_30.png]

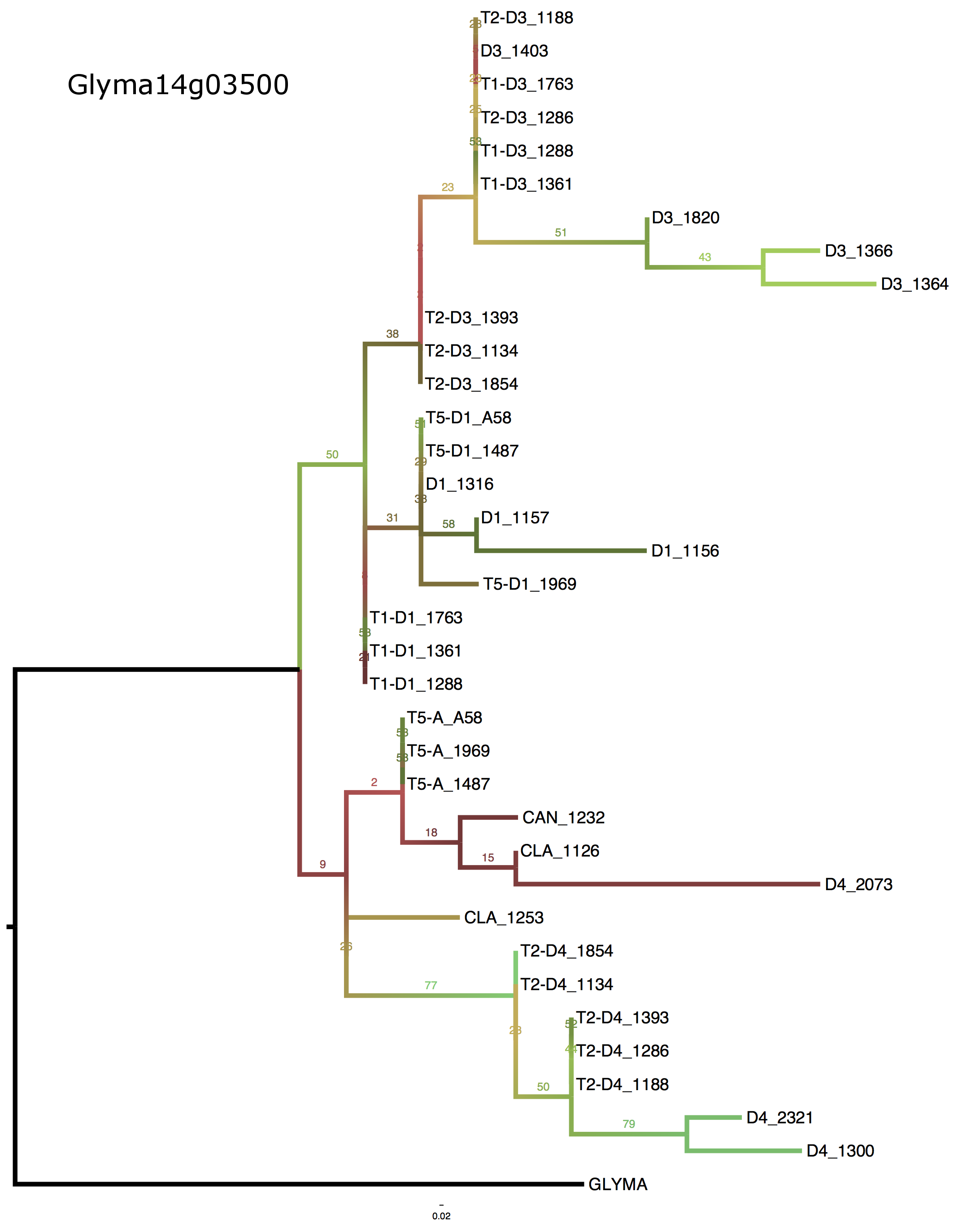

Supplement: Figures S13--S39 — MaximuThe color of the tree branch represents the bootstraping value (also with the same color). Genes are in the following order: Glyma01g35620 (Figure S13), Glyma02g11580 (Figure S14), Glyma03g29330 (Figure S15), Glyma03g36630 (Figure S16), Glyma04g39670 (Figure S17), Glyma05g05750 (Figure S18), Glyma05g09310 (Figure S19), Glyma05g26230 (Figure S20), Glyma05g37840 (Figure S21), Glyma06g18640 (Figure S22), Glyma07g03370 (Figure S23), Glyma07g17180 (Figure S24), Glyma10g42100 (Figure S25), Glyma11g13880 (Figure S26), Glyma11g33720 (Figure S27), Glyma12g04150 (Figure S28), Glyma12g12230 (Figure S29), Glyma13g17820 (Figure S30), Glyma14g03500 (Figure S31), Glyma16g00410 (Figure S32), Glyma16g01980 (Figure S33), Glyma16g04940 (Figure S34), Glyma18g04080 (Figure S35), Glyma19g03390 (Figure S36), Glyma19g32940 (Figure S37), Glyma20g24930 (Figure S38), Glyma20g32930 (Figure S39) [file peerj-02-391-s002.zip › Suplementary_Figures_S13-S39/Suplementary_Figure_31.png]

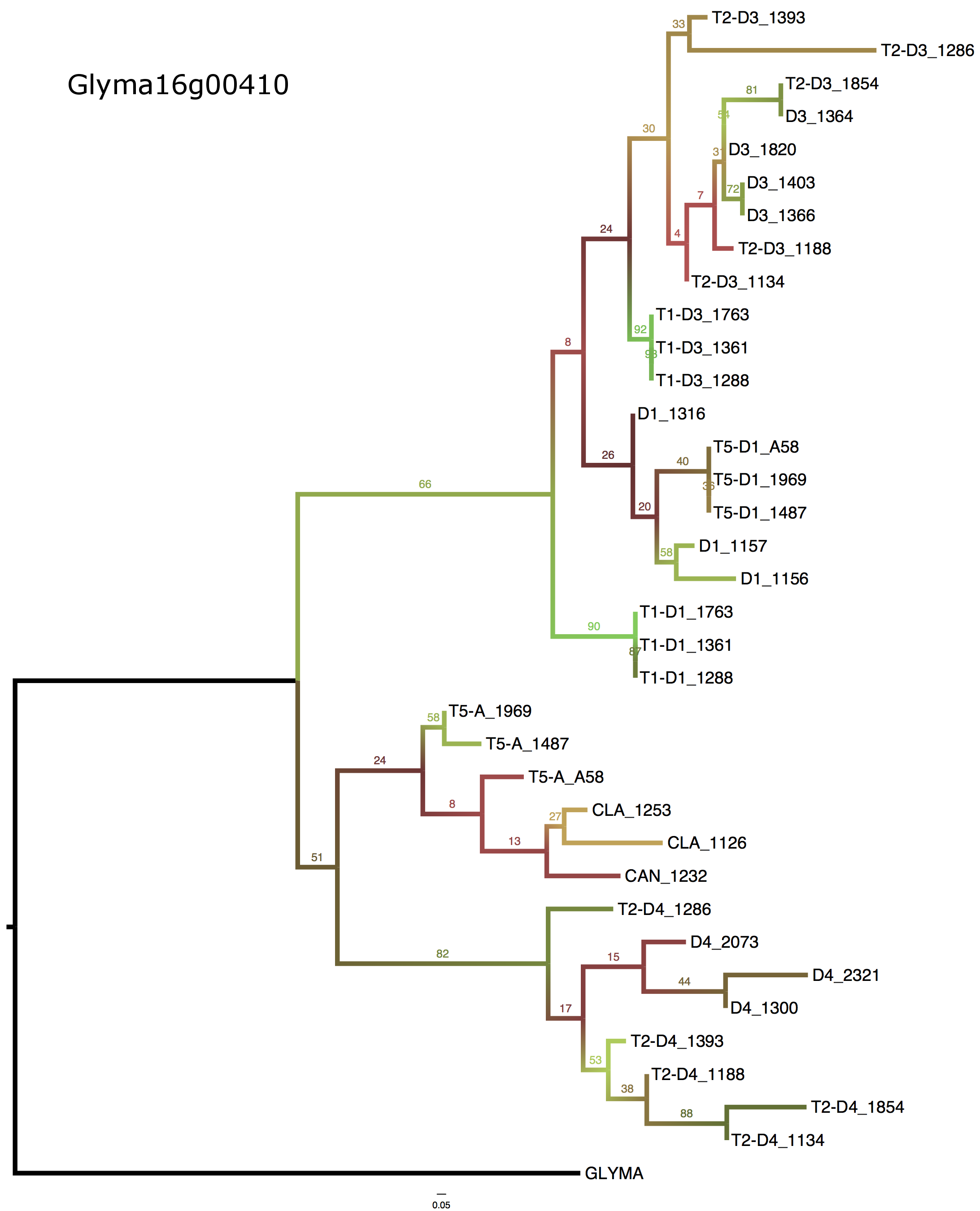

Supplement: Figures S13--S39 — MaximuThe color of the tree branch represents the bootstraping value (also with the same color). Genes are in the following order: Glyma01g35620 (Figure S13), Glyma02g11580 (Figure S14), Glyma03g29330 (Figure S15), Glyma03g36630 (Figure S16), Glyma04g39670 (Figure S17), Glyma05g05750 (Figure S18), Glyma05g09310 (Figure S19), Glyma05g26230 (Figure S20), Glyma05g37840 (Figure S21), Glyma06g18640 (Figure S22), Glyma07g03370 (Figure S23), Glyma07g17180 (Figure S24), Glyma10g42100 (Figure S25), Glyma11g13880 (Figure S26), Glyma11g33720 (Figure S27), Glyma12g04150 (Figure S28), Glyma12g12230 (Figure S29), Glyma13g17820 (Figure S30), Glyma14g03500 (Figure S31), Glyma16g00410 (Figure S32), Glyma16g01980 (Figure S33), Glyma16g04940 (Figure S34), Glyma18g04080 (Figure S35), Glyma19g03390 (Figure S36), Glyma19g32940 (Figure S37), Glyma20g24930 (Figure S38), Glyma20g32930 (Figure S39) [file peerj-02-391-s002.zip › Suplementary_Figures_S13-S39/Suplementary_Figure_32.png]

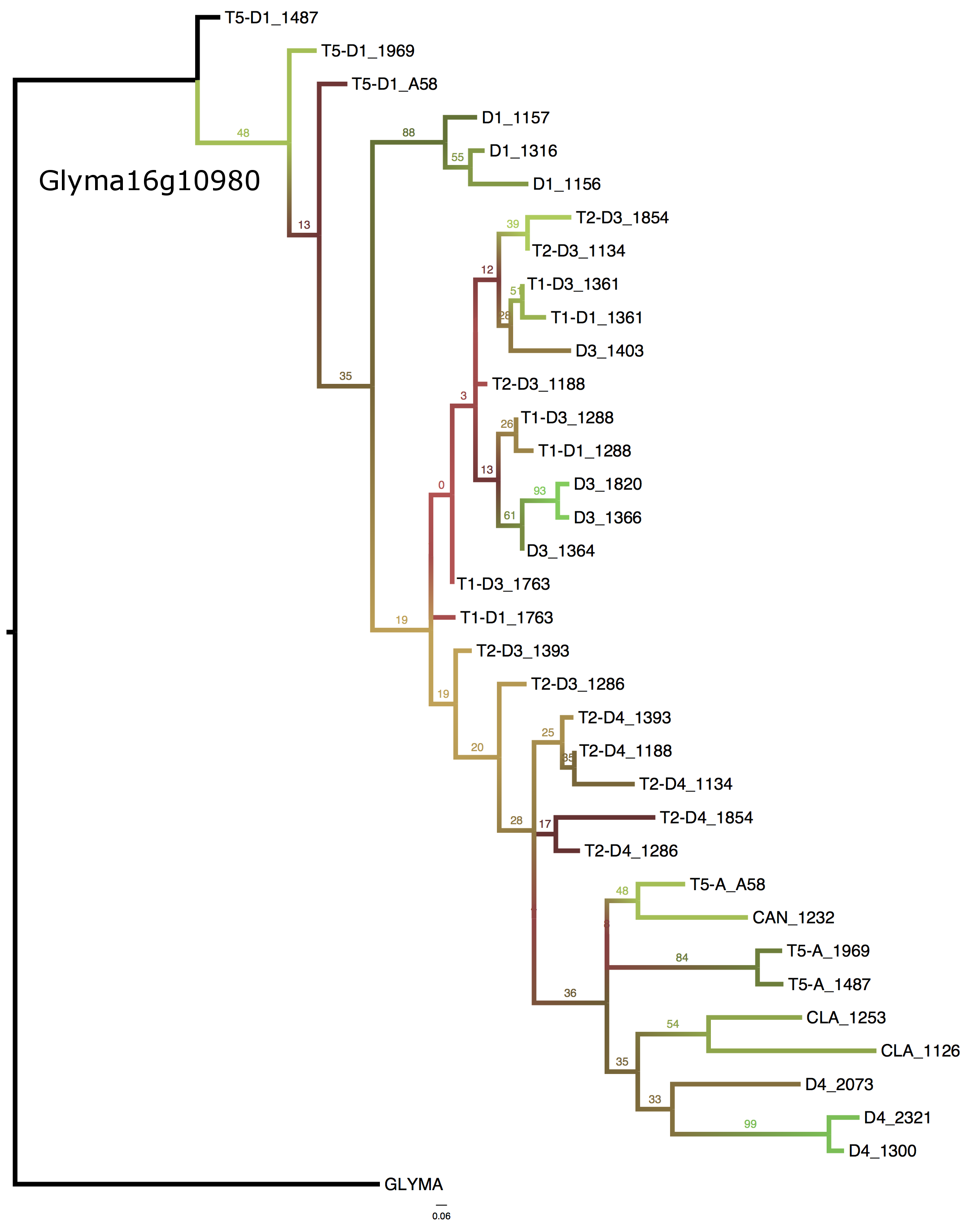

Supplement: Figures S13--S39 — MaximuThe color of the tree branch represents the bootstraping value (also with the same color). Genes are in the following order: Glyma01g35620 (Figure S13), Glyma02g11580 (Figure S14), Glyma03g29330 (Figure S15), Glyma03g36630 (Figure S16), Glyma04g39670 (Figure S17), Glyma05g05750 (Figure S18), Glyma05g09310 (Figure S19), Glyma05g26230 (Figure S20), Glyma05g37840 (Figure S21), Glyma06g18640 (Figure S22), Glyma07g03370 (Figure S23), Glyma07g17180 (Figure S24), Glyma10g42100 (Figure S25), Glyma11g13880 (Figure S26), Glyma11g33720 (Figure S27), Glyma12g04150 (Figure S28), Glyma12g12230 (Figure S29), Glyma13g17820 (Figure S30), Glyma14g03500 (Figure S31), Glyma16g00410 (Figure S32), Glyma16g01980 (Figure S33), Glyma16g04940 (Figure S34), Glyma18g04080 (Figure S35), Glyma19g03390 (Figure S36), Glyma19g32940 (Figure S37), Glyma20g24930 (Figure S38), Glyma20g32930 (Figure S39) [file peerj-02-391-s002.zip › Suplementary_Figures_S13-S39/Suplementary_Figure_33.png]

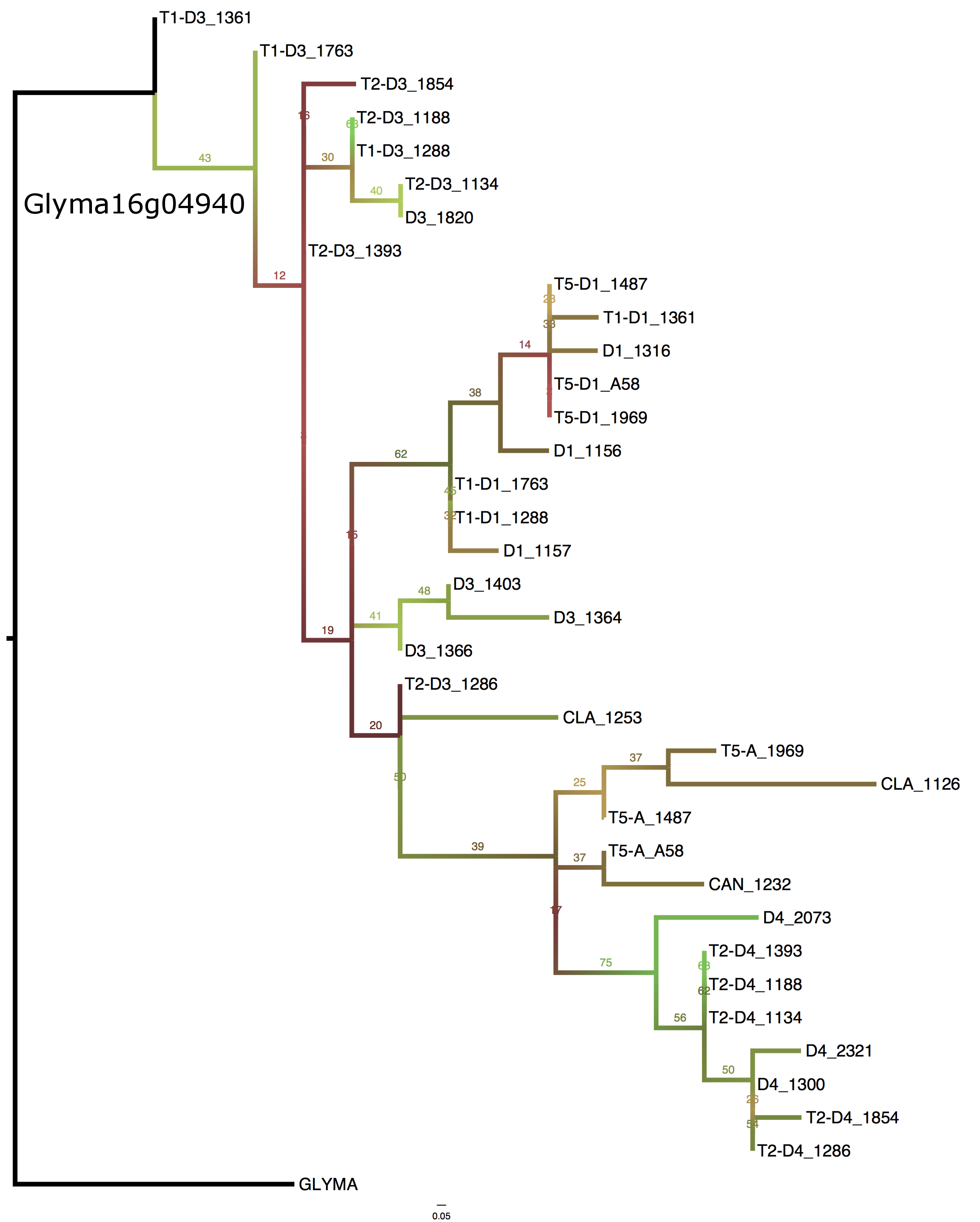

Supplement: Figures S13--S39 — MaximuThe color of the tree branch represents the bootstraping value (also with the same color). Genes are in the following order: Glyma01g35620 (Figure S13), Glyma02g11580 (Figure S14), Glyma03g29330 (Figure S15), Glyma03g36630 (Figure S16), Glyma04g39670 (Figure S17), Glyma05g05750 (Figure S18), Glyma05g09310 (Figure S19), Glyma05g26230 (Figure S20), Glyma05g37840 (Figure S21), Glyma06g18640 (Figure S22), Glyma07g03370 (Figure S23), Glyma07g17180 (Figure S24), Glyma10g42100 (Figure S25), Glyma11g13880 (Figure S26), Glyma11g33720 (Figure S27), Glyma12g04150 (Figure S28), Glyma12g12230 (Figure S29), Glyma13g17820 (Figure S30), Glyma14g03500 (Figure S31), Glyma16g00410 (Figure S32), Glyma16g01980 (Figure S33), Glyma16g04940 (Figure S34), Glyma18g04080 (Figure S35), Glyma19g03390 (Figure S36), Glyma19g32940 (Figure S37), Glyma20g24930 (Figure S38), Glyma20g32930 (Figure S39) [file peerj-02-391-s002.zip › Suplementary_Figures_S13-S39/Suplementary_Figure_34.png]

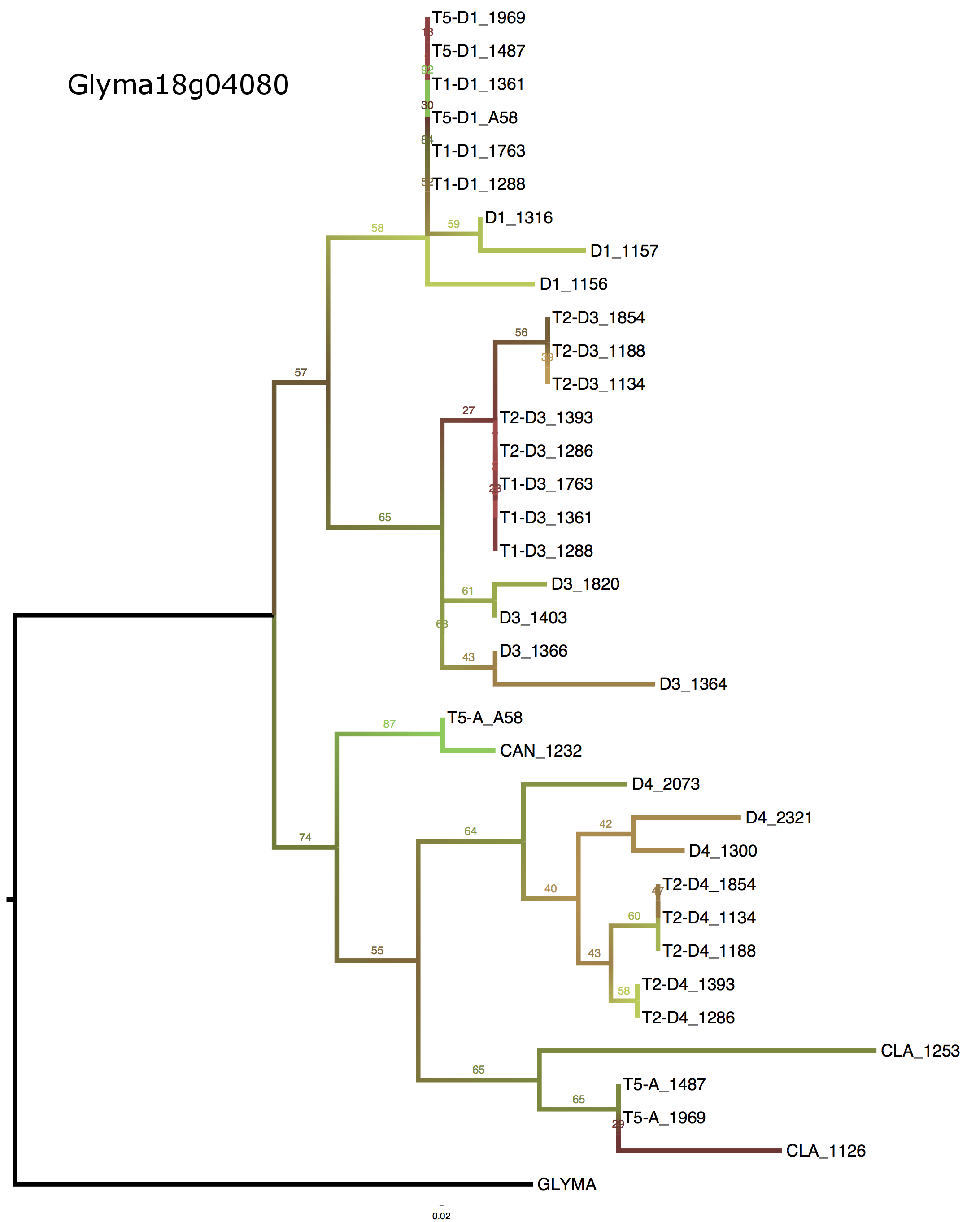

Supplement: Figures S13--S39 — MaximuThe color of the tree branch represents the bootstraping value (also with the same color). Genes are in the following order: Glyma01g35620 (Figure S13), Glyma02g11580 (Figure S14), Glyma03g29330 (Figure S15), Glyma03g36630 (Figure S16), Glyma04g39670 (Figure S17), Glyma05g05750 (Figure S18), Glyma05g09310 (Figure S19), Glyma05g26230 (Figure S20), Glyma05g37840 (Figure S21), Glyma06g18640 (Figure S22), Glyma07g03370 (Figure S23), Glyma07g17180 (Figure S24), Glyma10g42100 (Figure S25), Glyma11g13880 (Figure S26), Glyma11g33720 (Figure S27), Glyma12g04150 (Figure S28), Glyma12g12230 (Figure S29), Glyma13g17820 (Figure S30), Glyma14g03500 (Figure S31), Glyma16g00410 (Figure S32), Glyma16g01980 (Figure S33), Glyma16g04940 (Figure S34), Glyma18g04080 (Figure S35), Glyma19g03390 (Figure S36), Glyma19g32940 (Figure S37), Glyma20g24930 (Figure S38), Glyma20g32930 (Figure S39) [file peerj-02-391-s002.zip › Suplementary_Figures_S13-S39/Suplementary_Figure_35.png]

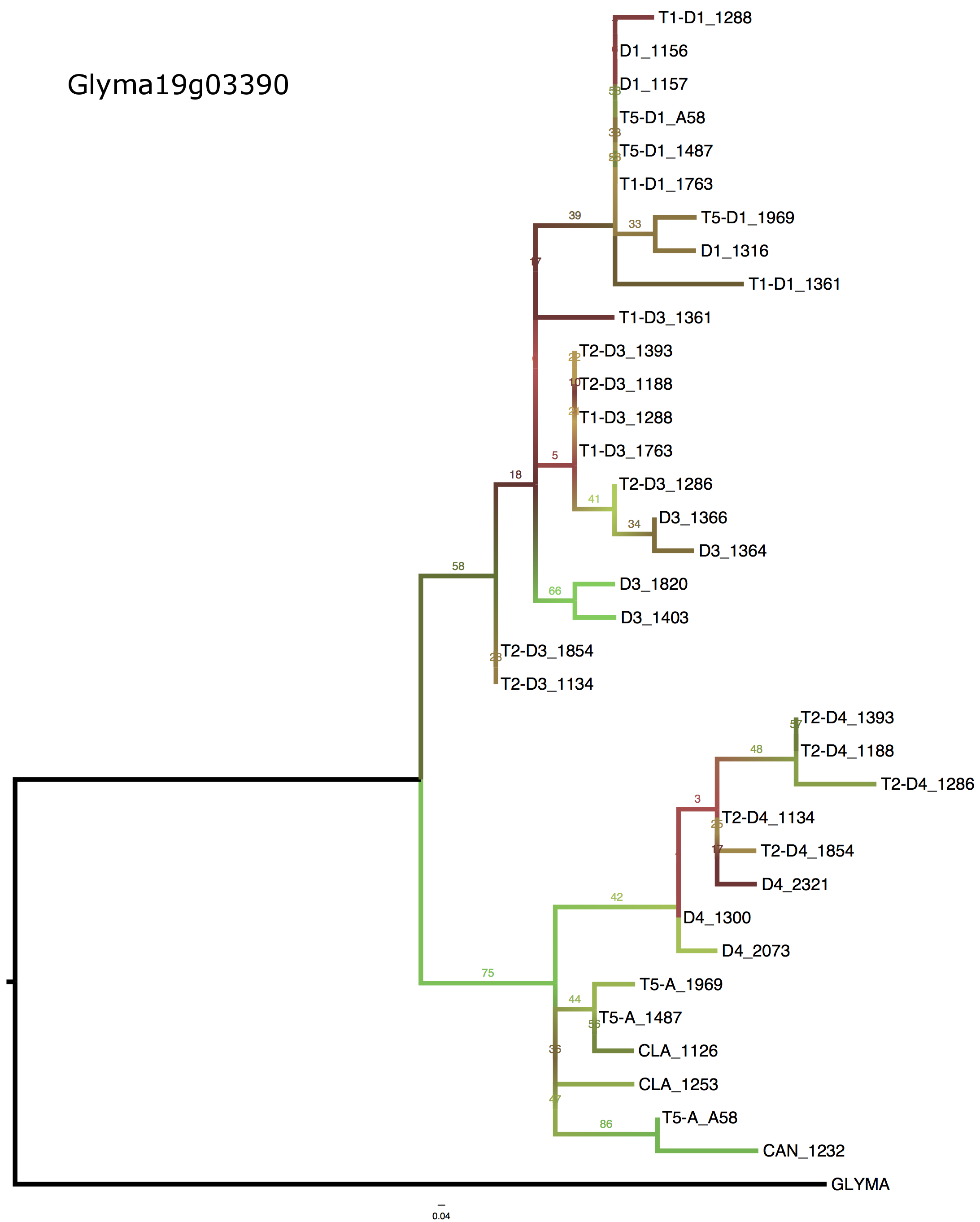

Supplement: Figures S13--S39 — MaximuThe color of the tree branch represents the bootstraping value (also with the same color). Genes are in the following order: Glyma01g35620 (Figure S13), Glyma02g11580 (Figure S14), Glyma03g29330 (Figure S15), Glyma03g36630 (Figure S16), Glyma04g39670 (Figure S17), Glyma05g05750 (Figure S18), Glyma05g09310 (Figure S19), Glyma05g26230 (Figure S20), Glyma05g37840 (Figure S21), Glyma06g18640 (Figure S22), Glyma07g03370 (Figure S23), Glyma07g17180 (Figure S24), Glyma10g42100 (Figure S25), Glyma11g13880 (Figure S26), Glyma11g33720 (Figure S27), Glyma12g04150 (Figure S28), Glyma12g12230 (Figure S29), Glyma13g17820 (Figure S30), Glyma14g03500 (Figure S31), Glyma16g00410 (Figure S32), Glyma16g01980 (Figure S33), Glyma16g04940 (Figure S34), Glyma18g04080 (Figure S35), Glyma19g03390 (Figure S36), Glyma19g32940 (Figure S37), Glyma20g24930 (Figure S38), Glyma20g32930 (Figure S39) [file peerj-02-391-s002.zip › Suplementary_Figures_S13-S39/Suplementary_Figure_36.png]

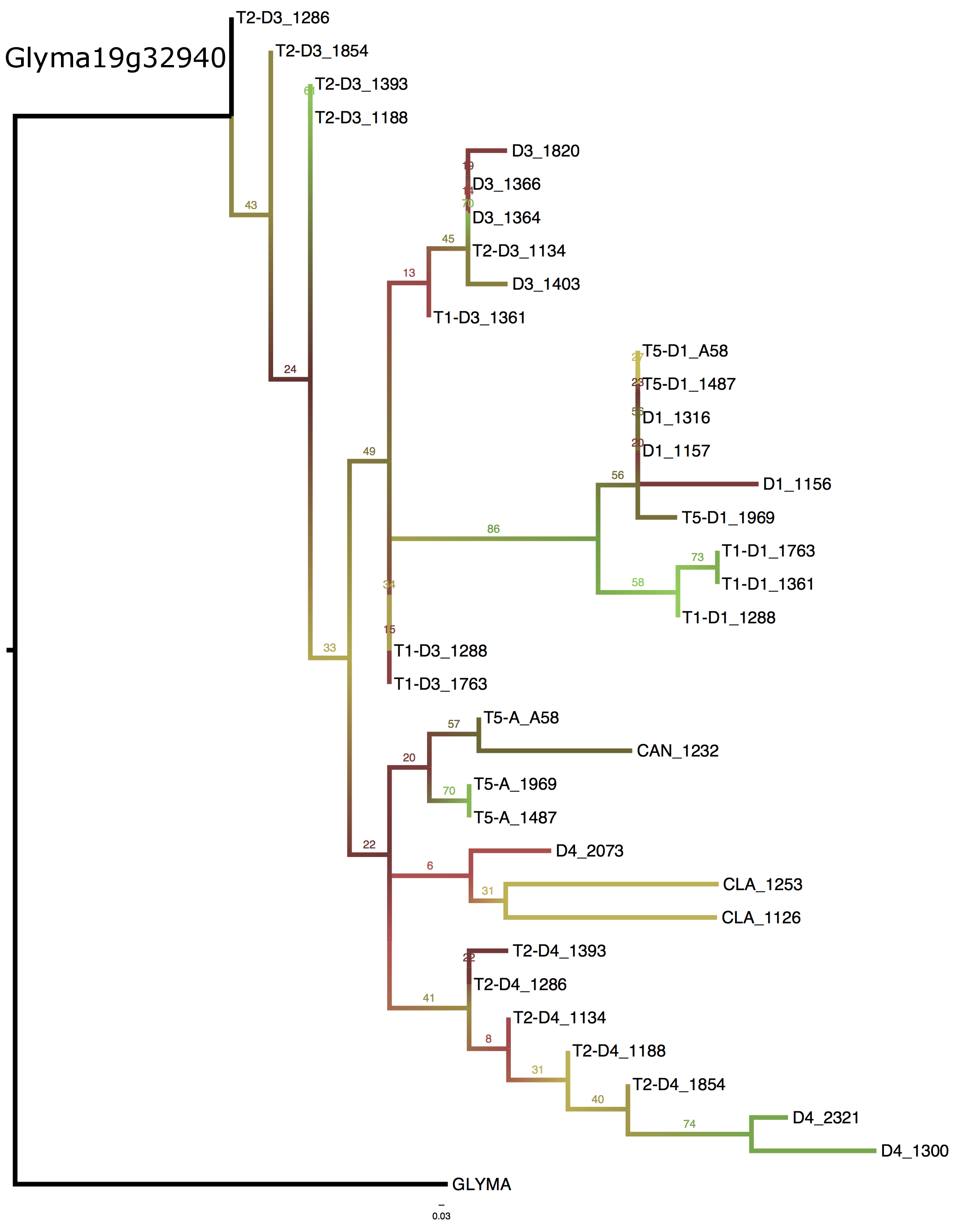

Supplement: Figures S13--S39 — MaximuThe color of the tree branch represents the bootstraping value (also with the same color). Genes are in the following order: Glyma01g35620 (Figure S13), Glyma02g11580 (Figure S14), Glyma03g29330 (Figure S15), Glyma03g36630 (Figure S16), Glyma04g39670 (Figure S17), Glyma05g05750 (Figure S18), Glyma05g09310 (Figure S19), Glyma05g26230 (Figure S20), Glyma05g37840 (Figure S21), Glyma06g18640 (Figure S22), Glyma07g03370 (Figure S23), Glyma07g17180 (Figure S24), Glyma10g42100 (Figure S25), Glyma11g13880 (Figure S26), Glyma11g33720 (Figure S27), Glyma12g04150 (Figure S28), Glyma12g12230 (Figure S29), Glyma13g17820 (Figure S30), Glyma14g03500 (Figure S31), Glyma16g00410 (Figure S32), Glyma16g01980 (Figure S33), Glyma16g04940 (Figure S34), Glyma18g04080 (Figure S35), Glyma19g03390 (Figure S36), Glyma19g32940 (Figure S37), Glyma20g24930 (Figure S38), Glyma20g32930 (Figure S39) [file peerj-02-391-s002.zip › Suplementary_Figures_S13-S39/Suplementary_Figure_37.png]

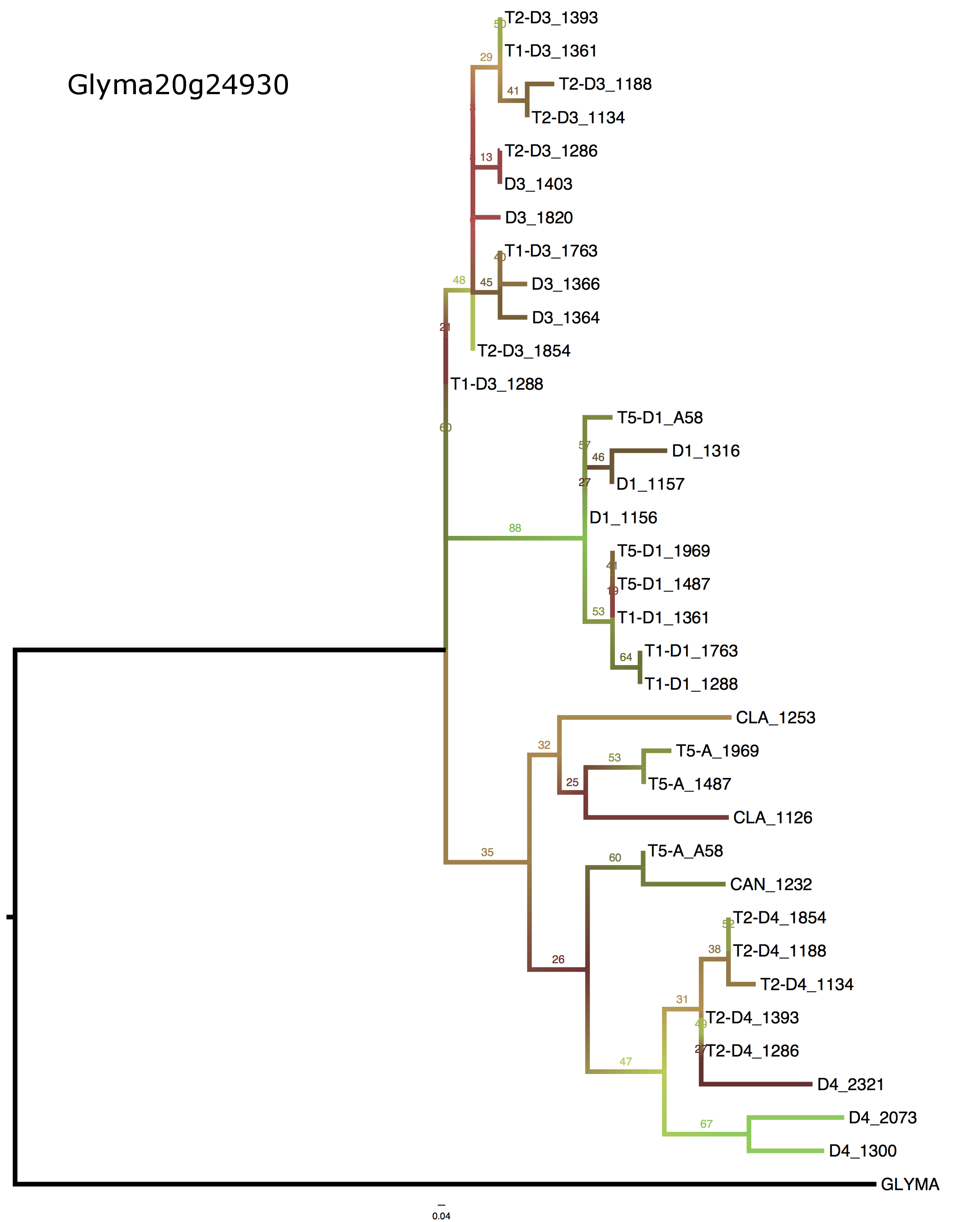

Supplement: Figures S13--S39 — MaximuThe color of the tree branch represents the bootstraping value (also with the same color). Genes are in the following order: Glyma01g35620 (Figure S13), Glyma02g11580 (Figure S14), Glyma03g29330 (Figure S15), Glyma03g36630 (Figure S16), Glyma04g39670 (Figure S17), Glyma05g05750 (Figure S18), Glyma05g09310 (Figure S19), Glyma05g26230 (Figure S20), Glyma05g37840 (Figure S21), Glyma06g18640 (Figure S22), Glyma07g03370 (Figure S23), Glyma07g17180 (Figure S24), Glyma10g42100 (Figure S25), Glyma11g13880 (Figure S26), Glyma11g33720 (Figure S27), Glyma12g04150 (Figure S28), Glyma12g12230 (Figure S29), Glyma13g17820 (Figure S30), Glyma14g03500 (Figure S31), Glyma16g00410 (Figure S32), Glyma16g01980 (Figure S33), Glyma16g04940 (Figure S34), Glyma18g04080 (Figure S35), Glyma19g03390 (Figure S36), Glyma19g32940 (Figure S37), Glyma20g24930 (Figure S38), Glyma20g32930 (Figure S39) [file peerj-02-391-s002.zip › Suplementary_Figures_S13-S39/Suplementary_Figure_38.png]

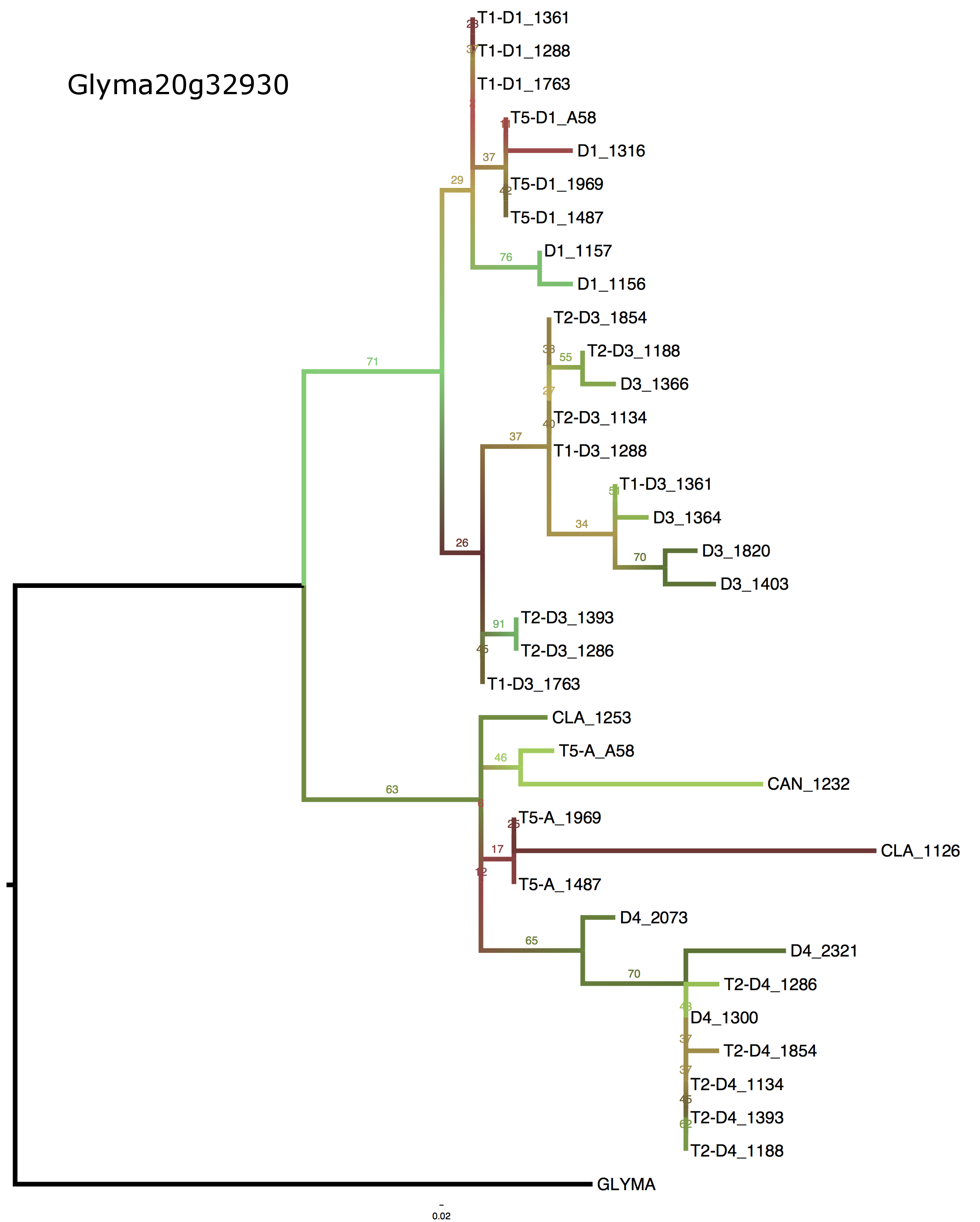

Supplement: Figures S13--S39 — MaximuThe color of the tree branch represents the bootstraping value (also with the same color). Genes are in the following order: Glyma01g35620 (Figure S13), Glyma02g11580 (Figure S14), Glyma03g29330 (Figure S15), Glyma03g36630 (Figure S16), Glyma04g39670 (Figure S17), Glyma05g05750 (Figure S18), Glyma05g09310 (Figure S19), Glyma05g26230 (Figure S20), Glyma05g37840 (Figure S21), Glyma06g18640 (Figure S22), Glyma07g03370 (Figure S23), Glyma07g17180 (Figure S24), Glyma10g42100 (Figure S25), Glyma11g13880 (Figure S26), Glyma11g33720 (Figure S27), Glyma12g04150 (Figure S28), Glyma12g12230 (Figure S29), Glyma13g17820 (Figure S30), Glyma14g03500 (Figure S31), Glyma16g00410 (Figure S32), Glyma16g01980 (Figure S33), Glyma16g04940 (Figure S34), Glyma18g04080 (Figure S35), Glyma19g03390 (Figure S36), Glyma19g32940 (Figure S37), Glyma20g24930 (Figure S38), Glyma20g32930 (Figure S39) [file peerj-02-391-s002.zip › Suplementary_Figures_S13-S39/Suplementary_Figure_39.png]

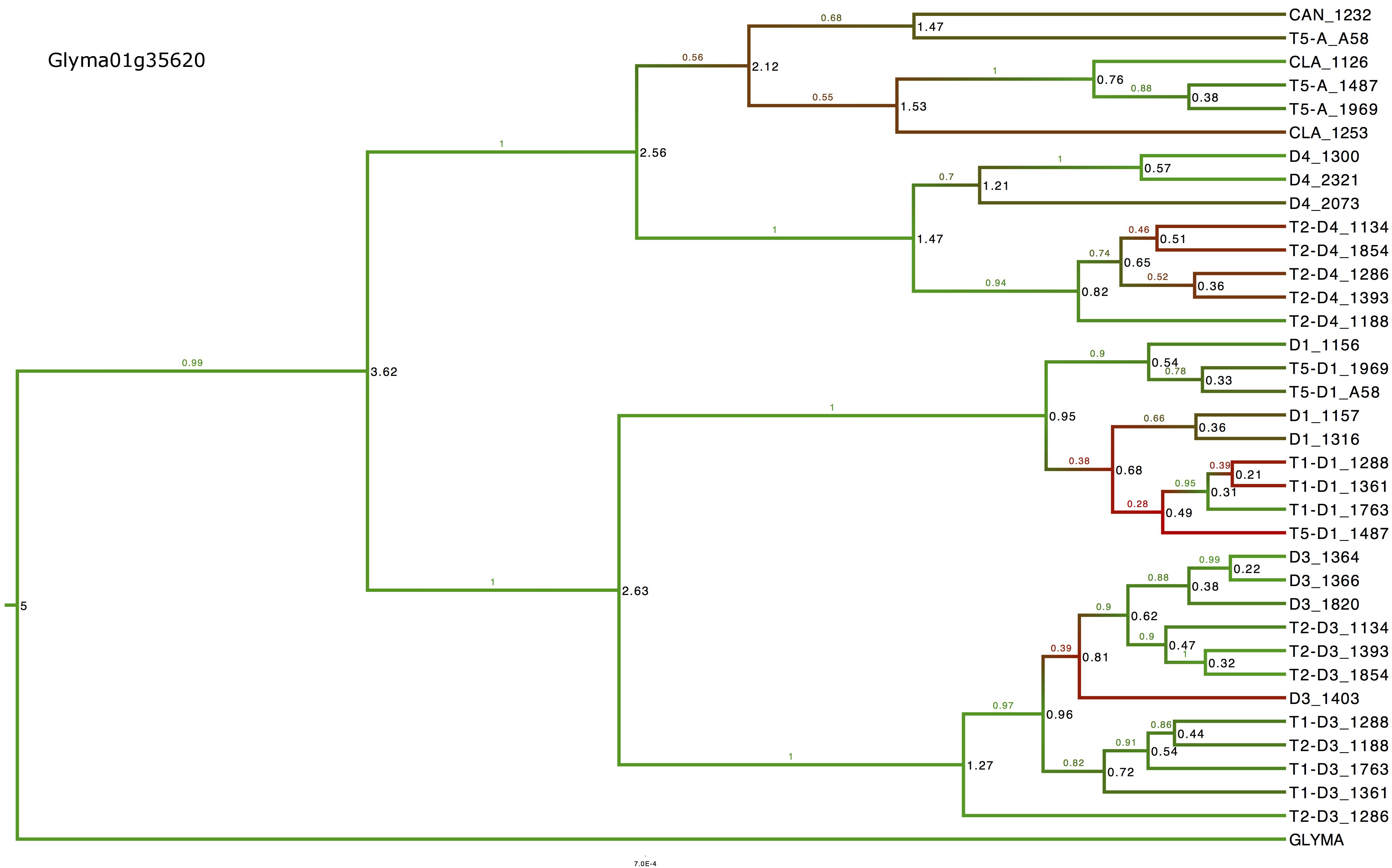

Supplement: Figures S41--S67 — The color of the tree branch represents the posterior probability value (also with the same color). Genes are in the following order: Glyma01g35620 (Figure S41), Glyma02g11580 (Figure S42), Glyma03g29330 (Figure S43), Glyma03g36630 (Figure S44), Glyma04g39670 (Figure S45), Glyma05g05750 (Figure S46), Glyma05g09310 (Figure S47), Glyma05g26230 (Figure S48), Glyma05g37840 (Figure S49), Glyma06g18640 (Figure S50), Glyma07g03370 (Figure S51), Glyma07g17180 (Figure S52), Glyma10g42100 (Figure S53), Glyma11g13880 (Figure S54), Glyma11g33720 (Figure S55), Glyma12g04150 (Figure S56), Glyma12g12230 (Figure S57), Glyma13g17820 (Figure S58), Glyma14g03500 (Figure S59), Glyma16g00410 (Figure S60), Glyma16g01980 (Figure S61), Glyma16g04940 (Figure S62), Glyma18g04080 (Figure S63), Glyma19g03390 (Figure S64), Glyma19g32940 (Figure S65), Glyma20g24930 (Figure S66), Glyma20g32930 (Figure S67). [file peerj-02-391-s003.zip › Suplementary_Figures_S41-S67/Suplementary_Figure_41.png]

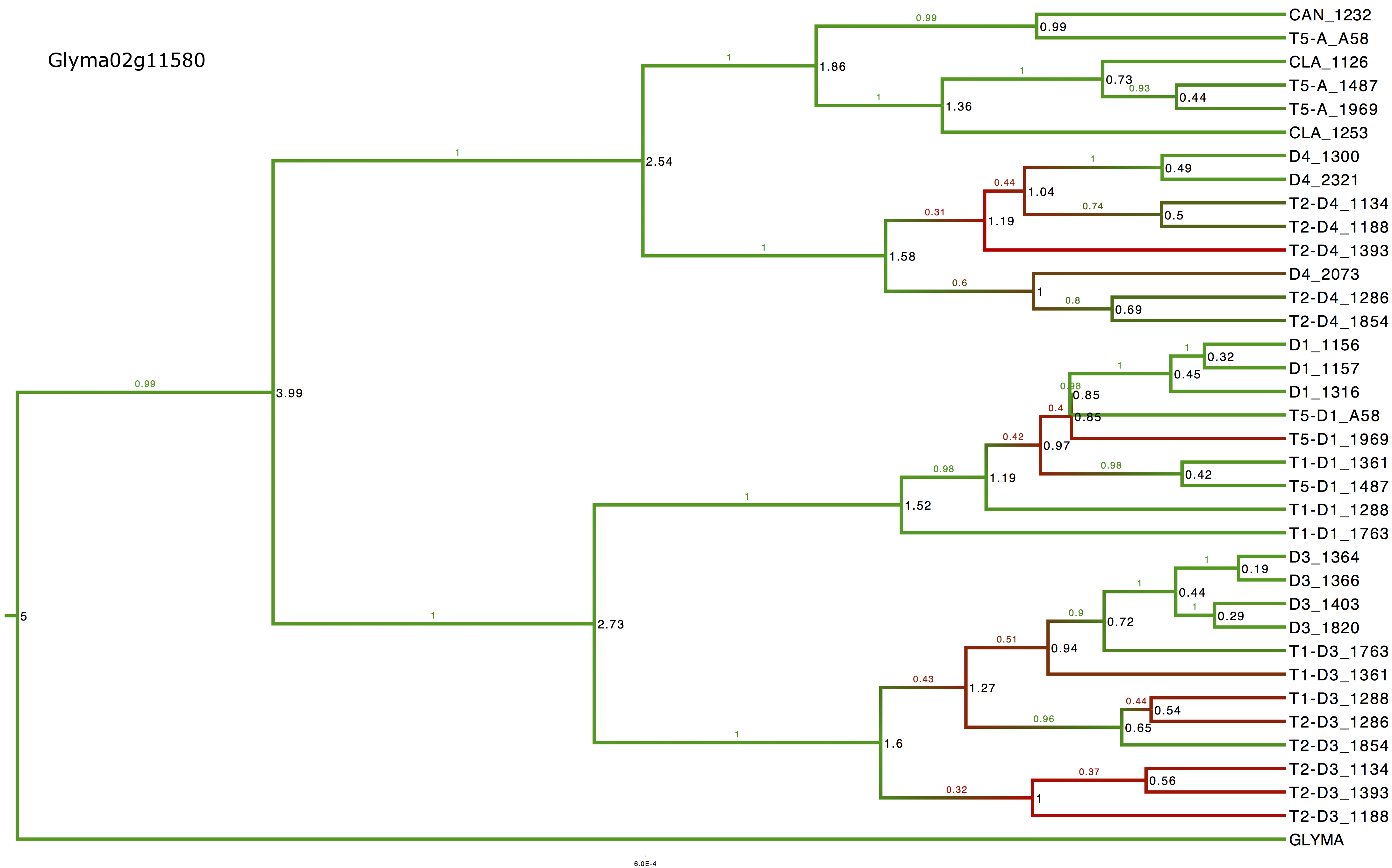

Supplement: Figures S41--S67 — The color of the tree branch represents the posterior probability value (also with the same color). Genes are in the following order: Glyma01g35620 (Figure S41), Glyma02g11580 (Figure S42), Glyma03g29330 (Figure S43), Glyma03g36630 (Figure S44), Glyma04g39670 (Figure S45), Glyma05g05750 (Figure S46), Glyma05g09310 (Figure S47), Glyma05g26230 (Figure S48), Glyma05g37840 (Figure S49), Glyma06g18640 (Figure S50), Glyma07g03370 (Figure S51), Glyma07g17180 (Figure S52), Glyma10g42100 (Figure S53), Glyma11g13880 (Figure S54), Glyma11g33720 (Figure S55), Glyma12g04150 (Figure S56), Glyma12g12230 (Figure S57), Glyma13g17820 (Figure S58), Glyma14g03500 (Figure S59), Glyma16g00410 (Figure S60), Glyma16g01980 (Figure S61), Glyma16g04940 (Figure S62), Glyma18g04080 (Figure S63), Glyma19g03390 (Figure S64), Glyma19g32940 (Figure S65), Glyma20g24930 (Figure S66), Glyma20g32930 (Figure S67). [file peerj-02-391-s003.zip › Suplementary_Figures_S41-S67/Suplementary_Figure_42.png]

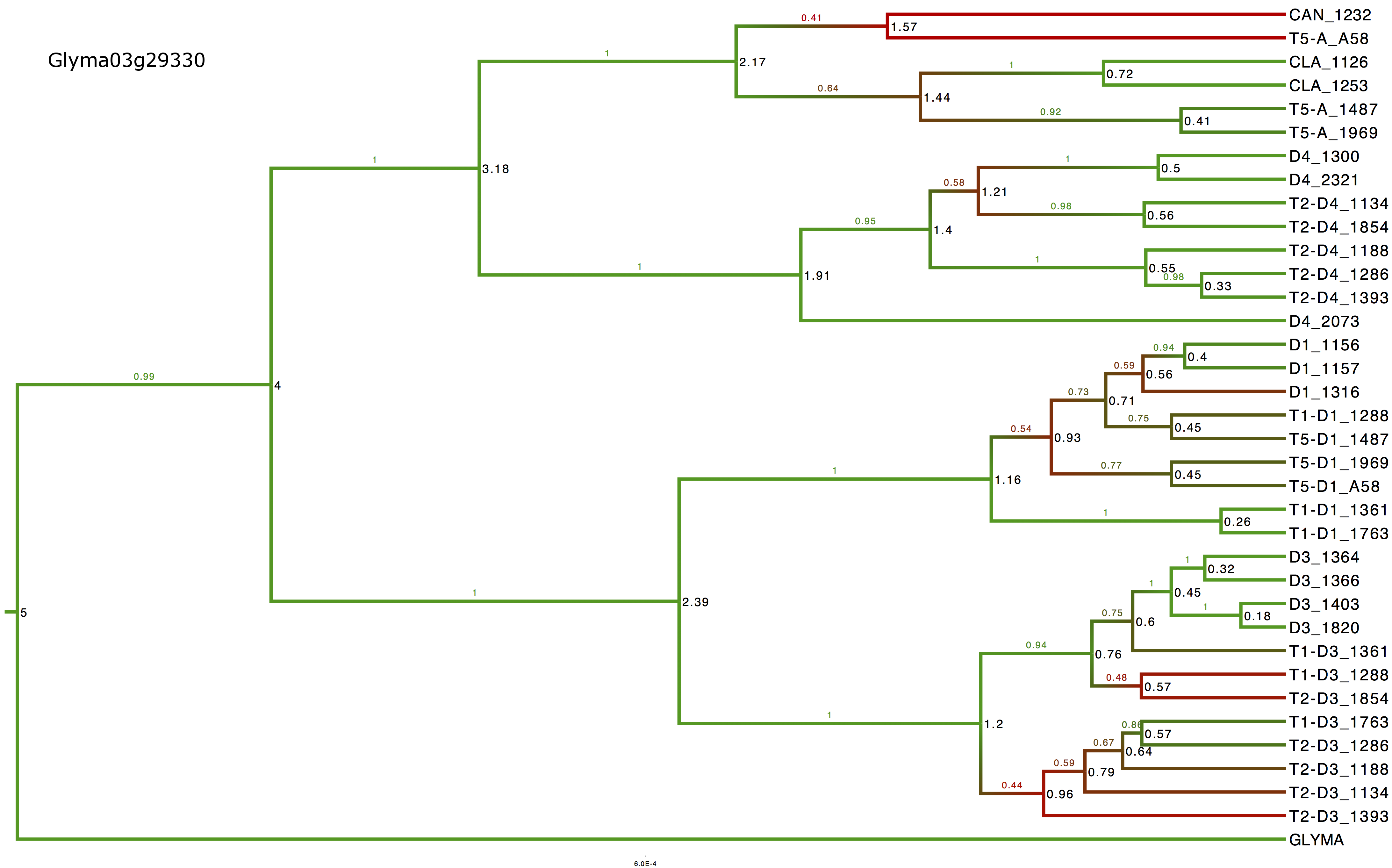

Supplement: Figures S41--S67 — The color of the tree branch represents the posterior probability value (also with the same color). Genes are in the following order: Glyma01g35620 (Figure S41), Glyma02g11580 (Figure S42), Glyma03g29330 (Figure S43), Glyma03g36630 (Figure S44), Glyma04g39670 (Figure S45), Glyma05g05750 (Figure S46), Glyma05g09310 (Figure S47), Glyma05g26230 (Figure S48), Glyma05g37840 (Figure S49), Glyma06g18640 (Figure S50), Glyma07g03370 (Figure S51), Glyma07g17180 (Figure S52), Glyma10g42100 (Figure S53), Glyma11g13880 (Figure S54), Glyma11g33720 (Figure S55), Glyma12g04150 (Figure S56), Glyma12g12230 (Figure S57), Glyma13g17820 (Figure S58), Glyma14g03500 (Figure S59), Glyma16g00410 (Figure S60), Glyma16g01980 (Figure S61), Glyma16g04940 (Figure S62), Glyma18g04080 (Figure S63), Glyma19g03390 (Figure S64), Glyma19g32940 (Figure S65), Glyma20g24930 (Figure S66), Glyma20g32930 (Figure S67). [file peerj-02-391-s003.zip › Suplementary_Figures_S41-S67/Suplementary_Figure_43.png]

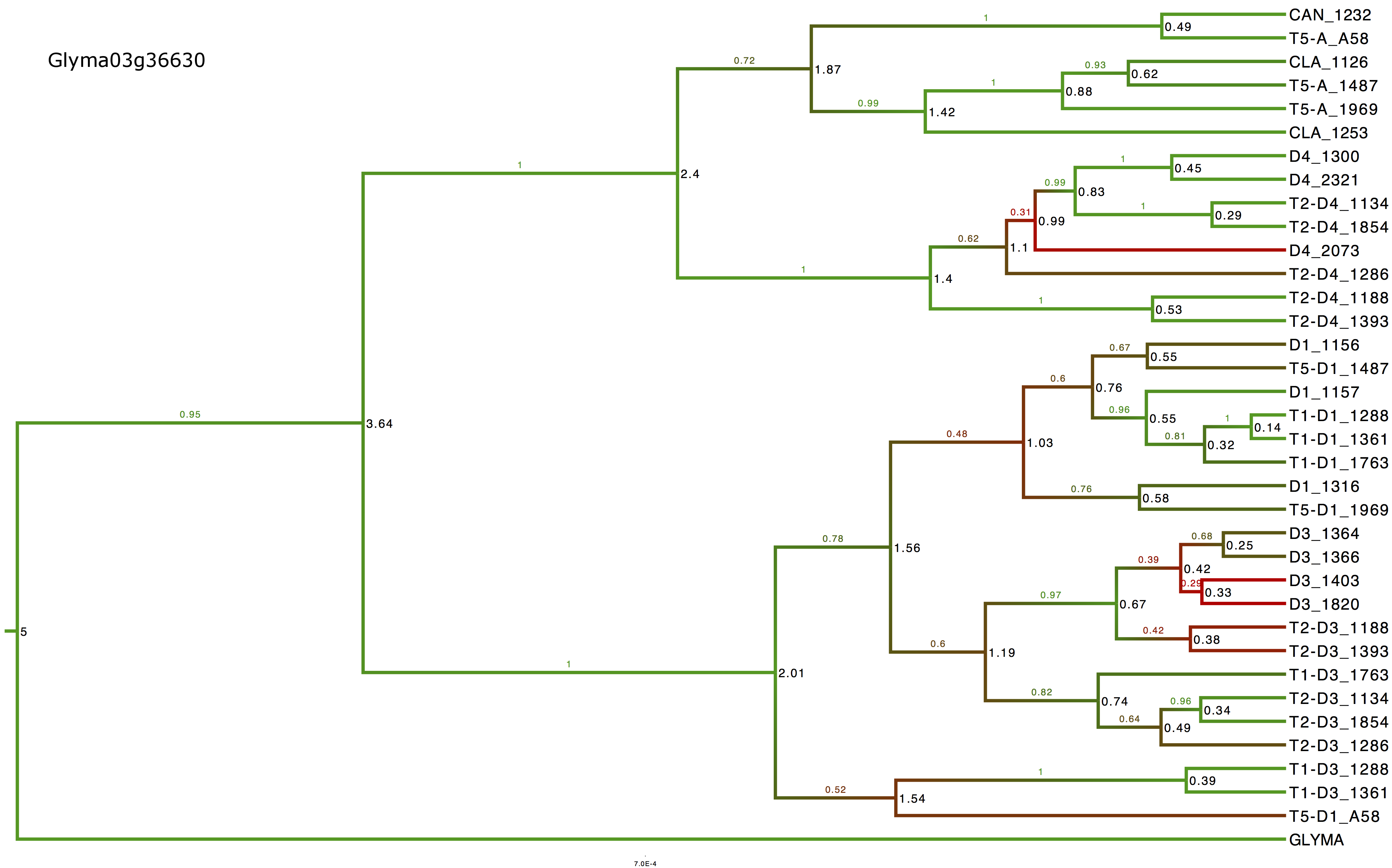

Supplement: Figures S41--S67 — The color of the tree branch represents the posterior probability value (also with the same color). Genes are in the following order: Glyma01g35620 (Figure S41), Glyma02g11580 (Figure S42), Glyma03g29330 (Figure S43), Glyma03g36630 (Figure S44), Glyma04g39670 (Figure S45), Glyma05g05750 (Figure S46), Glyma05g09310 (Figure S47), Glyma05g26230 (Figure S48), Glyma05g37840 (Figure S49), Glyma06g18640 (Figure S50), Glyma07g03370 (Figure S51), Glyma07g17180 (Figure S52), Glyma10g42100 (Figure S53), Glyma11g13880 (Figure S54), Glyma11g33720 (Figure S55), Glyma12g04150 (Figure S56), Glyma12g12230 (Figure S57), Glyma13g17820 (Figure S58), Glyma14g03500 (Figure S59), Glyma16g00410 (Figure S60), Glyma16g01980 (Figure S61), Glyma16g04940 (Figure S62), Glyma18g04080 (Figure S63), Glyma19g03390 (Figure S64), Glyma19g32940 (Figure S65), Glyma20g24930 (Figure S66), Glyma20g32930 (Figure S67). [file peerj-02-391-s003.zip › Suplementary_Figures_S41-S67/Suplementary_Figure_44.png]

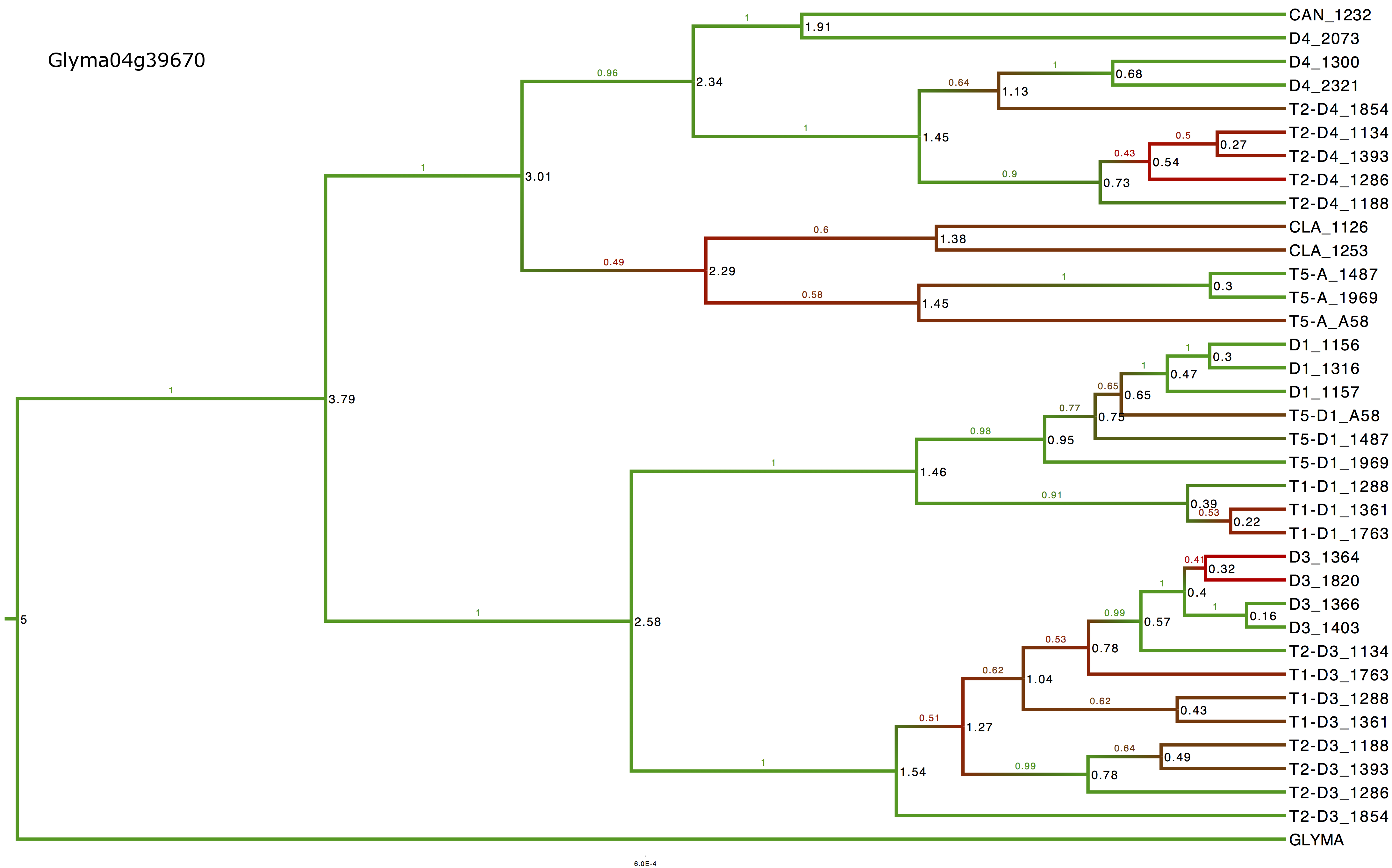

Supplement: Figures S41--S67 — The color of the tree branch represents the posterior probability value (also with the same color). Genes are in the following order: Glyma01g35620 (Figure S41), Glyma02g11580 (Figure S42), Glyma03g29330 (Figure S43), Glyma03g36630 (Figure S44), Glyma04g39670 (Figure S45), Glyma05g05750 (Figure S46), Glyma05g09310 (Figure S47), Glyma05g26230 (Figure S48), Glyma05g37840 (Figure S49), Glyma06g18640 (Figure S50), Glyma07g03370 (Figure S51), Glyma07g17180 (Figure S52), Glyma10g42100 (Figure S53), Glyma11g13880 (Figure S54), Glyma11g33720 (Figure S55), Glyma12g04150 (Figure S56), Glyma12g12230 (Figure S57), Glyma13g17820 (Figure S58), Glyma14g03500 (Figure S59), Glyma16g00410 (Figure S60), Glyma16g01980 (Figure S61), Glyma16g04940 (Figure S62), Glyma18g04080 (Figure S63), Glyma19g03390 (Figure S64), Glyma19g32940 (Figure S65), Glyma20g24930 (Figure S66), Glyma20g32930 (Figure S67). [file peerj-02-391-s003.zip › Suplementary_Figures_S41-S67/Suplementary_Figure_45.png]

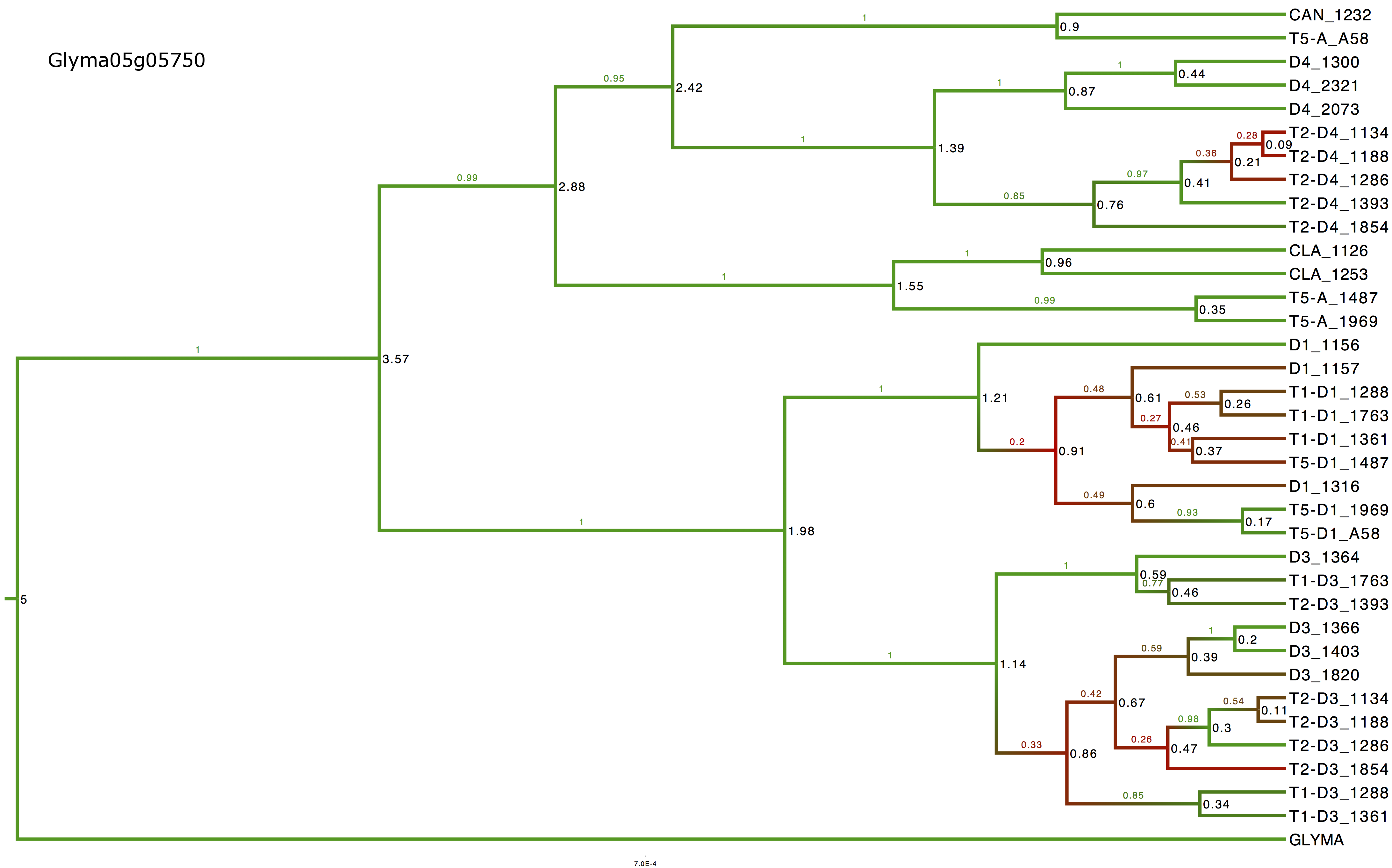

Supplement: Figures S41--S67 — The color of the tree branch represents the posterior probability value (also with the same color). Genes are in the following order: Glyma01g35620 (Figure S41), Glyma02g11580 (Figure S42), Glyma03g29330 (Figure S43), Glyma03g36630 (Figure S44), Glyma04g39670 (Figure S45), Glyma05g05750 (Figure S46), Glyma05g09310 (Figure S47), Glyma05g26230 (Figure S48), Glyma05g37840 (Figure S49), Glyma06g18640 (Figure S50), Glyma07g03370 (Figure S51), Glyma07g17180 (Figure S52), Glyma10g42100 (Figure S53), Glyma11g13880 (Figure S54), Glyma11g33720 (Figure S55), Glyma12g04150 (Figure S56), Glyma12g12230 (Figure S57), Glyma13g17820 (Figure S58), Glyma14g03500 (Figure S59), Glyma16g00410 (Figure S60), Glyma16g01980 (Figure S61), Glyma16g04940 (Figure S62), Glyma18g04080 (Figure S63), Glyma19g03390 (Figure S64), Glyma19g32940 (Figure S65), Glyma20g24930 (Figure S66), Glyma20g32930 (Figure S67). [file peerj-02-391-s003.zip › Suplementary_Figures_S41-S67/Suplementary_Figure_46.png]

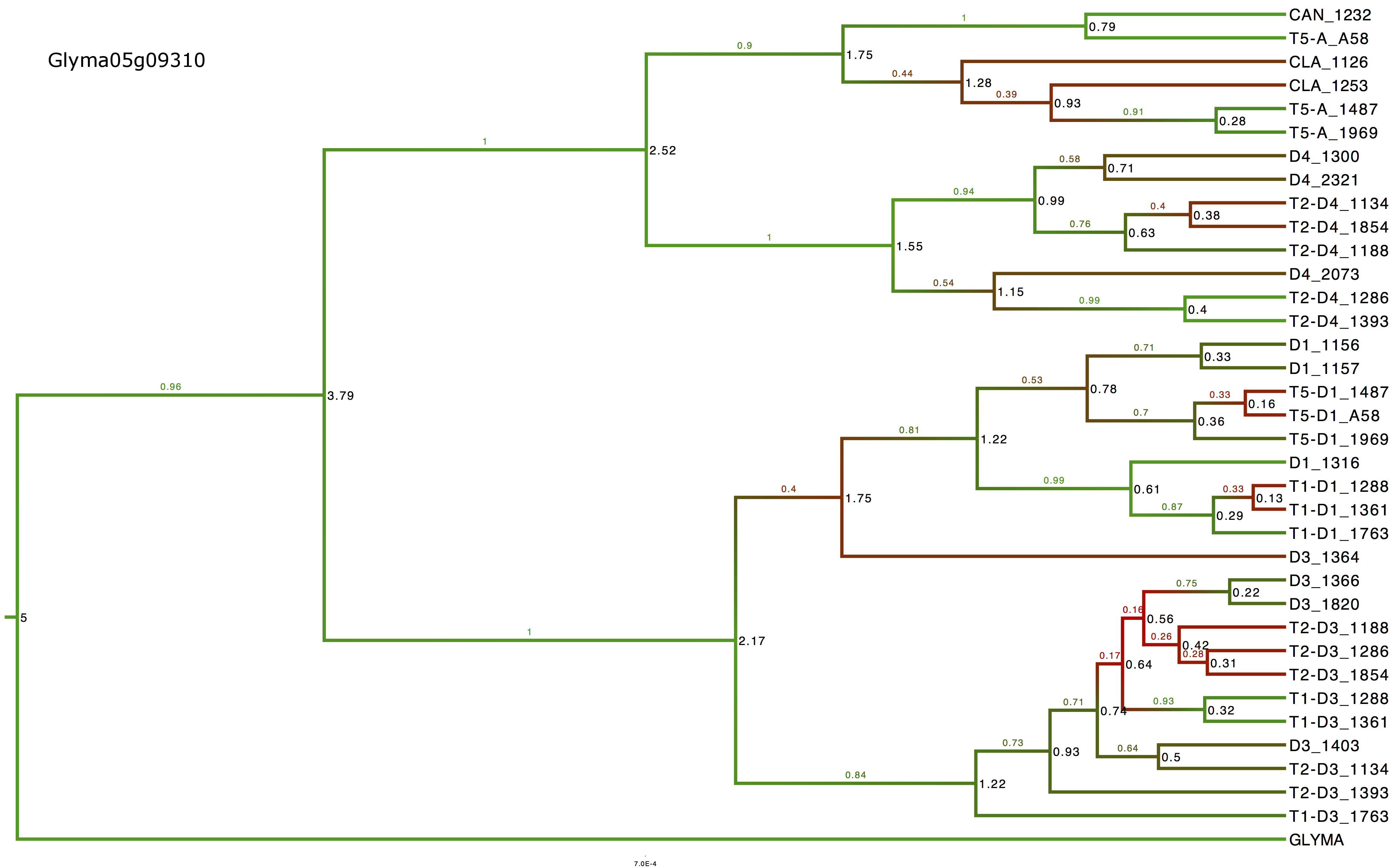

Supplement: Figures S41--S67 — The color of the tree branch represents the posterior probability value (also with the same color). Genes are in the following order: Glyma01g35620 (Figure S41), Glyma02g11580 (Figure S42), Glyma03g29330 (Figure S43), Glyma03g36630 (Figure S44), Glyma04g39670 (Figure S45), Glyma05g05750 (Figure S46), Glyma05g09310 (Figure S47), Glyma05g26230 (Figure S48), Glyma05g37840 (Figure S49), Glyma06g18640 (Figure S50), Glyma07g03370 (Figure S51), Glyma07g17180 (Figure S52), Glyma10g42100 (Figure S53), Glyma11g13880 (Figure S54), Glyma11g33720 (Figure S55), Glyma12g04150 (Figure S56), Glyma12g12230 (Figure S57), Glyma13g17820 (Figure S58), Glyma14g03500 (Figure S59), Glyma16g00410 (Figure S60), Glyma16g01980 (Figure S61), Glyma16g04940 (Figure S62), Glyma18g04080 (Figure S63), Glyma19g03390 (Figure S64), Glyma19g32940 (Figure S65), Glyma20g24930 (Figure S66), Glyma20g32930 (Figure S67). [file peerj-02-391-s003.zip › Suplementary_Figures_S41-S67/Suplementary_Figure_47.png]

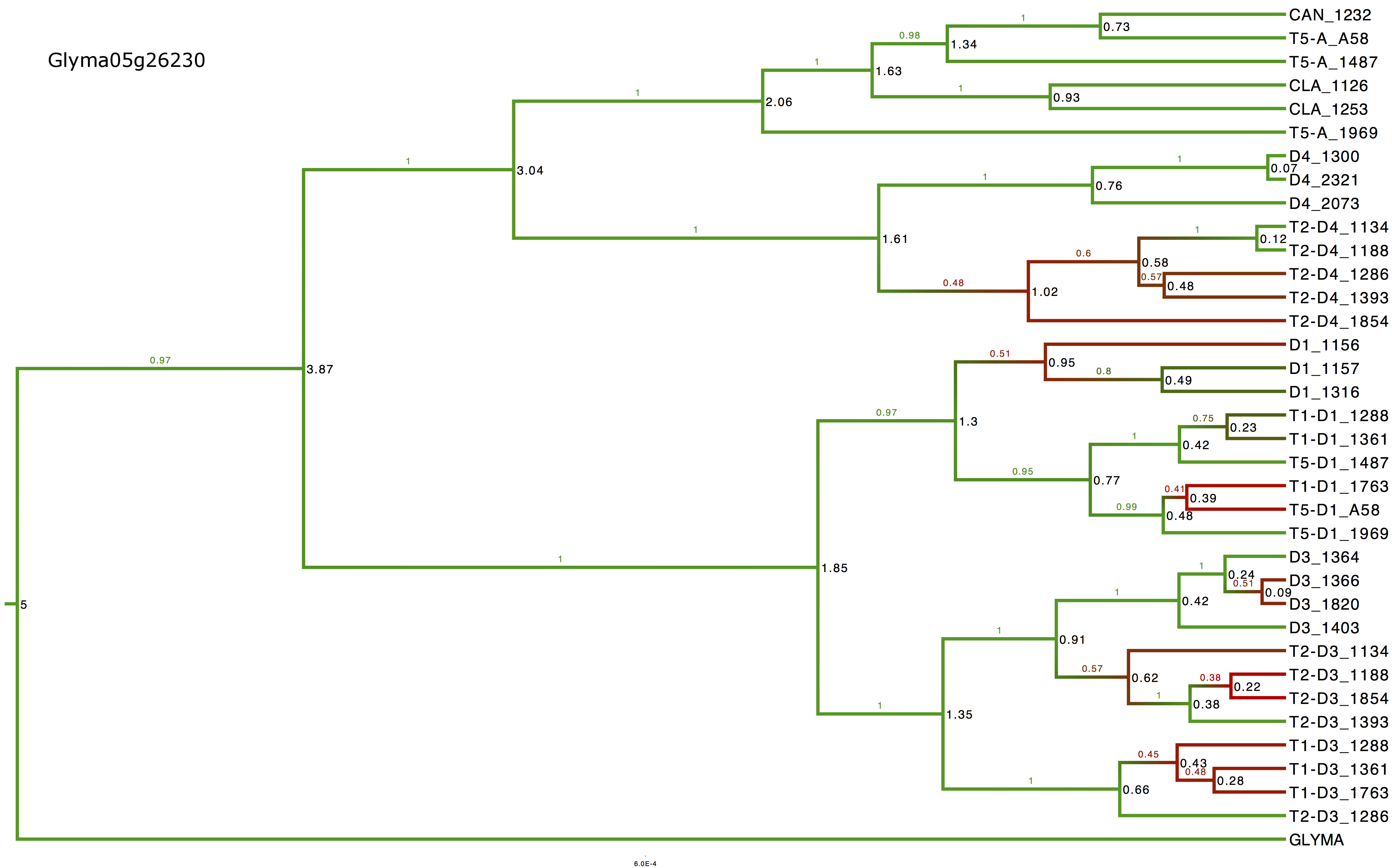

Supplement: Figures S41--S67 — The color of the tree branch represents the posterior probability value (also with the same color). Genes are in the following order: Glyma01g35620 (Figure S41), Glyma02g11580 (Figure S42), Glyma03g29330 (Figure S43), Glyma03g36630 (Figure S44), Glyma04g39670 (Figure S45), Glyma05g05750 (Figure S46), Glyma05g09310 (Figure S47), Glyma05g26230 (Figure S48), Glyma05g37840 (Figure S49), Glyma06g18640 (Figure S50), Glyma07g03370 (Figure S51), Glyma07g17180 (Figure S52), Glyma10g42100 (Figure S53), Glyma11g13880 (Figure S54), Glyma11g33720 (Figure S55), Glyma12g04150 (Figure S56), Glyma12g12230 (Figure S57), Glyma13g17820 (Figure S58), Glyma14g03500 (Figure S59), Glyma16g00410 (Figure S60), Glyma16g01980 (Figure S61), Glyma16g04940 (Figure S62), Glyma18g04080 (Figure S63), Glyma19g03390 (Figure S64), Glyma19g32940 (Figure S65), Glyma20g24930 (Figure S66), Glyma20g32930 (Figure S67). [file peerj-02-391-s003.zip › Suplementary_Figures_S41-S67/Suplementary_Figure_48.png]

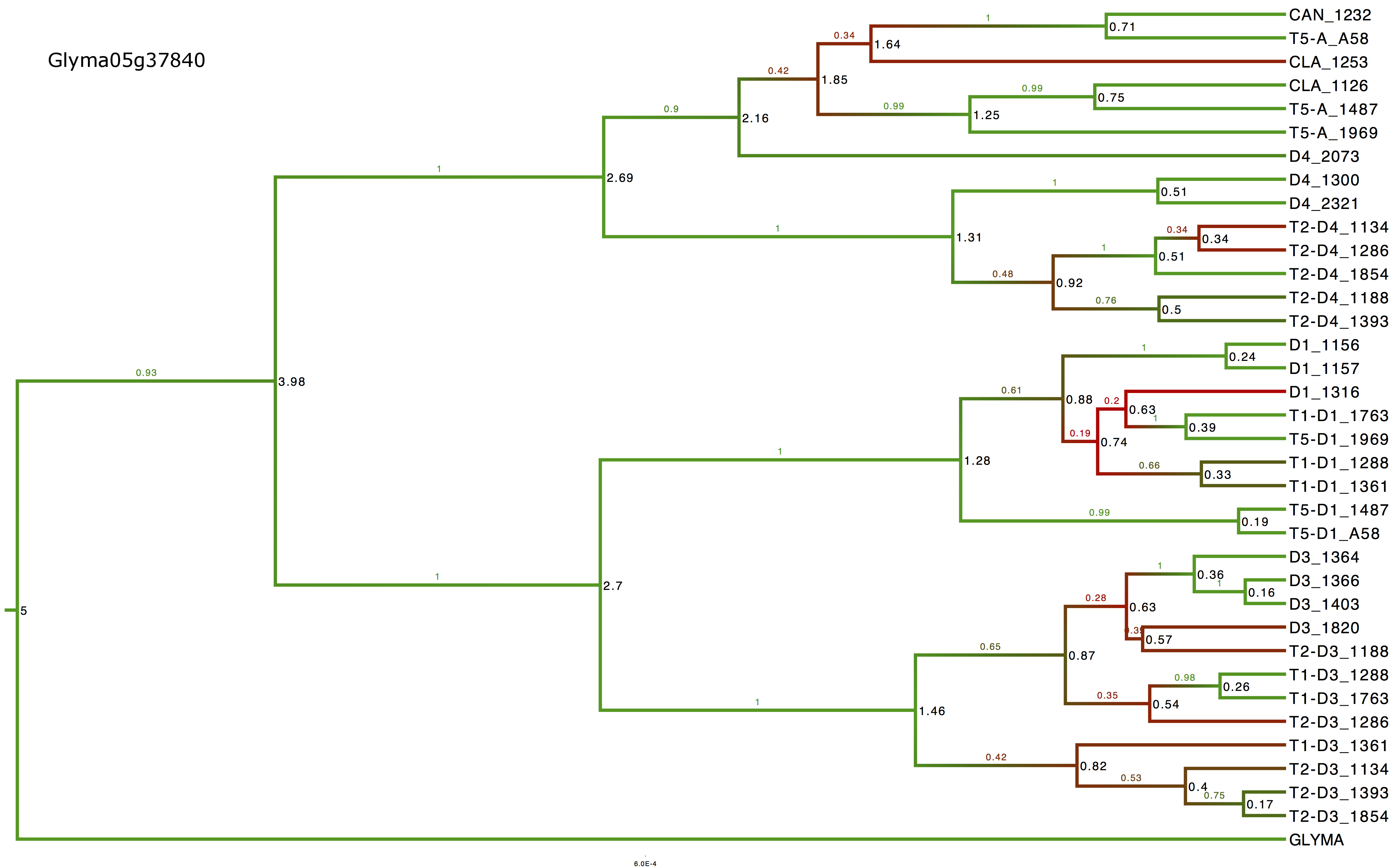

Supplement: Figures S41--S67 — The color of the tree branch represents the posterior probability value (also with the same color). Genes are in the following order: Glyma01g35620 (Figure S41), Glyma02g11580 (Figure S42), Glyma03g29330 (Figure S43), Glyma03g36630 (Figure S44), Glyma04g39670 (Figure S45), Glyma05g05750 (Figure S46), Glyma05g09310 (Figure S47), Glyma05g26230 (Figure S48), Glyma05g37840 (Figure S49), Glyma06g18640 (Figure S50), Glyma07g03370 (Figure S51), Glyma07g17180 (Figure S52), Glyma10g42100 (Figure S53), Glyma11g13880 (Figure S54), Glyma11g33720 (Figure S55), Glyma12g04150 (Figure S56), Glyma12g12230 (Figure S57), Glyma13g17820 (Figure S58), Glyma14g03500 (Figure S59), Glyma16g00410 (Figure S60), Glyma16g01980 (Figure S61), Glyma16g04940 (Figure S62), Glyma18g04080 (Figure S63), Glyma19g03390 (Figure S64), Glyma19g32940 (Figure S65), Glyma20g24930 (Figure S66), Glyma20g32930 (Figure S67). [file peerj-02-391-s003.zip › Suplementary_Figures_S41-S67/Suplementary_Figure_49.png]

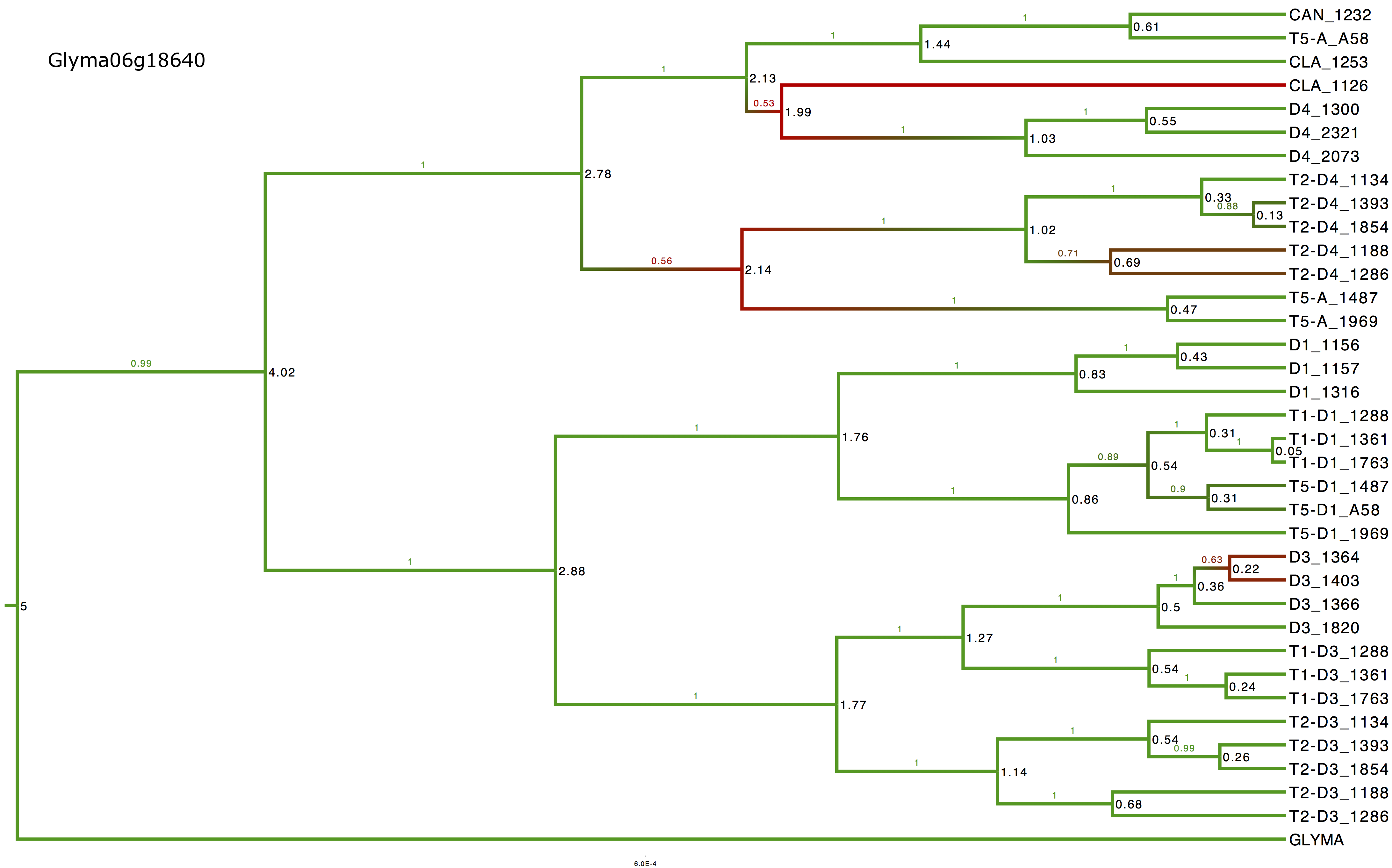

Supplement: Figures S41--S67 — The color of the tree branch represents the posterior probability value (also with the same color). Genes are in the following order: Glyma01g35620 (Figure S41), Glyma02g11580 (Figure S42), Glyma03g29330 (Figure S43), Glyma03g36630 (Figure S44), Glyma04g39670 (Figure S45), Glyma05g05750 (Figure S46), Glyma05g09310 (Figure S47), Glyma05g26230 (Figure S48), Glyma05g37840 (Figure S49), Glyma06g18640 (Figure S50), Glyma07g03370 (Figure S51), Glyma07g17180 (Figure S52), Glyma10g42100 (Figure S53), Glyma11g13880 (Figure S54), Glyma11g33720 (Figure S55), Glyma12g04150 (Figure S56), Glyma12g12230 (Figure S57), Glyma13g17820 (Figure S58), Glyma14g03500 (Figure S59), Glyma16g00410 (Figure S60), Glyma16g01980 (Figure S61), Glyma16g04940 (Figure S62), Glyma18g04080 (Figure S63), Glyma19g03390 (Figure S64), Glyma19g32940 (Figure S65), Glyma20g24930 (Figure S66), Glyma20g32930 (Figure S67). [file peerj-02-391-s003.zip › Suplementary_Figures_S41-S67/Suplementary_Figure_50.png]

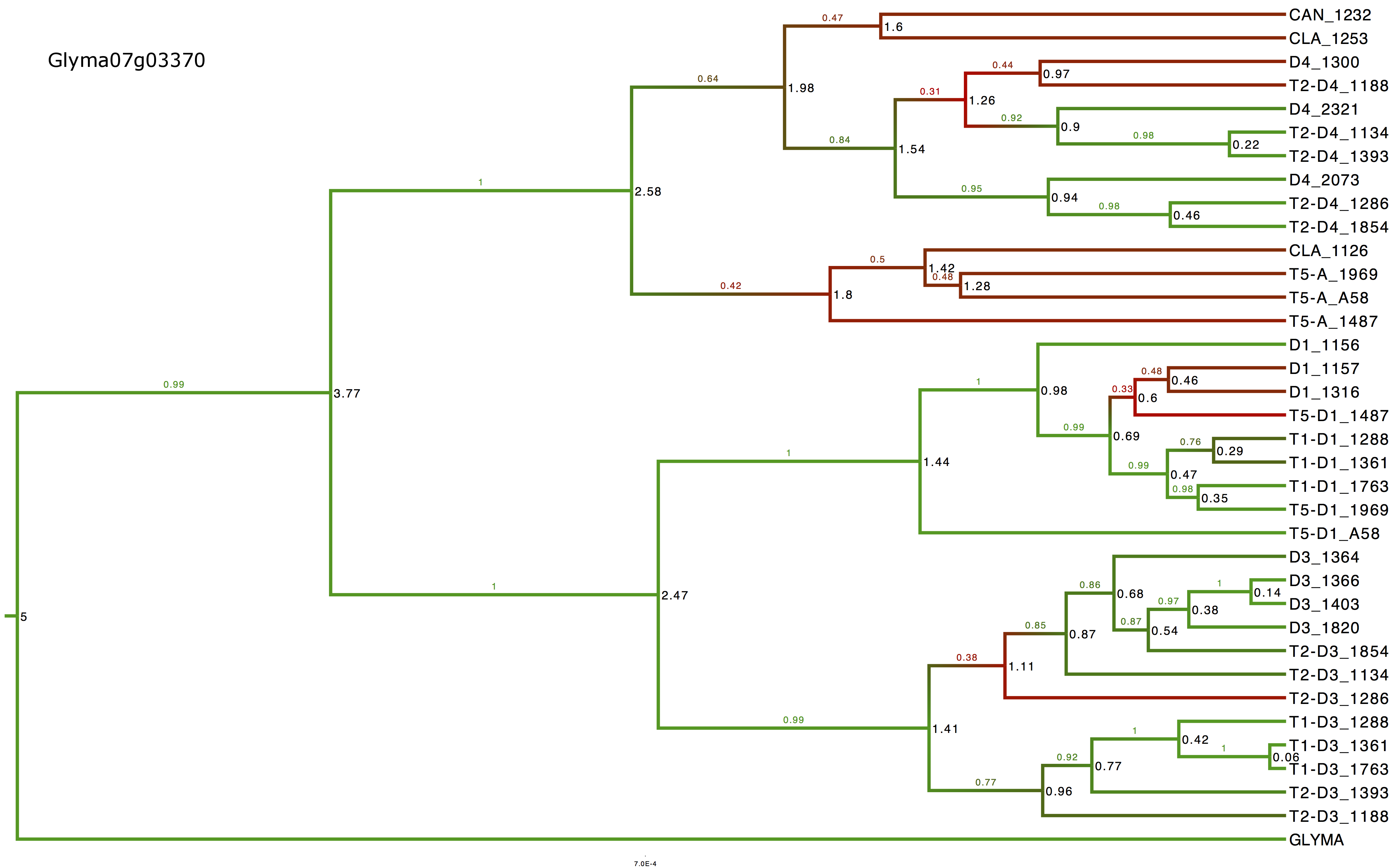

Supplement: Figures S41--S67 — The color of the tree branch represents the posterior probability value (also with the same color). Genes are in the following order: Glyma01g35620 (Figure S41), Glyma02g11580 (Figure S42), Glyma03g29330 (Figure S43), Glyma03g36630 (Figure S44), Glyma04g39670 (Figure S45), Glyma05g05750 (Figure S46), Glyma05g09310 (Figure S47), Glyma05g26230 (Figure S48), Glyma05g37840 (Figure S49), Glyma06g18640 (Figure S50), Glyma07g03370 (Figure S51), Glyma07g17180 (Figure S52), Glyma10g42100 (Figure S53), Glyma11g13880 (Figure S54), Glyma11g33720 (Figure S55), Glyma12g04150 (Figure S56), Glyma12g12230 (Figure S57), Glyma13g17820 (Figure S58), Glyma14g03500 (Figure S59), Glyma16g00410 (Figure S60), Glyma16g01980 (Figure S61), Glyma16g04940 (Figure S62), Glyma18g04080 (Figure S63), Glyma19g03390 (Figure S64), Glyma19g32940 (Figure S65), Glyma20g24930 (Figure S66), Glyma20g32930 (Figure S67). [file peerj-02-391-s003.zip › Suplementary_Figures_S41-S67/Suplementary_Figure_51.png]

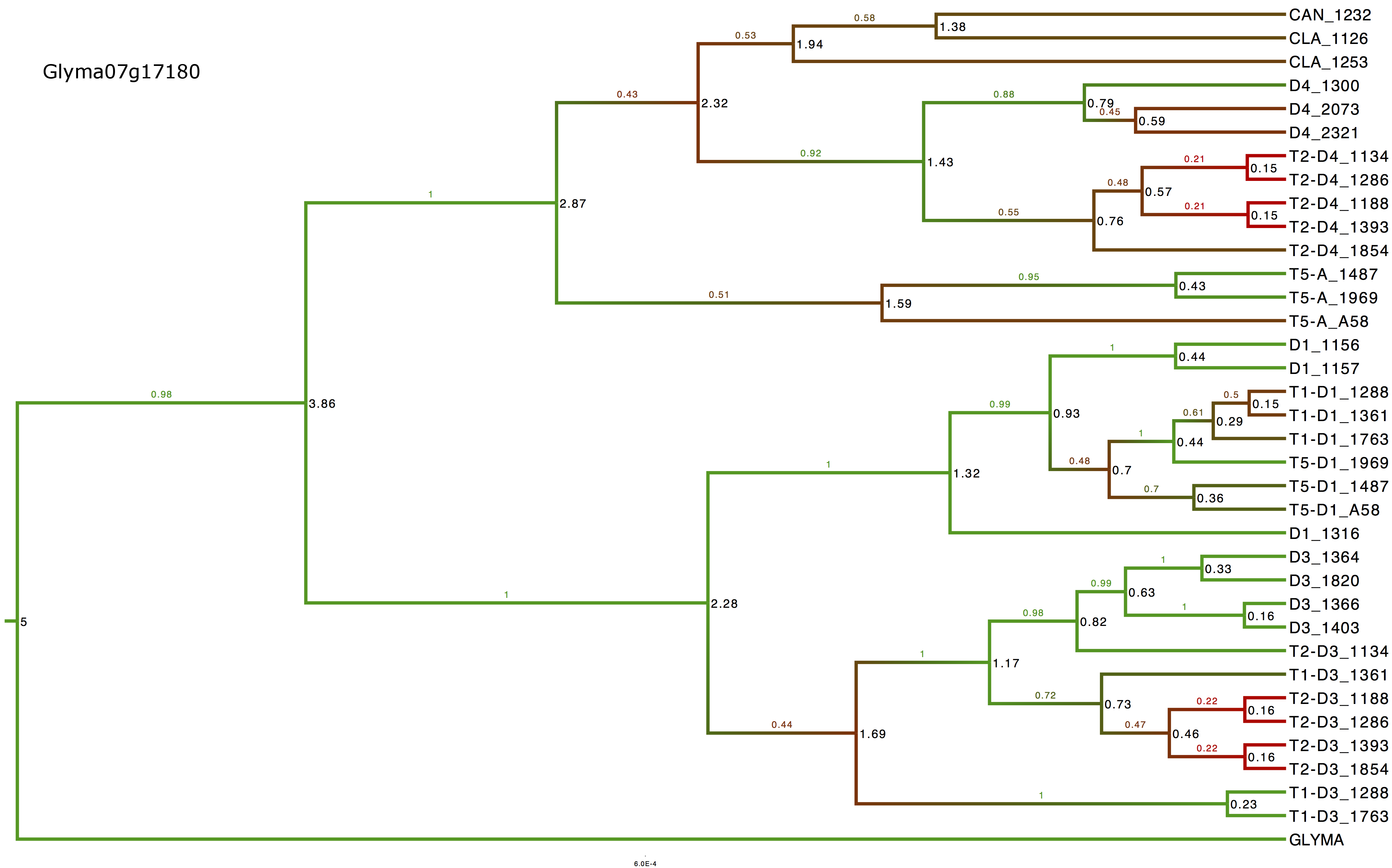

Supplement: Figures S41--S67 — The color of the tree branch represents the posterior probability value (also with the same color). Genes are in the following order: Glyma01g35620 (Figure S41), Glyma02g11580 (Figure S42), Glyma03g29330 (Figure S43), Glyma03g36630 (Figure S44), Glyma04g39670 (Figure S45), Glyma05g05750 (Figure S46), Glyma05g09310 (Figure S47), Glyma05g26230 (Figure S48), Glyma05g37840 (Figure S49), Glyma06g18640 (Figure S50), Glyma07g03370 (Figure S51), Glyma07g17180 (Figure S52), Glyma10g42100 (Figure S53), Glyma11g13880 (Figure S54), Glyma11g33720 (Figure S55), Glyma12g04150 (Figure S56), Glyma12g12230 (Figure S57), Glyma13g17820 (Figure S58), Glyma14g03500 (Figure S59), Glyma16g00410 (Figure S60), Glyma16g01980 (Figure S61), Glyma16g04940 (Figure S62), Glyma18g04080 (Figure S63), Glyma19g03390 (Figure S64), Glyma19g32940 (Figure S65), Glyma20g24930 (Figure S66), Glyma20g32930 (Figure S67). [file peerj-02-391-s003.zip › Suplementary_Figures_S41-S67/Suplementary_Figure_52.png]

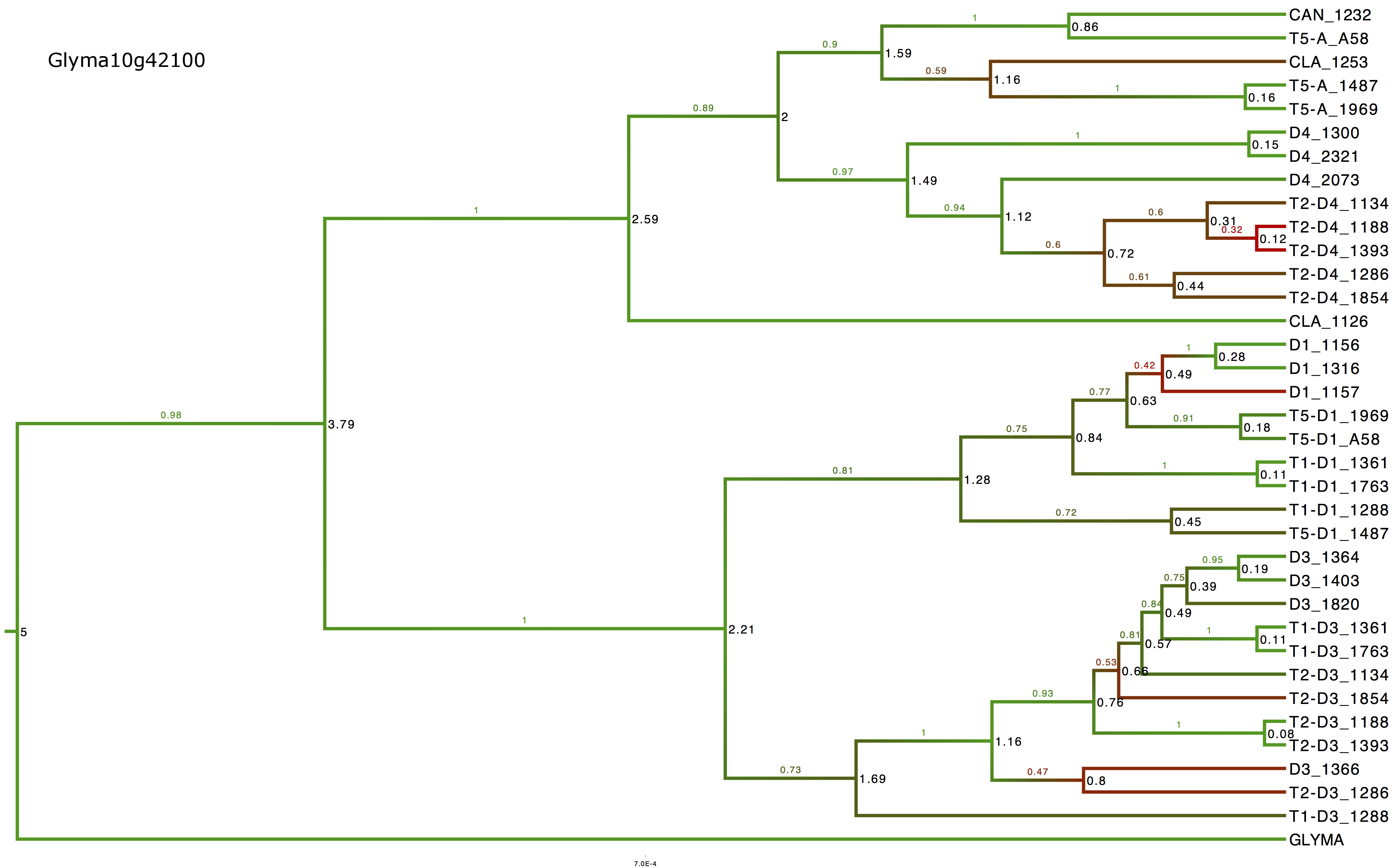

Supplement: Figures S41--S67 — The color of the tree branch represents the posterior probability value (also with the same color). Genes are in the following order: Glyma01g35620 (Figure S41), Glyma02g11580 (Figure S42), Glyma03g29330 (Figure S43), Glyma03g36630 (Figure S44), Glyma04g39670 (Figure S45), Glyma05g05750 (Figure S46), Glyma05g09310 (Figure S47), Glyma05g26230 (Figure S48), Glyma05g37840 (Figure S49), Glyma06g18640 (Figure S50), Glyma07g03370 (Figure S51), Glyma07g17180 (Figure S52), Glyma10g42100 (Figure S53), Glyma11g13880 (Figure S54), Glyma11g33720 (Figure S55), Glyma12g04150 (Figure S56), Glyma12g12230 (Figure S57), Glyma13g17820 (Figure S58), Glyma14g03500 (Figure S59), Glyma16g00410 (Figure S60), Glyma16g01980 (Figure S61), Glyma16g04940 (Figure S62), Glyma18g04080 (Figure S63), Glyma19g03390 (Figure S64), Glyma19g32940 (Figure S65), Glyma20g24930 (Figure S66), Glyma20g32930 (Figure S67). [file peerj-02-391-s003.zip › Suplementary_Figures_S41-S67/Suplementary_Figure_53.png]

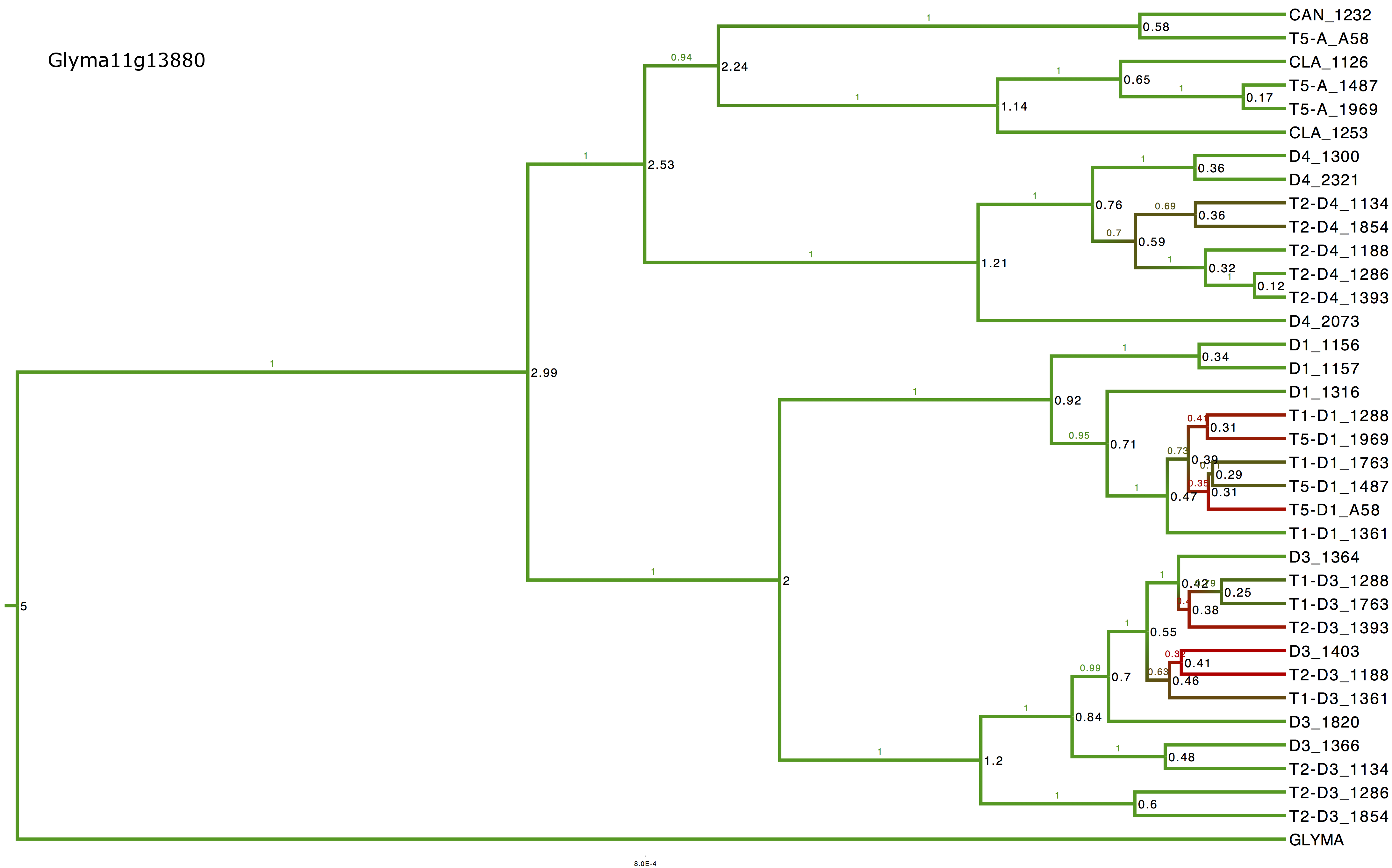

Supplement: Figures S41--S67 — The color of the tree branch represents the posterior probability value (also with the same color). Genes are in the following order: Glyma01g35620 (Figure S41), Glyma02g11580 (Figure S42), Glyma03g29330 (Figure S43), Glyma03g36630 (Figure S44), Glyma04g39670 (Figure S45), Glyma05g05750 (Figure S46), Glyma05g09310 (Figure S47), Glyma05g26230 (Figure S48), Glyma05g37840 (Figure S49), Glyma06g18640 (Figure S50), Glyma07g03370 (Figure S51), Glyma07g17180 (Figure S52), Glyma10g42100 (Figure S53), Glyma11g13880 (Figure S54), Glyma11g33720 (Figure S55), Glyma12g04150 (Figure S56), Glyma12g12230 (Figure S57), Glyma13g17820 (Figure S58), Glyma14g03500 (Figure S59), Glyma16g00410 (Figure S60), Glyma16g01980 (Figure S61), Glyma16g04940 (Figure S62), Glyma18g04080 (Figure S63), Glyma19g03390 (Figure S64), Glyma19g32940 (Figure S65), Glyma20g24930 (Figure S66), Glyma20g32930 (Figure S67). [file peerj-02-391-s003.zip › Suplementary_Figures_S41-S67/Suplementary_Figure_54.png]

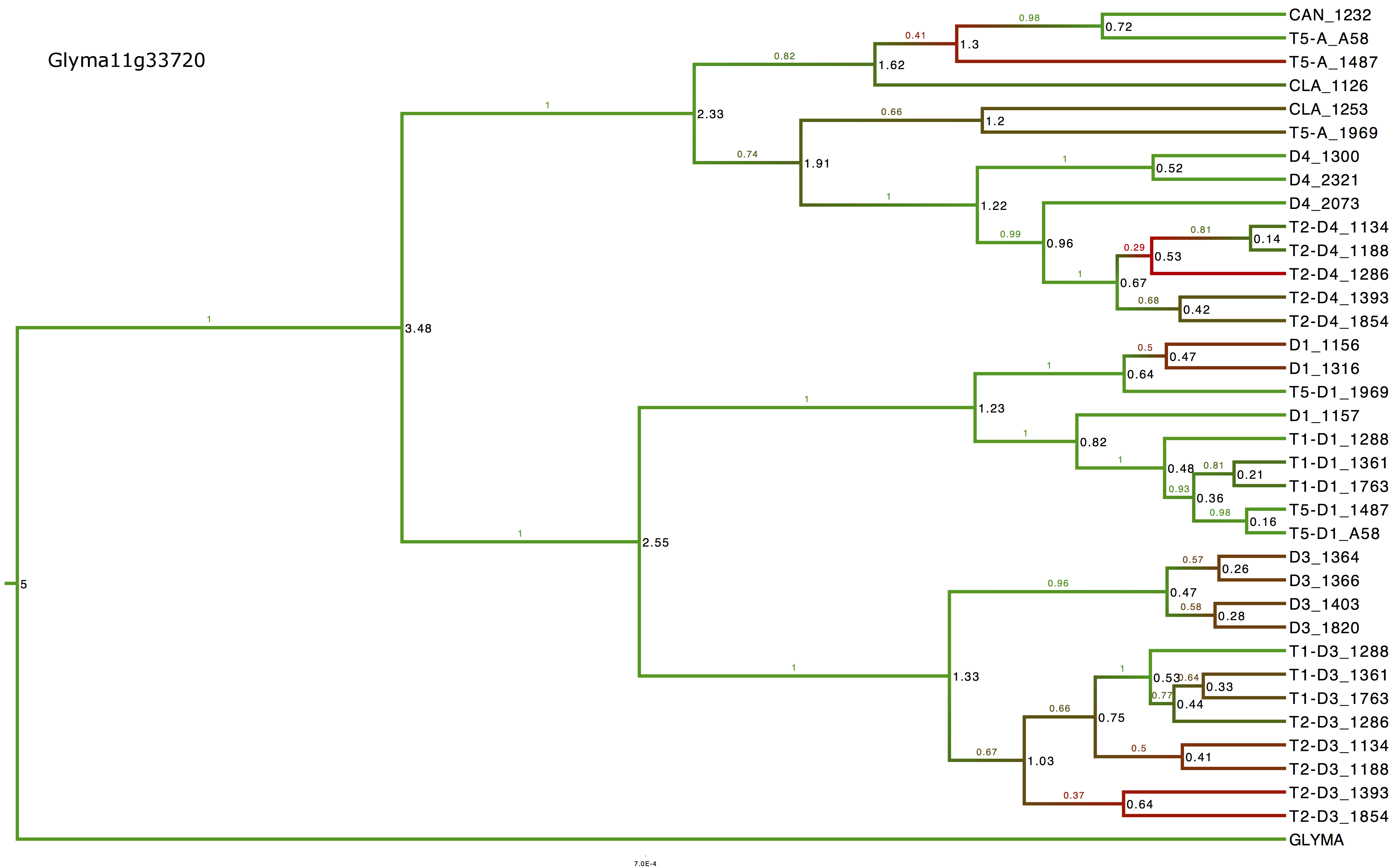

Supplement: Figures S41--S67 — The color of the tree branch represents the posterior probability value (also with the same color). Genes are in the following order: Glyma01g35620 (Figure S41), Glyma02g11580 (Figure S42), Glyma03g29330 (Figure S43), Glyma03g36630 (Figure S44), Glyma04g39670 (Figure S45), Glyma05g05750 (Figure S46), Glyma05g09310 (Figure S47), Glyma05g26230 (Figure S48), Glyma05g37840 (Figure S49), Glyma06g18640 (Figure S50), Glyma07g03370 (Figure S51), Glyma07g17180 (Figure S52), Glyma10g42100 (Figure S53), Glyma11g13880 (Figure S54), Glyma11g33720 (Figure S55), Glyma12g04150 (Figure S56), Glyma12g12230 (Figure S57), Glyma13g17820 (Figure S58), Glyma14g03500 (Figure S59), Glyma16g00410 (Figure S60), Glyma16g01980 (Figure S61), Glyma16g04940 (Figure S62), Glyma18g04080 (Figure S63), Glyma19g03390 (Figure S64), Glyma19g32940 (Figure S65), Glyma20g24930 (Figure S66), Glyma20g32930 (Figure S67). [file peerj-02-391-s003.zip › Suplementary_Figures_S41-S67/Suplementary_Figure_55.png]

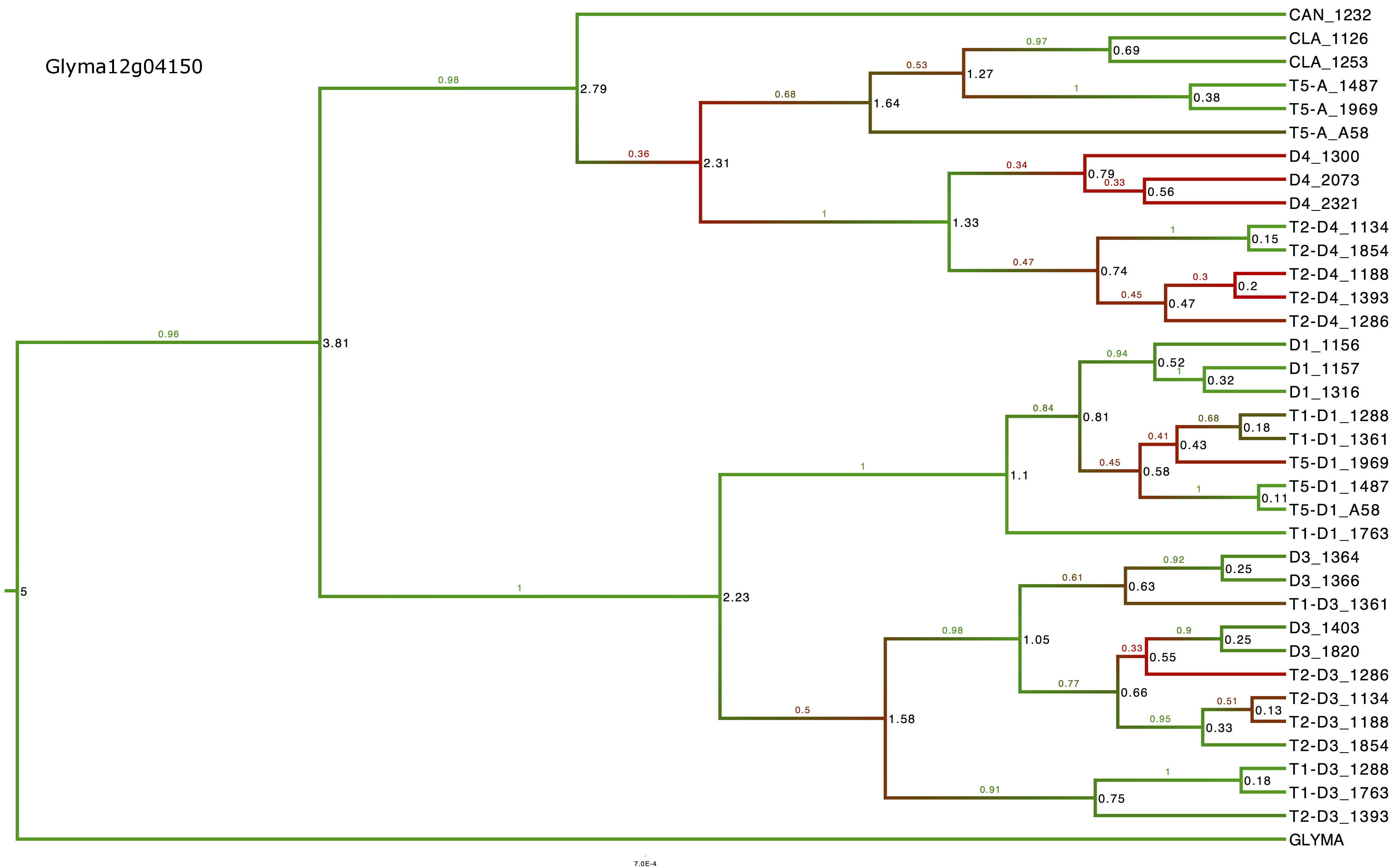

Supplement: Figures S41--S67 — The color of the tree branch represents the posterior probability value (also with the same color). Genes are in the following order: Glyma01g35620 (Figure S41), Glyma02g11580 (Figure S42), Glyma03g29330 (Figure S43), Glyma03g36630 (Figure S44), Glyma04g39670 (Figure S45), Glyma05g05750 (Figure S46), Glyma05g09310 (Figure S47), Glyma05g26230 (Figure S48), Glyma05g37840 (Figure S49), Glyma06g18640 (Figure S50), Glyma07g03370 (Figure S51), Glyma07g17180 (Figure S52), Glyma10g42100 (Figure S53), Glyma11g13880 (Figure S54), Glyma11g33720 (Figure S55), Glyma12g04150 (Figure S56), Glyma12g12230 (Figure S57), Glyma13g17820 (Figure S58), Glyma14g03500 (Figure S59), Glyma16g00410 (Figure S60), Glyma16g01980 (Figure S61), Glyma16g04940 (Figure S62), Glyma18g04080 (Figure S63), Glyma19g03390 (Figure S64), Glyma19g32940 (Figure S65), Glyma20g24930 (Figure S66), Glyma20g32930 (Figure S67). [file peerj-02-391-s003.zip › Suplementary_Figures_S41-S67/Suplementary_Figure_56.png]

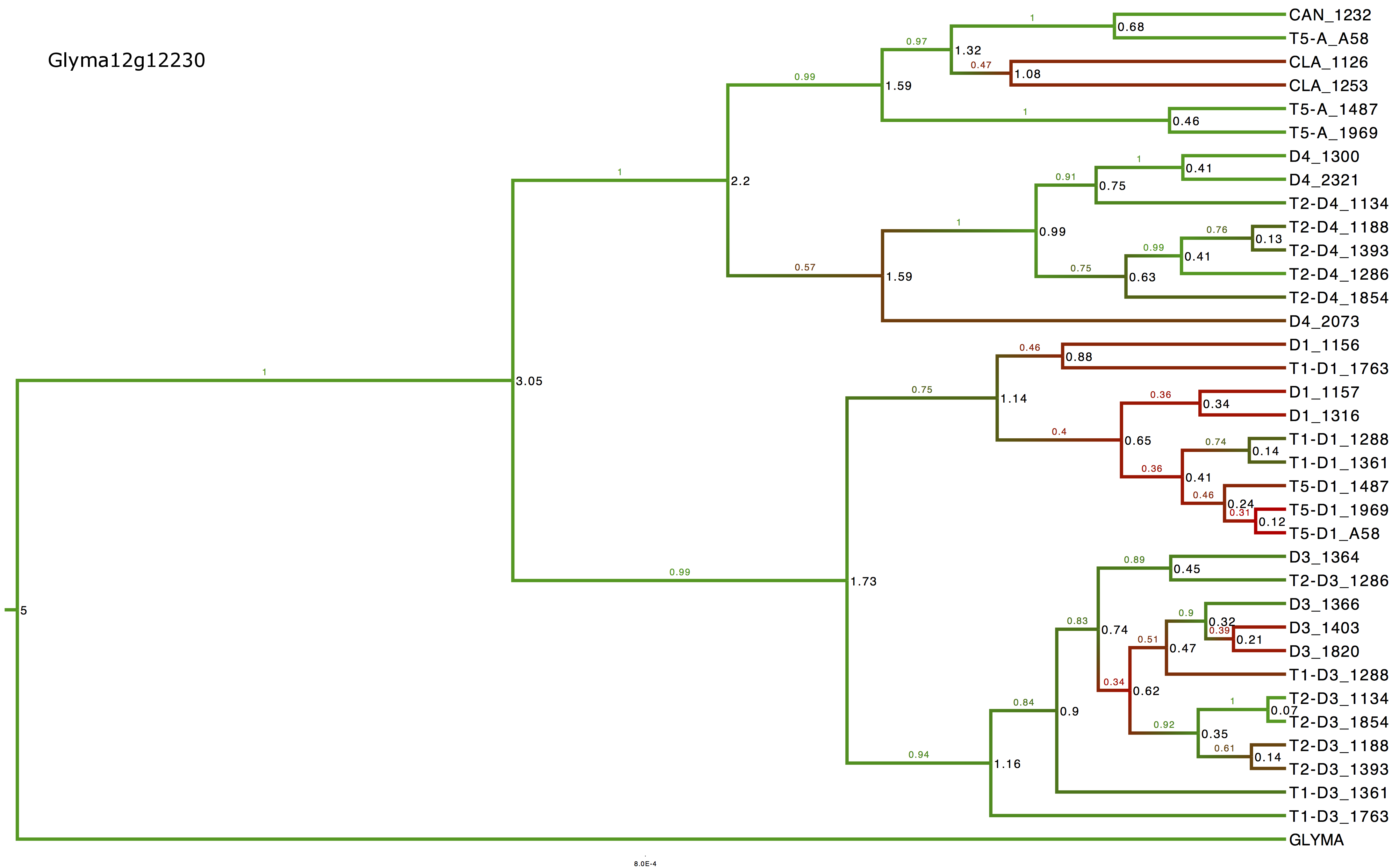

Supplement: Figures S41--S67 — The color of the tree branch represents the posterior probability value (also with the same color). Genes are in the following order: Glyma01g35620 (Figure S41), Glyma02g11580 (Figure S42), Glyma03g29330 (Figure S43), Glyma03g36630 (Figure S44), Glyma04g39670 (Figure S45), Glyma05g05750 (Figure S46), Glyma05g09310 (Figure S47), Glyma05g26230 (Figure S48), Glyma05g37840 (Figure S49), Glyma06g18640 (Figure S50), Glyma07g03370 (Figure S51), Glyma07g17180 (Figure S52), Glyma10g42100 (Figure S53), Glyma11g13880 (Figure S54), Glyma11g33720 (Figure S55), Glyma12g04150 (Figure S56), Glyma12g12230 (Figure S57), Glyma13g17820 (Figure S58), Glyma14g03500 (Figure S59), Glyma16g00410 (Figure S60), Glyma16g01980 (Figure S61), Glyma16g04940 (Figure S62), Glyma18g04080 (Figure S63), Glyma19g03390 (Figure S64), Glyma19g32940 (Figure S65), Glyma20g24930 (Figure S66), Glyma20g32930 (Figure S67). [file peerj-02-391-s003.zip › Suplementary_Figures_S41-S67/Suplementary_Figure_57.png]

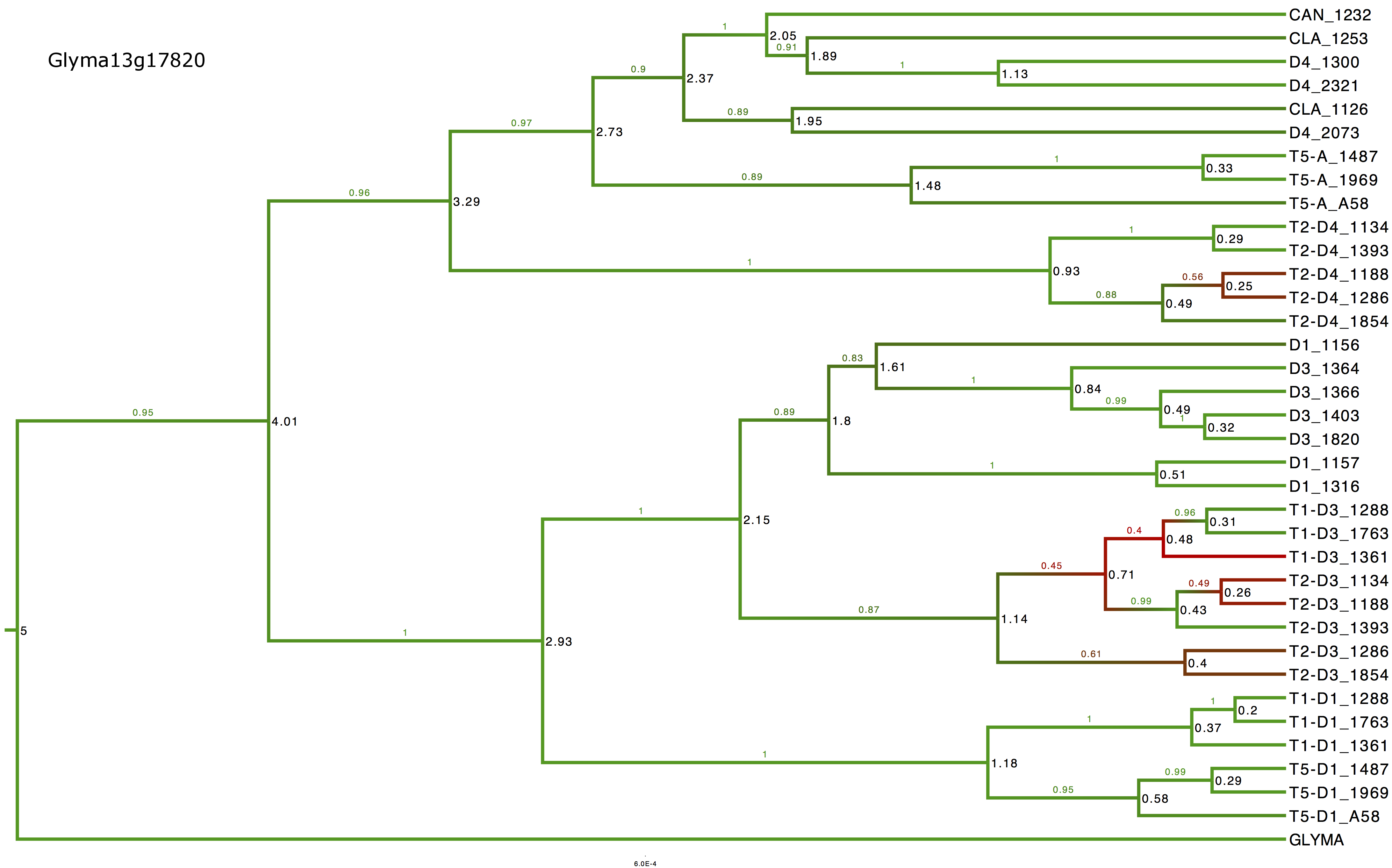

Supplement: Figures S41--S67 — The color of the tree branch represents the posterior probability value (also with the same color). Genes are in the following order: Glyma01g35620 (Figure S41), Glyma02g11580 (Figure S42), Glyma03g29330 (Figure S43), Glyma03g36630 (Figure S44), Glyma04g39670 (Figure S45), Glyma05g05750 (Figure S46), Glyma05g09310 (Figure S47), Glyma05g26230 (Figure S48), Glyma05g37840 (Figure S49), Glyma06g18640 (Figure S50), Glyma07g03370 (Figure S51), Glyma07g17180 (Figure S52), Glyma10g42100 (Figure S53), Glyma11g13880 (Figure S54), Glyma11g33720 (Figure S55), Glyma12g04150 (Figure S56), Glyma12g12230 (Figure S57), Glyma13g17820 (Figure S58), Glyma14g03500 (Figure S59), Glyma16g00410 (Figure S60), Glyma16g01980 (Figure S61), Glyma16g04940 (Figure S62), Glyma18g04080 (Figure S63), Glyma19g03390 (Figure S64), Glyma19g32940 (Figure S65), Glyma20g24930 (Figure S66), Glyma20g32930 (Figure S67). [file peerj-02-391-s003.zip › Suplementary_Figures_S41-S67/Suplementary_Figure_58.png]

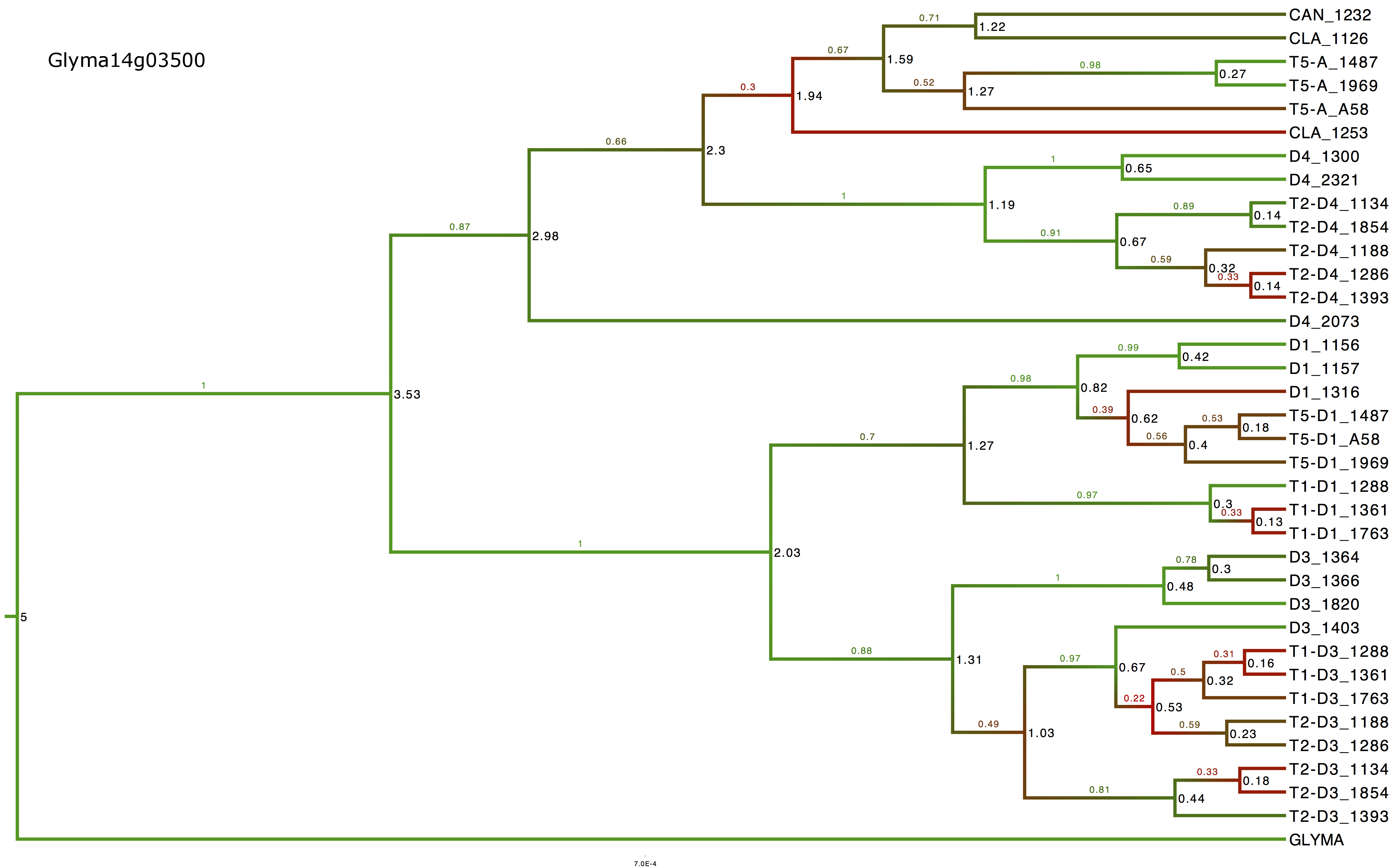

Supplement: Figures S41--S67 — The color of the tree branch represents the posterior probability value (also with the same color). Genes are in the following order: Glyma01g35620 (Figure S41), Glyma02g11580 (Figure S42), Glyma03g29330 (Figure S43), Glyma03g36630 (Figure S44), Glyma04g39670 (Figure S45), Glyma05g05750 (Figure S46), Glyma05g09310 (Figure S47), Glyma05g26230 (Figure S48), Glyma05g37840 (Figure S49), Glyma06g18640 (Figure S50), Glyma07g03370 (Figure S51), Glyma07g17180 (Figure S52), Glyma10g42100 (Figure S53), Glyma11g13880 (Figure S54), Glyma11g33720 (Figure S55), Glyma12g04150 (Figure S56), Glyma12g12230 (Figure S57), Glyma13g17820 (Figure S58), Glyma14g03500 (Figure S59), Glyma16g00410 (Figure S60), Glyma16g01980 (Figure S61), Glyma16g04940 (Figure S62), Glyma18g04080 (Figure S63), Glyma19g03390 (Figure S64), Glyma19g32940 (Figure S65), Glyma20g24930 (Figure S66), Glyma20g32930 (Figure S67). [file peerj-02-391-s003.zip › Suplementary_Figures_S41-S67/Suplementary_Figure_59.png]

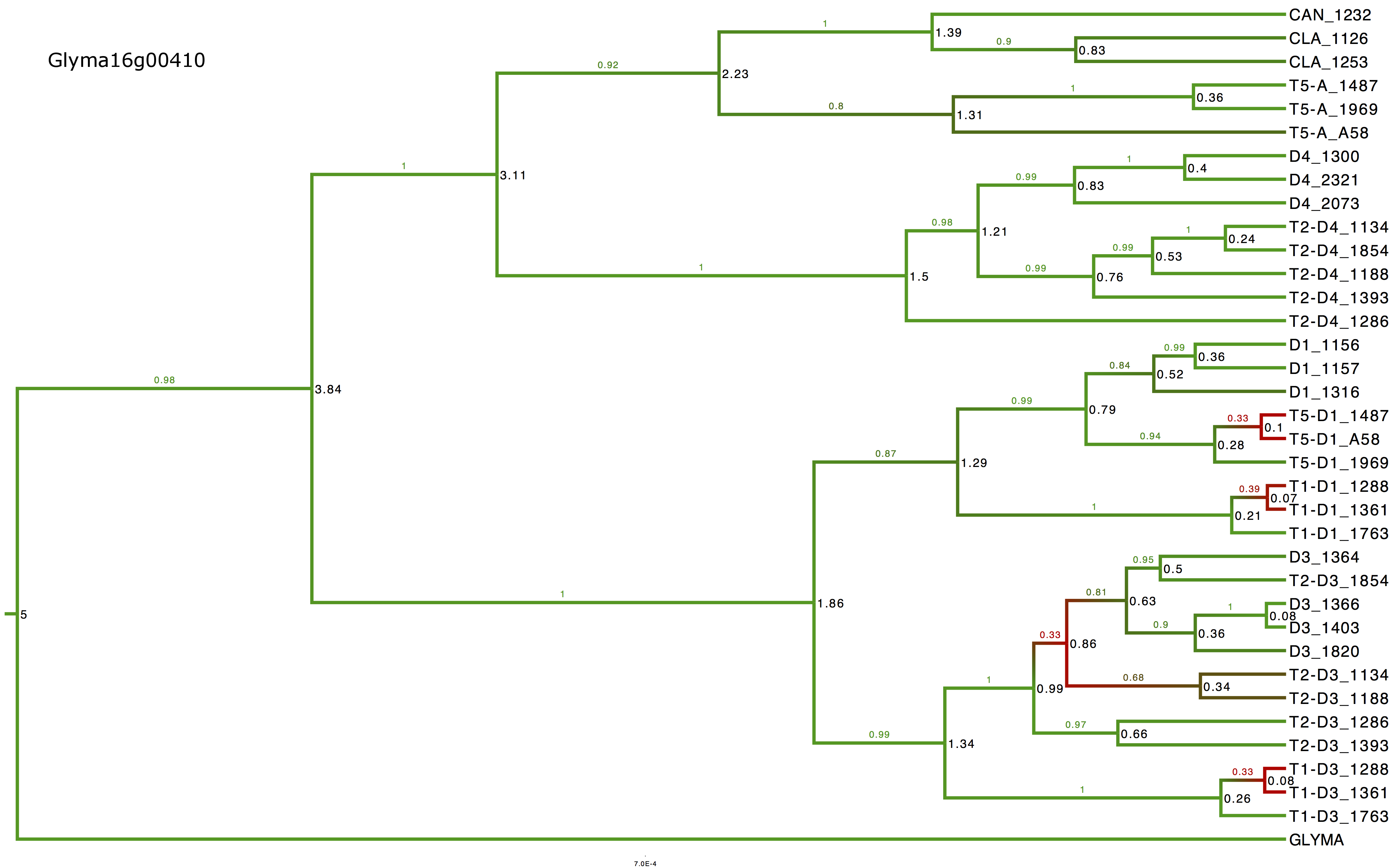

Supplement: Figures S41--S67 — The color of the tree branch represents the posterior probability value (also with the same color). Genes are in the following order: Glyma01g35620 (Figure S41), Glyma02g11580 (Figure S42), Glyma03g29330 (Figure S43), Glyma03g36630 (Figure S44), Glyma04g39670 (Figure S45), Glyma05g05750 (Figure S46), Glyma05g09310 (Figure S47), Glyma05g26230 (Figure S48), Glyma05g37840 (Figure S49), Glyma06g18640 (Figure S50), Glyma07g03370 (Figure S51), Glyma07g17180 (Figure S52), Glyma10g42100 (Figure S53), Glyma11g13880 (Figure S54), Glyma11g33720 (Figure S55), Glyma12g04150 (Figure S56), Glyma12g12230 (Figure S57), Glyma13g17820 (Figure S58), Glyma14g03500 (Figure S59), Glyma16g00410 (Figure S60), Glyma16g01980 (Figure S61), Glyma16g04940 (Figure S62), Glyma18g04080 (Figure S63), Glyma19g03390 (Figure S64), Glyma19g32940 (Figure S65), Glyma20g24930 (Figure S66), Glyma20g32930 (Figure S67). [file peerj-02-391-s003.zip › Suplementary_Figures_S41-S67/Suplementary_Figure_60.png]

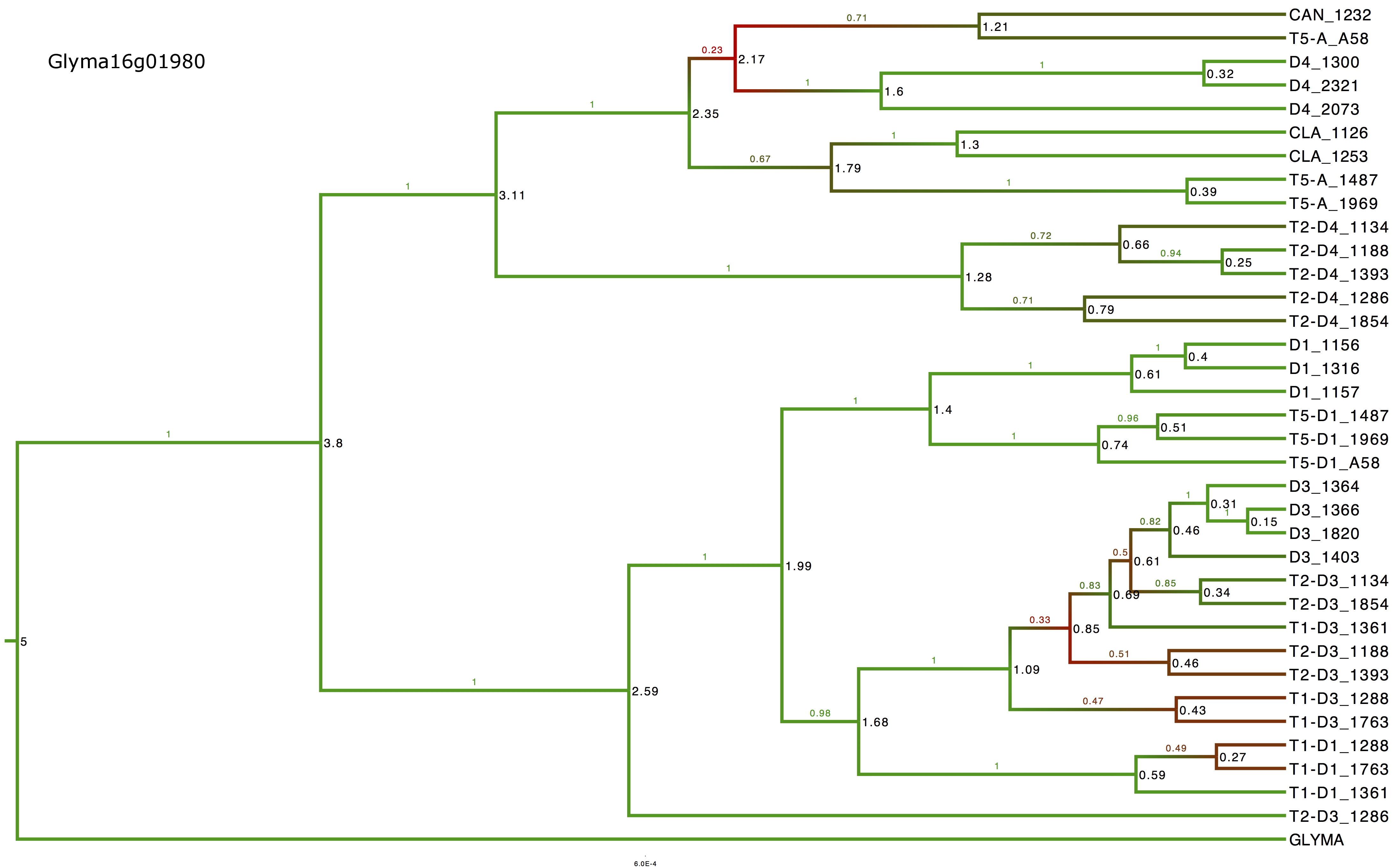

Supplement: Figures S41--S67 — The color of the tree branch represents the posterior probability value (also with the same color). Genes are in the following order: Glyma01g35620 (Figure S41), Glyma02g11580 (Figure S42), Glyma03g29330 (Figure S43), Glyma03g36630 (Figure S44), Glyma04g39670 (Figure S45), Glyma05g05750 (Figure S46), Glyma05g09310 (Figure S47), Glyma05g26230 (Figure S48), Glyma05g37840 (Figure S49), Glyma06g18640 (Figure S50), Glyma07g03370 (Figure S51), Glyma07g17180 (Figure S52), Glyma10g42100 (Figure S53), Glyma11g13880 (Figure S54), Glyma11g33720 (Figure S55), Glyma12g04150 (Figure S56), Glyma12g12230 (Figure S57), Glyma13g17820 (Figure S58), Glyma14g03500 (Figure S59), Glyma16g00410 (Figure S60), Glyma16g01980 (Figure S61), Glyma16g04940 (Figure S62), Glyma18g04080 (Figure S63), Glyma19g03390 (Figure S64), Glyma19g32940 (Figure S65), Glyma20g24930 (Figure S66), Glyma20g32930 (Figure S67). [file peerj-02-391-s003.zip › Suplementary_Figures_S41-S67/Suplementary_Figure_61.png]

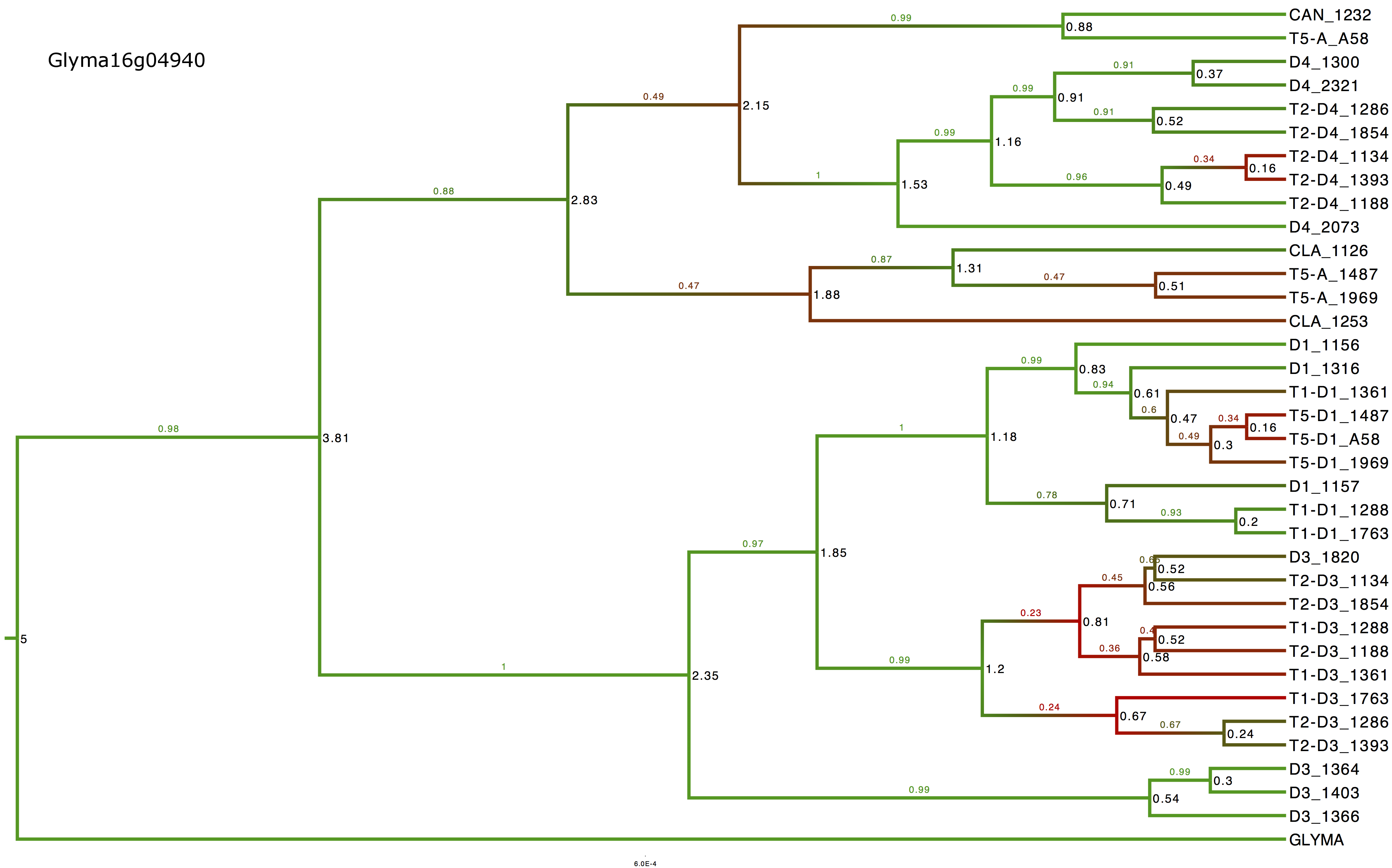

Supplement: Figures S41--S67 — The color of the tree branch represents the posterior probability value (also with the same color). Genes are in the following order: Glyma01g35620 (Figure S41), Glyma02g11580 (Figure S42), Glyma03g29330 (Figure S43), Glyma03g36630 (Figure S44), Glyma04g39670 (Figure S45), Glyma05g05750 (Figure S46), Glyma05g09310 (Figure S47), Glyma05g26230 (Figure S48), Glyma05g37840 (Figure S49), Glyma06g18640 (Figure S50), Glyma07g03370 (Figure S51), Glyma07g17180 (Figure S52), Glyma10g42100 (Figure S53), Glyma11g13880 (Figure S54), Glyma11g33720 (Figure S55), Glyma12g04150 (Figure S56), Glyma12g12230 (Figure S57), Glyma13g17820 (Figure S58), Glyma14g03500 (Figure S59), Glyma16g00410 (Figure S60), Glyma16g01980 (Figure S61), Glyma16g04940 (Figure S62), Glyma18g04080 (Figure S63), Glyma19g03390 (Figure S64), Glyma19g32940 (Figure S65), Glyma20g24930 (Figure S66), Glyma20g32930 (Figure S67). [file peerj-02-391-s003.zip › Suplementary_Figures_S41-S67/Suplementary_Figure_62.png]

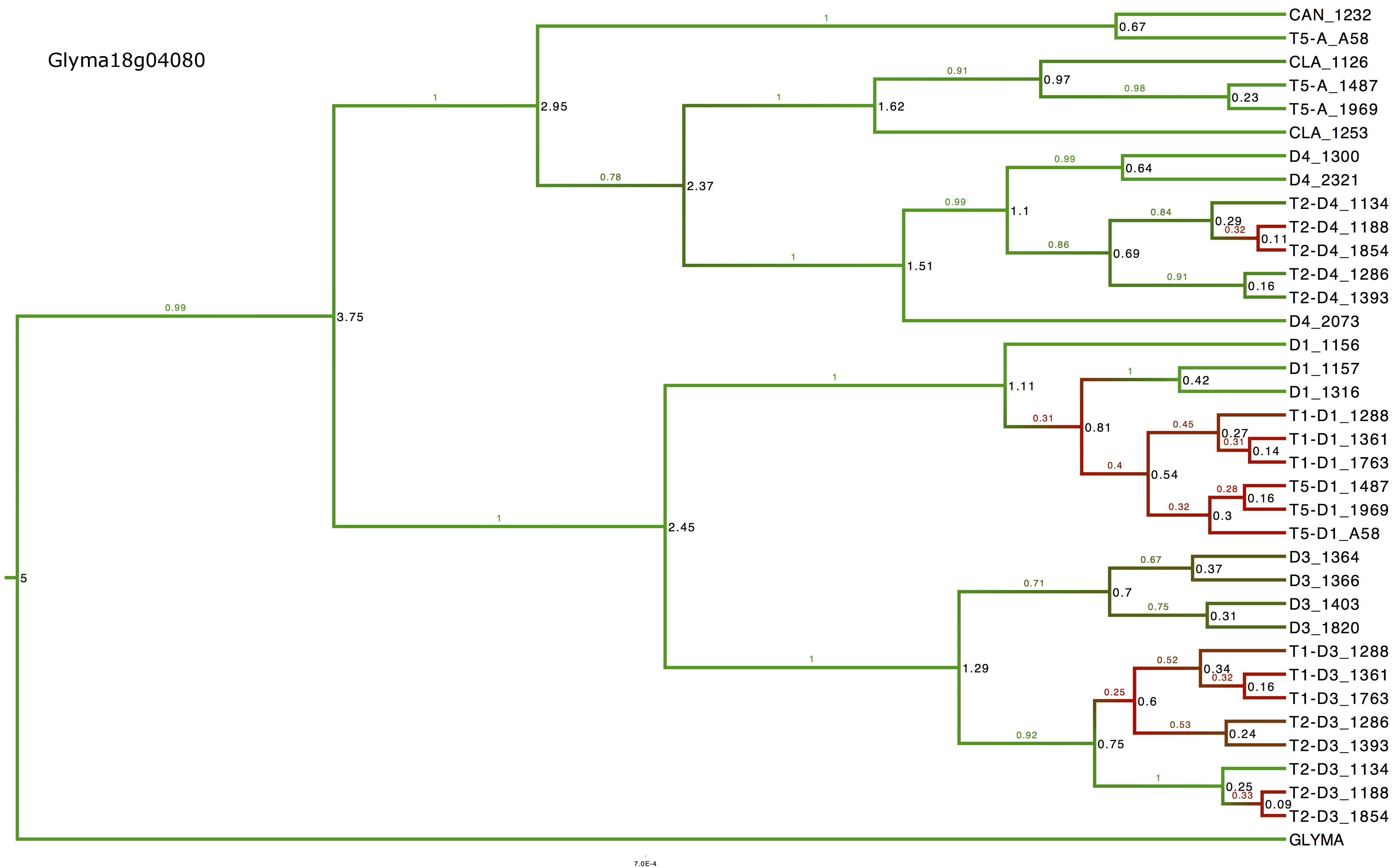

Supplement: Figures S41--S67 — The color of the tree branch represents the posterior probability value (also with the same color). Genes are in the following order: Glyma01g35620 (Figure S41), Glyma02g11580 (Figure S42), Glyma03g29330 (Figure S43), Glyma03g36630 (Figure S44), Glyma04g39670 (Figure S45), Glyma05g05750 (Figure S46), Glyma05g09310 (Figure S47), Glyma05g26230 (Figure S48), Glyma05g37840 (Figure S49), Glyma06g18640 (Figure S50), Glyma07g03370 (Figure S51), Glyma07g17180 (Figure S52), Glyma10g42100 (Figure S53), Glyma11g13880 (Figure S54), Glyma11g33720 (Figure S55), Glyma12g04150 (Figure S56), Glyma12g12230 (Figure S57), Glyma13g17820 (Figure S58), Glyma14g03500 (Figure S59), Glyma16g00410 (Figure S60), Glyma16g01980 (Figure S61), Glyma16g04940 (Figure S62), Glyma18g04080 (Figure S63), Glyma19g03390 (Figure S64), Glyma19g32940 (Figure S65), Glyma20g24930 (Figure S66), Glyma20g32930 (Figure S67). [file peerj-02-391-s003.zip › Suplementary_Figures_S41-S67/Suplementary_Figure_63.png]

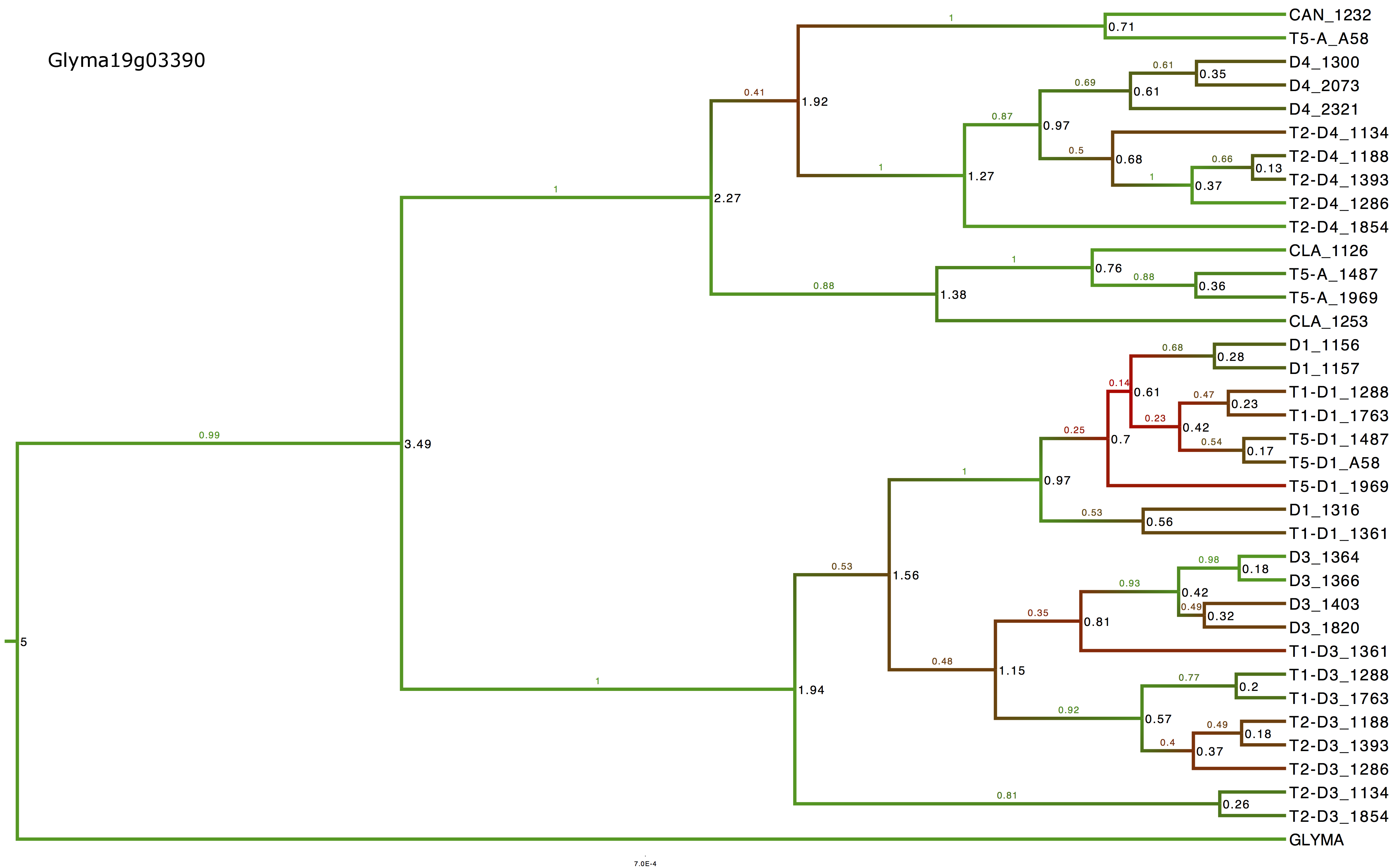

Supplement: Figures S41--S67 — The color of the tree branch represents the posterior probability value (also with the same color). Genes are in the following order: Glyma01g35620 (Figure S41), Glyma02g11580 (Figure S42), Glyma03g29330 (Figure S43), Glyma03g36630 (Figure S44), Glyma04g39670 (Figure S45), Glyma05g05750 (Figure S46), Glyma05g09310 (Figure S47), Glyma05g26230 (Figure S48), Glyma05g37840 (Figure S49), Glyma06g18640 (Figure S50), Glyma07g03370 (Figure S51), Glyma07g17180 (Figure S52), Glyma10g42100 (Figure S53), Glyma11g13880 (Figure S54), Glyma11g33720 (Figure S55), Glyma12g04150 (Figure S56), Glyma12g12230 (Figure S57), Glyma13g17820 (Figure S58), Glyma14g03500 (Figure S59), Glyma16g00410 (Figure S60), Glyma16g01980 (Figure S61), Glyma16g04940 (Figure S62), Glyma18g04080 (Figure S63), Glyma19g03390 (Figure S64), Glyma19g32940 (Figure S65), Glyma20g24930 (Figure S66), Glyma20g32930 (Figure S67). [file peerj-02-391-s003.zip › Suplementary_Figures_S41-S67/Suplementary_Figure_64.png]

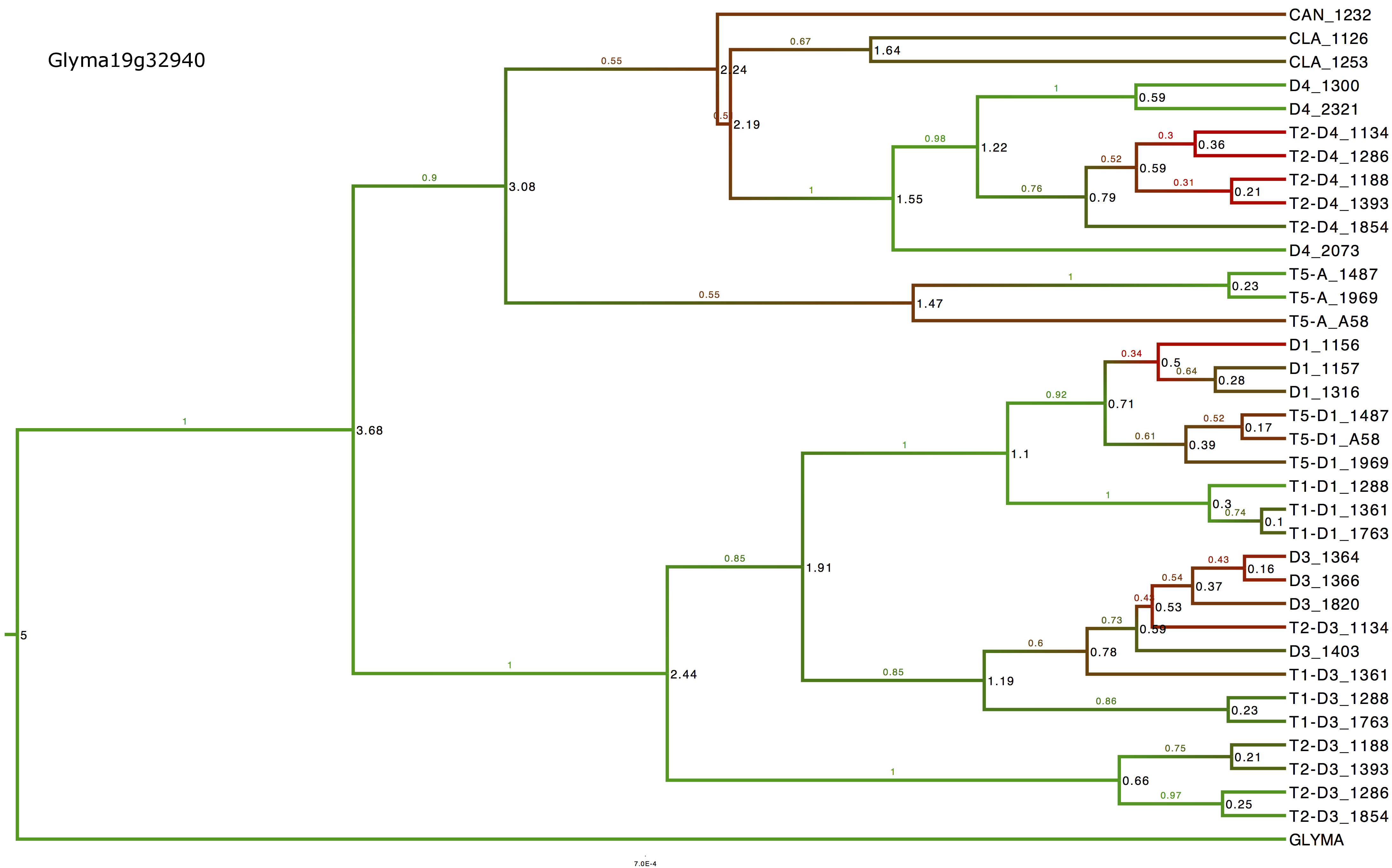

Supplement: Figures S41--S67 — The color of the tree branch represents the posterior probability value (also with the same color). Genes are in the following order: Glyma01g35620 (Figure S41), Glyma02g11580 (Figure S42), Glyma03g29330 (Figure S43), Glyma03g36630 (Figure S44), Glyma04g39670 (Figure S45), Glyma05g05750 (Figure S46), Glyma05g09310 (Figure S47), Glyma05g26230 (Figure S48), Glyma05g37840 (Figure S49), Glyma06g18640 (Figure S50), Glyma07g03370 (Figure S51), Glyma07g17180 (Figure S52), Glyma10g42100 (Figure S53), Glyma11g13880 (Figure S54), Glyma11g33720 (Figure S55), Glyma12g04150 (Figure S56), Glyma12g12230 (Figure S57), Glyma13g17820 (Figure S58), Glyma14g03500 (Figure S59), Glyma16g00410 (Figure S60), Glyma16g01980 (Figure S61), Glyma16g04940 (Figure S62), Glyma18g04080 (Figure S63), Glyma19g03390 (Figure S64), Glyma19g32940 (Figure S65), Glyma20g24930 (Figure S66), Glyma20g32930 (Figure S67). [file peerj-02-391-s003.zip › Suplementary_Figures_S41-S67/Suplementary_Figure_65.png]

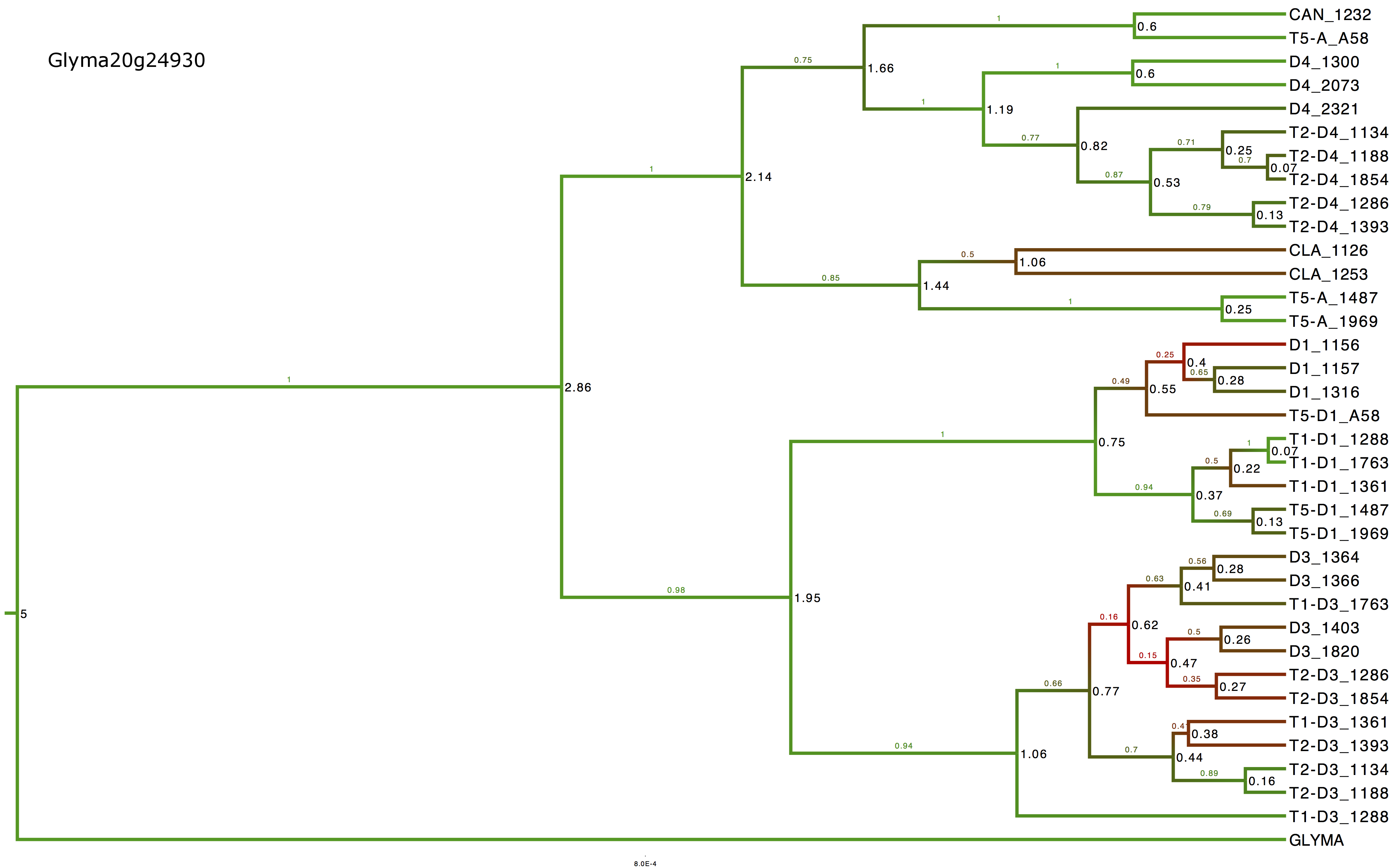

Supplement: Figures S41--S67 — The color of the tree branch represents the posterior probability value (also with the same color). Genes are in the following order: Glyma01g35620 (Figure S41), Glyma02g11580 (Figure S42), Glyma03g29330 (Figure S43), Glyma03g36630 (Figure S44), Glyma04g39670 (Figure S45), Glyma05g05750 (Figure S46), Glyma05g09310 (Figure S47), Glyma05g26230 (Figure S48), Glyma05g37840 (Figure S49), Glyma06g18640 (Figure S50), Glyma07g03370 (Figure S51), Glyma07g17180 (Figure S52), Glyma10g42100 (Figure S53), Glyma11g13880 (Figure S54), Glyma11g33720 (Figure S55), Glyma12g04150 (Figure S56), Glyma12g12230 (Figure S57), Glyma13g17820 (Figure S58), Glyma14g03500 (Figure S59), Glyma16g00410 (Figure S60), Glyma16g01980 (Figure S61), Glyma16g04940 (Figure S62), Glyma18g04080 (Figure S63), Glyma19g03390 (Figure S64), Glyma19g32940 (Figure S65), Glyma20g24930 (Figure S66), Glyma20g32930 (Figure S67). [file peerj-02-391-s003.zip › Suplementary_Figures_S41-S67/Suplementary_Figure_66.png]

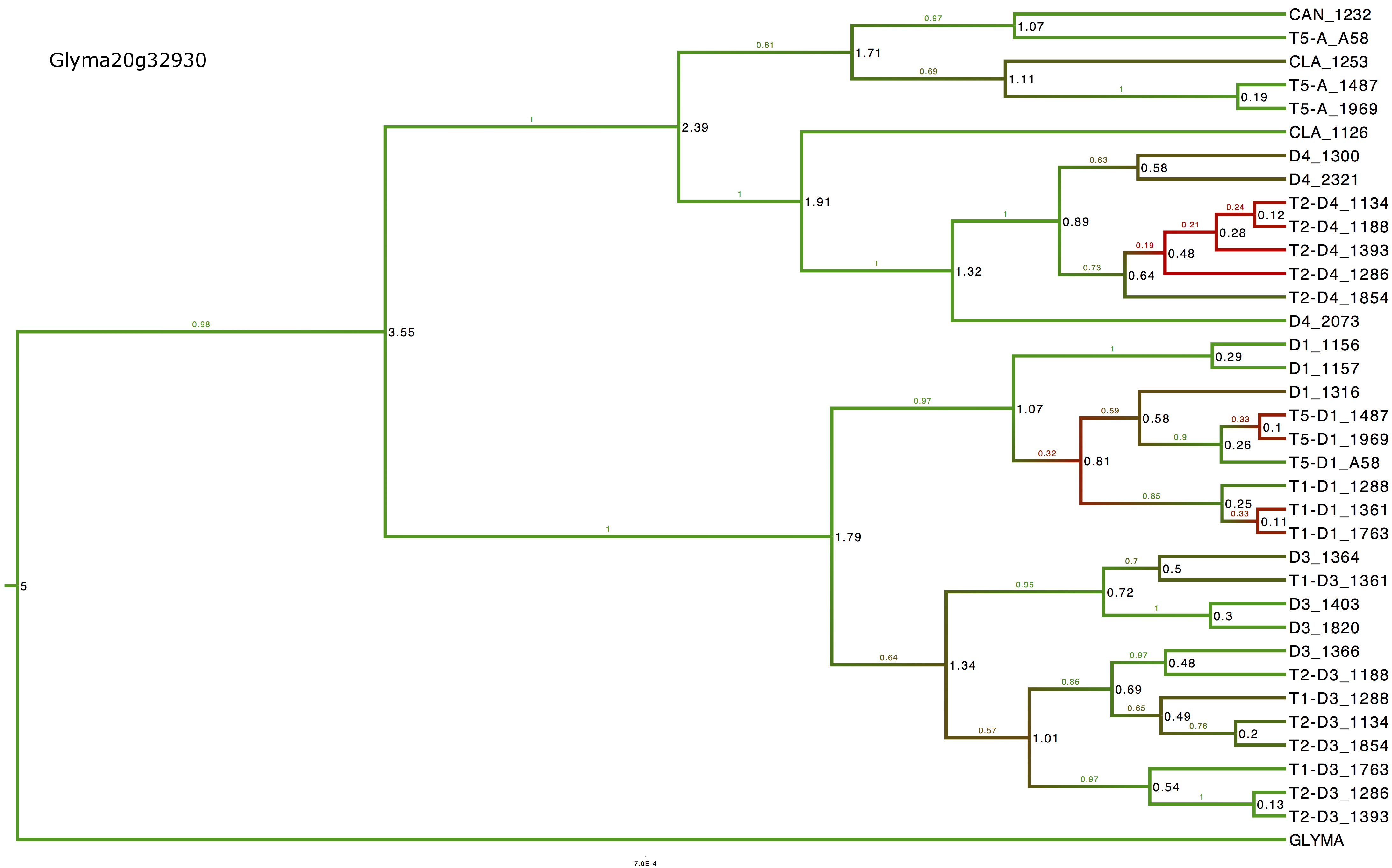

Supplement: Figures S41--S67 — The color of the tree branch represents the posterior probability value (also with the same color). Genes are in the following order: Glyma01g35620 (Figure S41), Glyma02g11580 (Figure S42), Glyma03g29330 (Figure S43), Glyma03g36630 (Figure S44), Glyma04g39670 (Figure S45), Glyma05g05750 (Figure S46), Glyma05g09310 (Figure S47), Glyma05g26230 (Figure S48), Glyma05g37840 (Figure S49), Glyma06g18640 (Figure S50), Glyma07g03370 (Figure S51), Glyma07g17180 (Figure S52), Glyma10g42100 (Figure S53), Glyma11g13880 (Figure S54), Glyma11g33720 (Figure S55), Glyma12g04150 (Figure S56), Glyma12g12230 (Figure S57), Glyma13g17820 (Figure S58), Glyma14g03500 (Figure S59), Glyma16g00410 (Figure S60), Glyma16g01980 (Figure S61), Glyma16g04940 (Figure S62), Glyma18g04080 (Figure S63), Glyma19g03390 (Figure S64), Glyma19g32940 (Figure S65), Glyma20g24930 (Figure S66), Glyma20g32930 (Figure S67). [file peerj-02-391-s003.zip › Suplementary_Figures_S41-S67/Suplementary_Figure_67.png]

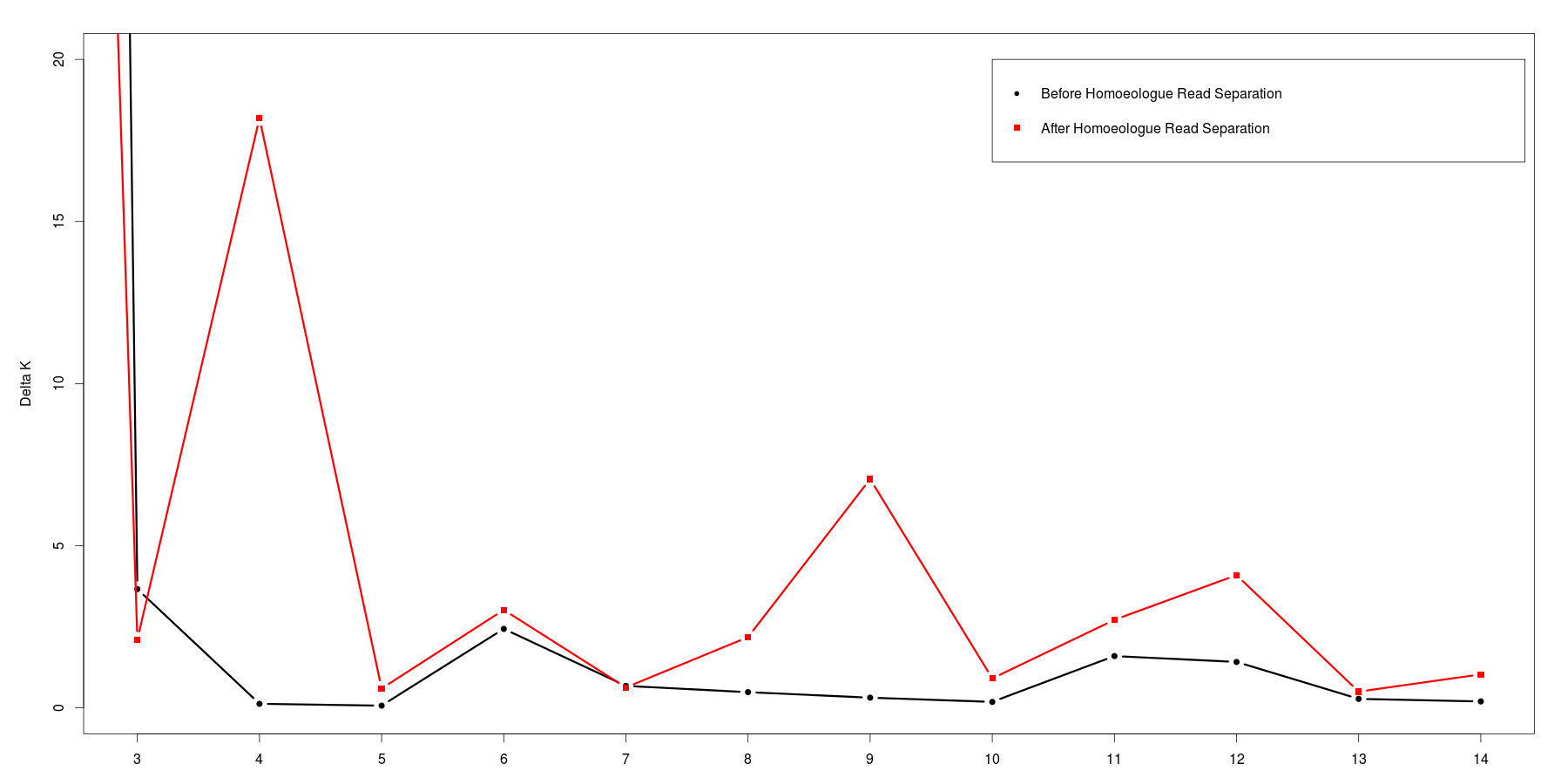

Supplement: Figure S11 — Delta K graph (Evano et al. 2005) for Structure analysis for different K (from 3 to 15) before and after the homoeologues separation. [file peerj-02-391-s004.png]

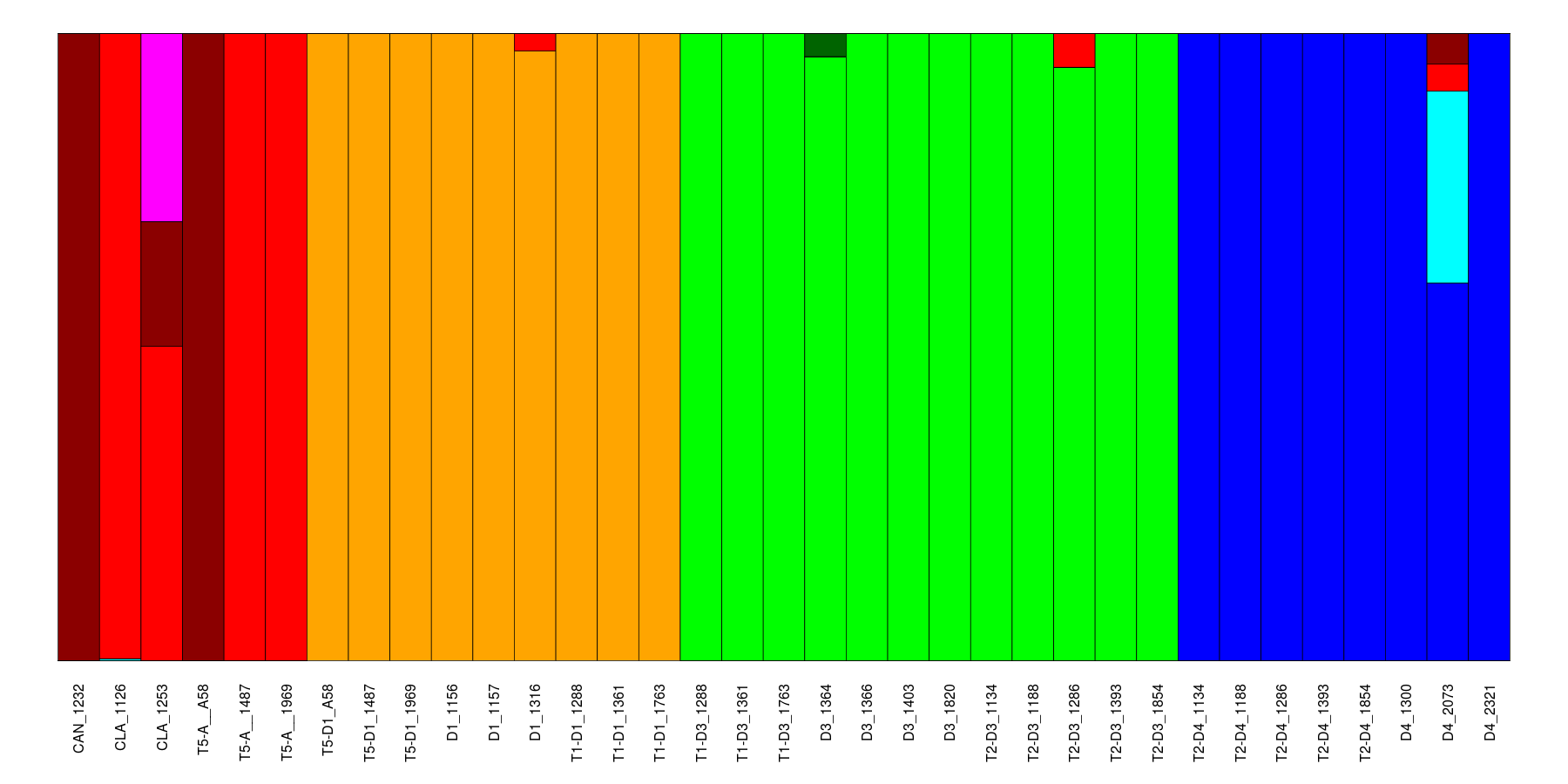

Supplement: Figure S12 — Structure results after homoeologues separation for K = 9 [file peerj-02-391-s005.png]

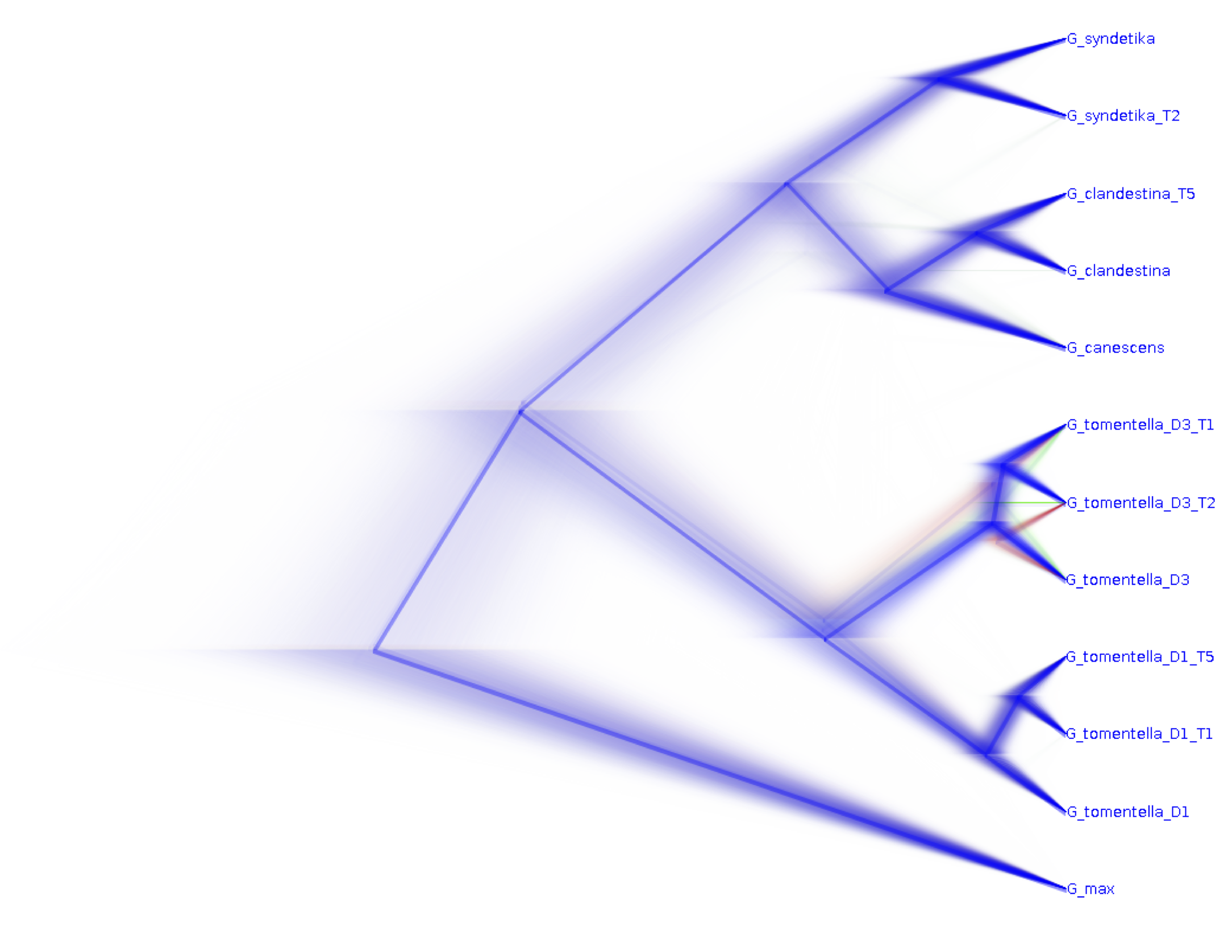

Supplement: Figure S40 — Densitree for the *BEAST analysis representing 10,000 trees (sampling each 1,000 for the MCMC). [file peerj-02-391-s006.png]
